# Assessing and improving on-farm biosecurity knowledge and practices among swine producers and veterinarians through online surveys and an educational website in Illinois, United States

**DOI:** 10.3389/fvets.2023.1167056

**Published:** 2023-06-09

**Authors:** Isha Agrawal, Corinne Bromfield, Csaba Varga

**Affiliations:** ^1^Department of Pathobiology, College of Veterinary Medicine, University of Illinois Urbana-Champaign, Urbana, IL, United States; ^2^Agriculture and Environment Extension, College of Agriculture, Food, and Natural Resources, University of Missouri, Columbia, MO, United States; ^3^Carl R. Woese Institute for Genomic Biology, University of Illinois Urbana-Champaign, Urbana, IL, United States

**Keywords:** United States, Illinois, swine, biosecurity, veterinarians, farmers, foreign animal disease, Google analytics

## Abstract

There is a growing risk to the health and productivity of the Illinois and United States swine population from foreign and endemic infectious diseases. Effective on-farm biosecurity practices play a pivotal role in preventing these high-consequence pathogens from affecting swine farms. Veterinarians are essential in providing disease prevention advice to swine producers that can help them implement effective biosecurity practices on their farms. Our descriptive study objectives were to assess Illinois swine producers’ and veterinarians’ biosecurity perception, knowledge, and practices to identify knowledge gaps and address these by developing an online educational website. We developed two independent online questionnaires using Qualtrics^XM^ software. Swine producer members of the Illinois Pork Producers Association and veterinarians registered with the Illinois State Veterinary Medical Association were contacted via e-mail through their associations and asked to complete an online survey. In total, 13 swine producers across 9 Illinois counties operating 82 farms (8 managed single farms and 5 managed multiple farms) responded to the swine producer survey. Despite some biosecurity awareness among swine producers, the need for a biosecurity-related outreach program was evident. Among the 7 swine veterinarian responders, 5 predominantly treated swine (oversaw an average of 21.6 farms), and 2 were mixed animal practitioners. The swine veterinarian survey showed a disconnect between their biosecurity perception and practices. We developed a biosecurity educational website and used Google Analytics to collect website traffic and user data. The 4 months of data showed good coverage that included the highest proportion of users from the Midwest and North Carolina, the largest swine-production regions in the US, and China and Canada, the leading producers of swine worldwide. The most accessed webpage was the resources page, and the swine diseases page had the highest engagement time. Our study highlights the effectiveness of combining online surveys with an educational website to assess and improve the biosecurity knowledge of swine producers and veterinarians that can be applied to assess and improve the biosecurity knowledge and practices of other livestock farmers.

## 1. Introduction

The United States of America (US) is a leading producer of pork and pork products (1). The entry and spread of pathogens causing foreign animal diseases into and among the US swine farms and the emergence of endemic infectious diseases would threaten the productivity and profitability of the swine sector and would negatively impact the agricultural economy (2, 3). On-farm biosecurity knowledge, plans, and associated practices are crucial to prevent the introduction and spread of these high-consequence pathogens into swine farms (2, 3).

Pork production is a vital component of the US agricultural economy, contributing to an estimated $35.86 billion of personal income and $57.20 billion of the gross national product in 2021 (4). Within the US, the Midwestern states (Iowa, Minnesota, Indiana, Illinois, Missouri) and North Carolina are leaders in pork production. Illinois ranks fourth with an annual output of $1.91 billion of marketed hogs and 5.4 million heads in December 2021 (5). The US swine industry has changed significantly in the last few decades, shifting from a small-scale swine production system of numerous farms, each with fewer pigs, to an integrated system of fewer farms with a large number of pigs (6). In Illinois, larger farms with 5,000 or more hogs are predominant, making up 76% of the total swine inventory, while farms with 2,000 to 4,999 heads represent 18%, and farms with less than 2,000 heads constitute only 6% (7). The integrated multi-site swine production system requires more within and between farm movements of animals and people, posing new challenges in maintaining disease-free farms (8–10). Moreover, the emergence and re-emergence of infectious and economically devastating swine diseases in the US, like Porcine Reproductive and Respiratory Syndrome (PRRS) (11), Porcine Epidemic Diarrhea (PED) (12), and the looming threat of African swine fever (ASF) (13), adds to the risks to the US swine industry. Some of these diseases can be prevented or controlled by vaccination; however, in the US, there is no commercially available vaccine or treatment against foreign animal diseases like ASF (14). The gravity of the consequences of these disease outbreaks in the US highlights the importance of protecting the swine herds by adopting disease prevention practices.

The “minimal disease approach” theory, which aims to produce healthy pigs economically, first described biosecurity in swine production around the 1980s (15). The concept of on-farm biosecurity categorizes these procedures into two components: external biosecurity, which consists of measures aimed at preventing the introduction of a disease into a farm, and internal biosecurity, which involves practices intended at controlling the spread of already present diseases within a farm (3). Previous studies described the importance of biosecurity in disease prevention and control and highlighted the benefits of adopting effective biosecurity plans and practices for swine producers (2, 3, 16–20). However, a lack of knowledge about these practices feeds the resistance to their adoption. Therefore, to encourage the adoption of biosecurity plans and practices, it is essential to understand the perception and beliefs of swine producers (21), as the Theory of Planned Behavior suggests that human behaviors and practices are belief-driven (21–23).

Livestock veterinarians play a crucial role in ensuring animal health and welfare by treating farm animals, addressing producers’ concerns on health management, advising farmers on disease prevention, and assessing the effectiveness of on-farm biosecurity practices (3, 24). Effective on-farm biosecurity requires continuous assessment and improvements of plan and practice that requires a veterinarian’s recommendations and expertise (25). Previous studies have shown the significance of educating farmers on effective disease prevention and control practices for maintaining farm biosecurity (17). Moreover, educating swine producers on diseases and biosecurity measures is vital for shaping a positive perspective toward biosecurity (16, 18).

Using a web-based platform as an educational tool deems fit in this age of digitalization, where access to the Internet has become imperative for a diverse set of industries, including agriculture and livestock production (26, 27). An educational website provides opportunities for educators to evaluate, improve, and update the provided information over time based on the users’ needs (28). Adding Google Analytics to these web-based education tools is a cost-effective way of assessing the effectiveness of user engagement and website outreach (29) Google Analytics is a valuable tool that enables user data collection and website traffic monitoring and is a free hosted service that uses JavaScript to collect data (30). Previous studies have used Google Analytics to collect website traffic data to evaluate the effectiveness of their website and enhance the web-based learning experience for their users (31, 32). Website traffic data gives information on the most and the least visited pages, the number of downloads, and the time spent per page. In addition, Google Analytics records information on users’ demographics, geographic locations, and devices used to access the website (31, 32).

Encouraging swine producers to adopt farm-level biosecurity plans and practices and their continuous evaluation is an ongoing challenge (3). There is a lack of literature on disease risk perception, foreign animal disease preparedness, and biosecurity knowledge of swine producers and veterinarians in Illinois and the US. No previous study has evaluated Illinois swine producers and veterinarians on their biosecurity knowledge and perception. As a leading pork-producing state, evaluating Illinois swine farmers’ and veterinarians’ biosecurity practices and understanding their infectious disease risk perception is imperative. With this aim, the first study objective is to assess Illinois swine producers for their knowledge and perceived risk of foreign and endemic diseases, evaluate biosecurity practices implemented on their hog farms, and assess their preparedness for a potential foreign animal disease outbreak. Given the significance of swine veterinarians in swine health management, the second objective of our study is to evaluate swine veterinarians’ biosecurity knowledge, perception, and practices and explore their relationship with their swine producer clientele. The third study objective is to develop an online biosecurity educational website to address the knowledge gaps identified during the surveys and evaluate the effectiveness of the biosecurity website as an educational tool.

## 2. Methods

**Figure 1:**
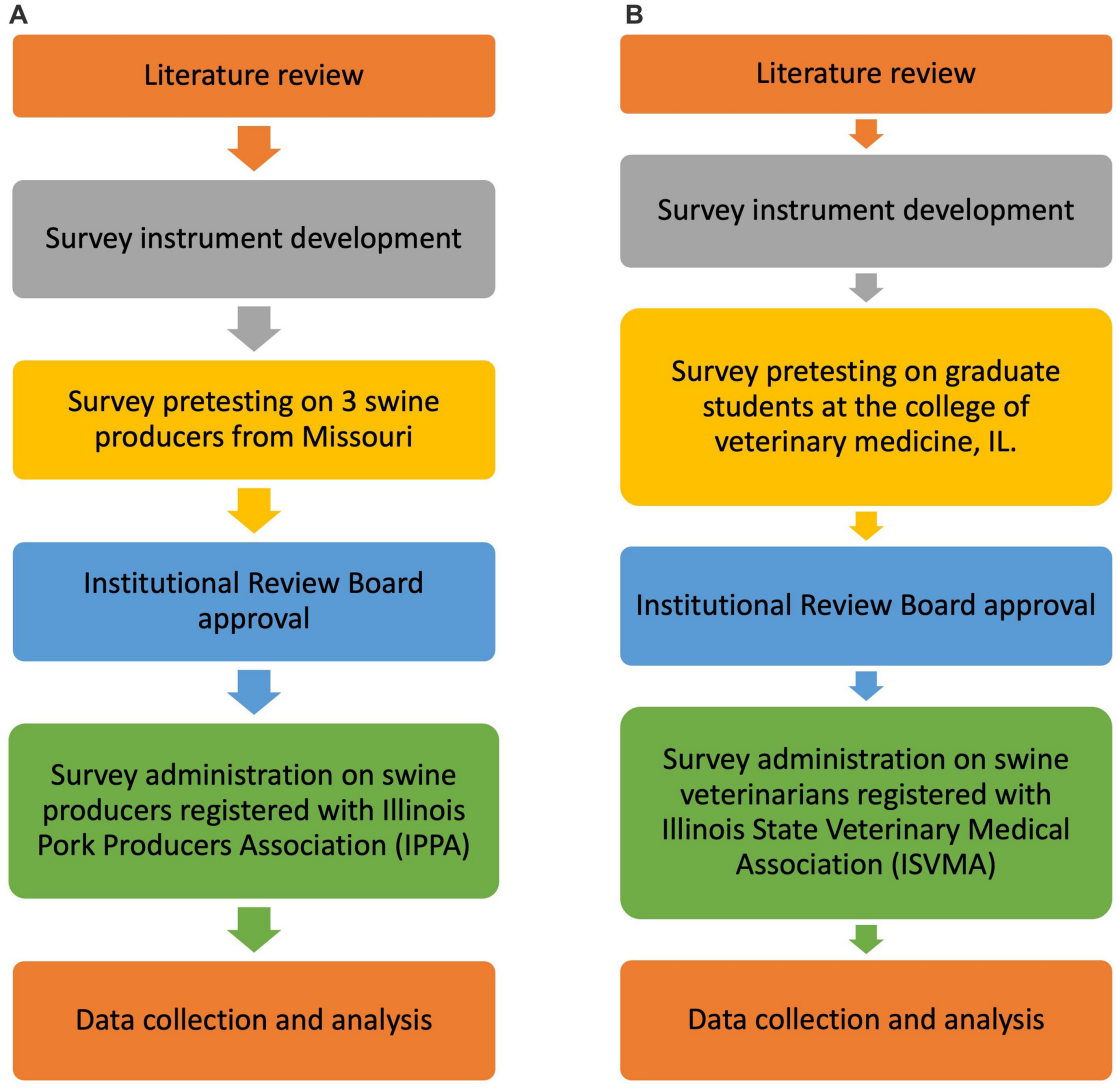
Description of the design, administration, and analysis of the swine producer **(A)** and swine veterinarian **(B)** surveys.

The survey design and administration protocols followed for swine producers’ and veterinarians’ surveys are described in Figure 1.

### 2.1. Ethics statement

The Institutional Review Board (IRB) of the University of Illinois at Urbana Champaign approved the survey instruments and the study procedure for the swine producers and swine veterinarians surveys (IRB #21974). We complied with the set guidelines for the study methodology and data collection. The surveys received an exempt status because participants remained anonymous and they had to complete a consent form at the beginning as a prerequisite to participate in the survey.

### 2.2. Survey of swine producers

#### 2.2.1. Sampling frame, questionnaire development, and pretesting

The sampling frame consisted of swine producers registered with the Illinois Pork Producers Association (IPPA).

The study team developed the questionnaire consulting existing literature on biosecurity practices and evaluation (2, 18, 33–38) and by seeking advice from experts in swine biosecurity and survey research methodology from Illinois. Additionally, two board members from the IPPA reviewed the questionnaire and provided feedback. Lastly, three swine producers from the state of Missouri pretested the questionnaire. Pretesting enabled us to check the survey flow and identify potential problems in the survey instrument and its relevance to swine production in Illinois. Based on the comments received from the experts and swine industry stakeholders, the study team modified and finalized the questionnaire.

#### 2.2.2. Questionnaire characteristics and survey administration

We designed a concise and interactive survey that took the responders approximately 15 min to complete. The number of questions in the survey ranged from 98 to 116, depending on the answer choice of the swine producers. Questions not relevant to the responders were skipped using the skip logic attribute of Qualtrics^XM^ software. The questionnaire comprised a combination of closed and open-ended questions divided into nine sections (Supplementary File 1). The sections were as follows: (i) demographics characteristics and general production-related questions (11 questions), (ii) disease risk perception and biosecurity knowledge (10 questions), (iii) farm characteristics tailored to single or multiple farm owners (17–20 questions), (iv) farm practices related to the movement of people (36 questions), (v) farm practices related to the movement of animals (13 questions), (vi) animal health management (6 questions), (vii) previous disease outbreak on their farms (3 questions), (viii) a section for multiple farm owners regarding farm characteristics of their other farms (15 questions), and (ix) a feedback section to learn about the preferred source of disease prevention information of the farmers (2 questions).

The questionnaire was forked based on the number of farms swine producers owned. Farmers who owned multiple farms in Illinois would first respond to the survey questions about their newest farm, and these responders would complete an additional section about their other farms. We developed this section to identify differences in biosecurity-related structures, farm management, and disease history among their newest and other farms. We used the selective display attributes of Qualtrics software to facilitate easy forking based on survey participants’ responses.

The survey was distributed via a personal e-mail through the IPPA in June 2021 to 406 registered IPPA members whose e-mail addresses were available. We enclosed the e-mail with a cover letter that contained the survey link. An e-mail reminder was sent 2 weeks after the initial survey launch. We kept the survey link active for 4 weeks.

### 2.3. Survey of swine veterinarians

#### 2.3.1. Sampling frame, questionnaire development, and pretesting

The sampling frame consisted of all veterinarians registered with the Illinois State Veterinary Medical Association (ISVMA).

The study team developed the first draft of the questionnaire consulting previous studies (24, 36, 37, 39–42). A veterinarian expert in swine biosecurity in Illinois reviewed the questionnaire. We completed the questionnaire pretesting with 24 graduate students from the College of Veterinary Medicine, University of Illinois, Urbana-Champaign. Most of the graduate students who enrolled in pretesting had a veterinary degree. They checked the survey instrument for validity and errors in survey flow. The questionnaire was revised based on the comments received.

#### 2.3.2. Questionnaire design and survey administration

We designed a short, interactive survey that took approximately 10 min to complete. The survey comprised 45 to 47 closed and open-ended questions divided into six sections (Supplementary File 2). The range of questions in the questionnaire (43–45) implied the use of the skip logic attribute of Qualtrics^XM^ to skip questions that were not relevant to the respondent based on his/her answer choices in the previous questions. The six sections in the questionnaire were as follows: (i) general demographic characteristics (7 questions), (ii) disease risk perception and knowledge (10 questions), (iii) disease investigation and reporting (3 questions), (iv) demographics of clinical veterinarians (5 questions), (v) biosecurity practices of clinical veterinarians tailored to their type of practice (18–20 questions), and (vi) sources of updated biosecurity information (2 questions). The questionnaire was forked based on the type of practice using the Qualtrics software’s selective display features. In addition, we included practice-related questions tailored to the practice type (companion animal veterinarian, swine veterinarian, bovine veterinarian, equine veterinarian, and mixed animal veterinarian) for the veterinarians in clinical practice.

In September 2021, ISVMA distributed the survey to all active ISVMA members (1,391 veterinarians) via personal e-mails. The survey was kept open for 8 weeks. During these 8 weeks, ISVMA sent out two personal e-mail reminders and shared the survey link twice in their weekly online newsletter. At the end of the survey, we received 121 responses. For this study, we included only veterinarians exclusively or predominantly working in swine clinical practice (*n* = 7 swine veterinarians).

### 2.4. Swine biosecurity educational website development

We designed a swine biosecurity website (14) as an educational tool to address the biosecurity knowledge and practice gaps among Illinois swine producers and veterinarians identified in the surveys. The website contains information on swine diseases and on-farm biosecurity, divided into six modules. The modules covered the following topics: (i) high-consequence swine diseases, (ii) a brief overview of biosecurity, (iii) a description of general biosecurity practices, (iv) a description of external biosecurity, (v) elements of internal biosecurity, and (vi) details of the Secure Pork Supply Plan. An editor translated each module’s content into simple language devoid of jargon. At the end of each module, we included a short quiz to allow the users to “Test their Knowledge” (Supplementary File 3). A consent form was attached with each quiz to request permission from the users to use their quiz result data in an anonymous form for research. Additionally, we created a resources section on the website with useful biosecurity information sheets, including seven Infographics (Supplementary File 4) and seven usable signs in a downloadable format.

#### 2.4.1. Launching Google analytics to track the swine biosecurity website

We set up Google Analytics for the swine biosecurity website to collect website traffic and user data to evaluate the biosecurity outreach and its efficacy as an educational tool. We recorded numerous variables related to website acquisition and engagement, user demographics, and technology used. For this study, we evaluated 4 months of data starting from July 5th, 2022, the day of the website launch and promotion, till November 5th, 2022.

### 2.5. Statistical analysis

At the end of both surveys, we exported the data from Qualtrics^XM^ to Excel (.xlsx) and comma-separated values (.csv) formats. We stored the raw data in CSV format in cloud storage and used duplicate Excel files for data cleaning and recoding. We used STATA Intercooled software (Version 17, Stata Corporation, College Station, TX) for data analysis and R Studio (Version 1.4.1106 2009–2021 RStudio, PBC) for data visualization using packages ggplot2 and tidyverse. We generated descriptive data for all variables and presented them in tables and charts. We calculated the mean for continuous variables and proportions for binomial and categorical variables. In addition, we recategorized the 5-scale Likert scale into 3-scale Likert scale variables, and proportions were reported. Finally, we reported the frequency and proportion of perceptions and practices of swine producers owning single and multiple farms. We generated only descriptive data from the veterinarians’ survey reporting frequency and proportion for all variables.

## 3. Results

### 3.1. Survey of swine producers

We received 16 responses from Illinois swine producers, and 13 producers who completed the survey were included in the analysis.

#### 3.1.1. Demographics of swine producers

All 13 responders were male, with a mean age of 57 years. Ninety-two percent of the producers had more than 25 years of experience in swine production (Table 1). Nine of the responders reported having a 4 year college or bachelor’s degree (Table 1).

**Table 1:** Demographic characteristics of swine producers.

Variable	Frequency (%)
Business Structure (*N* = 13)	
Independent producer	7 (54)
Contractor or integrator	2 (15)
Contract hog producer	4 (31)
Others	0
Education (*N* = 13)	
Did not complete high school	0
High school or equivalent	2 (15)
Some college or Associates degree	2 (15)
4-year college or bachelor’s degree	9 (70)
Graduate degree	0
Years of experience in hog production^a^ (*N* = 13)	
<25 years	1 (8)
>25 years	12 (92)
Role at hog farm^b^ (*N* = 13)	
Farm owner	13 (100)
Farm manager	5 (38)
Veterinarian	0
Others	0
Multiple hog farms operated in IL (*N* = 13)	
Yes	5 (38)
No	8 (62)

a Indicates a categorical variable that has been recategorized into two categories.

b Indicates a multiple-answer question with some responders having multiple roles on the farm.

Sixty-two percent (*n* = 8) of the swine producers operated a single swine farm, and the remaining (5 producers) operated 74 farms across Illinois. Thirteen producers who completed the survey managed 82 farms from 9 counties across Illinois (Knox, Carroll, Ford, Montgomery, DeKalb, Monroe, Randolph, Effingham, and Lee). The predominant production type of farms was wean-to-finish (*n* = 66), followed by farrow-to-finish (*n* = 3), and farrow-to-wean (*n* = 3). Sixty-two percent (*n* = 8) of the responders raised only swine on their farm; five producers reported raising other animals along with swine (cattle (*n* = 2), poultry (*n* = 2), sheep (*n* = 1), goat (*n* = 1), llama (*n* = 1). All producers reported having a Premise ID on all of their farms, 67 swine farms implemented the Secure Pork Supply plan (46), and seven have had a biosecurity assessment done on their farms within the last 13–24 months. The five multiple farm owners representing 72 swine farms across Illinois reported having “not very different/slightly different” biosecurity plans and practices for the farms other than their newest farm.

#### 3.1.2. Swine producers’ perception of disease risk and biosecurity adoption

Most swine producers believed that biosecurity practices play an essential role in disease prevention and control; however, they had varying perceptions of foreign animal disease risk (Table 2). Four responders representing 62 swine farms in Illinois reported that a foreign animal disease outbreak “probably/definitely will not occur” in the US swine industry in the next 5 years (Table 2). Eighty-eight percent of the responders indicated they would contact a private veterinarian if they suspected an emergency foreign animal disease in their herd (Table 2). Only 33% of the farmers were aware of the government indemnity requirement for a biosecurity plan in case of a foreign animal disease outbreak.

**Table 2:** Biosecurity and Foreign Animal Disease risk perception-based questions asked to swine producers.

Perception questions	*n* (%)
How important do you consider developing an enhanced disease prevention and control plan (e.g., Secure Pork Supply Plan) that can be used during a foreign animal disease (FAD) outbreak for your hog farm?^a^	
Extremely Important/Very Important	11 (84)
Moderately important	1 (8)
Slightly important/Not at all Important	1 (8)
How important do you consider routine testing for the detection and prevention of specific diseases on your hog farm(s)?^a^	
Very Important/Important	11 (84)
Moderately important	2 (16)
Slightly important/Not at all Important	0 (0)
What do you think is the likelihood of the occurrence of a foreign animal disease (FAD) outbreak in the next 5 years in the US swine industry?^a^	
Definitely will occur/Probably will occur	4 (29)
Possibly will occur	6 (42)
Probably will not occur/Definitely will not occur	4 (29)
How would you rate your hog operation’s disease prevention and control measures?^a^	
Very good/good	10 (77)
Acceptable	3 (23)
Poor/Very poor	0 (0)
How beneficial do you think your biosecurity measures are at preventing disease introduction into your hog farm?^a^	
Extremely beneficial/Beneficial	14 (88)
Moderately Beneficial	2 (12)
Somewhat Beneficial/Not beneficial at all	0 (0)
Where do you currently spend most of your money out of your farm health management funds?	
Prevention of diseases	12 (75)
Treatment of diseases	4 (25)
If given a choice, which do you generally think is more cost-effective?	
Preventing diseases from infecting your animals	15 (94)
Treating your animals when they have a disease	1 (6)
Are you familiar with foreign animal diseases (FAD) such as African swine fever (ASF) / Foot and mouth disease (FMD)?^a^	
I have full/decent knowledge of these diseases	13 (81)
I have a superficial knowledge of these diseases	3 (19)
I have just heard/never heard about these diseases	0 (0)
If you suspect an emergency foreign animal disease (FAD) in your herd, who are you most likely to contact?	
I would not call anybody	0
Private veterinarian	14 (88)
Illinois State veterinarian	0 (0)
Extension Agent	0 (0)
Neighbor	0 (0)
Other	2 (12)
If a foreign animal disease (FAD) outbreak occurred on your farm, what do you think the government indemnity payment approach would be?	
No indemnity payments will be available	3 (23)
Indemnity payments will be available for all farmers regardless of their disease prevention and control efforts	4 (31)
Indemnity payment will be available only for farmers who demonstrate and document disease prevention and control efforts	2 (15)
Indemnity payment will be available only for farmers who are registered with SAM and have a DUNS number	4 (31)
If a foreign animal disease (FAD) outbreak occurred on your farm, in your opinion, how long would the negative impacts on your operation persist?	
less than 1 month	0 (0)
1–2 months	0 (0)
3–6 months	3 (23)
6–12 month	2 (15)
more than 12 months	8 (62)

a Five-scale choices were collapsed and reported as three-scale.

#### 3.1.3. Biosecurity structures of swine farms

More than 85% of the farms had a clear demarcation between dirty and clean areas at the farm entrance. Eighty-two percent (*n* = 67) of the farms had shower facilities at their farm entrance for shower-in and shower-out for visitors and farm personnel entering the farm (Table 3).

**Table 3:** Barn-entry system and biosecurity-related structures and practices adopted by swine producers on their farms (*N* = 82) across Illinois.

Structures related to the barn entry system present on the farm	*n* (%)
Footbath at the barn entry	2 (2)
A dirty area that holds dirty clothes and boots	72 (88)
A clean area to put on farm-specific coveralls/boots	74 (90)
A clear demarcation (e.g., bench, counter) between dirty and clean areas	71 (87)
Hand-wash basin in the dirty area	72 (88)
Hand-wash basin in the clean area	67 (82)
Toilet	72 (88)
Shower	67 (82)

Biosecurity related structures	*n* (%)
Designated parking area for trucks and visitors	68 (83)
Loading and unloading area for pigs	75 (92)
Delivery areas for feed	68 (83)
Cleaning and disinfection station	60 (73)
Perimeter buffer area (PBA)	9 (11)
Line of separation (LOS)	79 (96)
Signages at the farm entrance	76 (93)
Gates and barriers	6 (7)

Biosecurity-related practices	*n* (%)
Logbook entry at the farm entrance	72 (88)
Separate entrance for waste-collecting vehicles	69 (84)
Vehicles restricted to entering within 10 feet radius of barns	81 (99)
Require vehicles to be washed and disinfected before entry into the farm	66 (81)
Change into farm-specific clothes and boots before entry into the farm	80 (98)
Showering before entering and exiting the farm	72 (88)

#### 3.1.4. Biosecurity practices at swine farms

Responders reported the farm and health management practices followed at their swine farms to maintain farm biosecurity. All 82 farms had active rodent control; however, 74 farms had wildlife exclusion/control facilities, and 73 farms had bird exclusion/control and restricted pet entry on their farms. Eighty-six percent of the producers get feed delivered from the mill, whereas 12% of the producers reported self-production of feed. Seventy-five percent of the responders reported using surface water on their farms, and the remaining producers get well (19%) or public water supply (6%) on their farms. On-site manure storage (*n* = 78) and land application (*n* = 78) were the most popular means of manure disposal. Other methods include on-site composting (*n* = 67) and land application on other farms (*n* = 70). On-site composting (*n* = 78) was the primarily reported method of dead stock disposal. Other methods include on-site burial (*n* = 2), on-site incineration (*n* = 1), and off-site rendering (*n* = 3).

**Figure 2:**
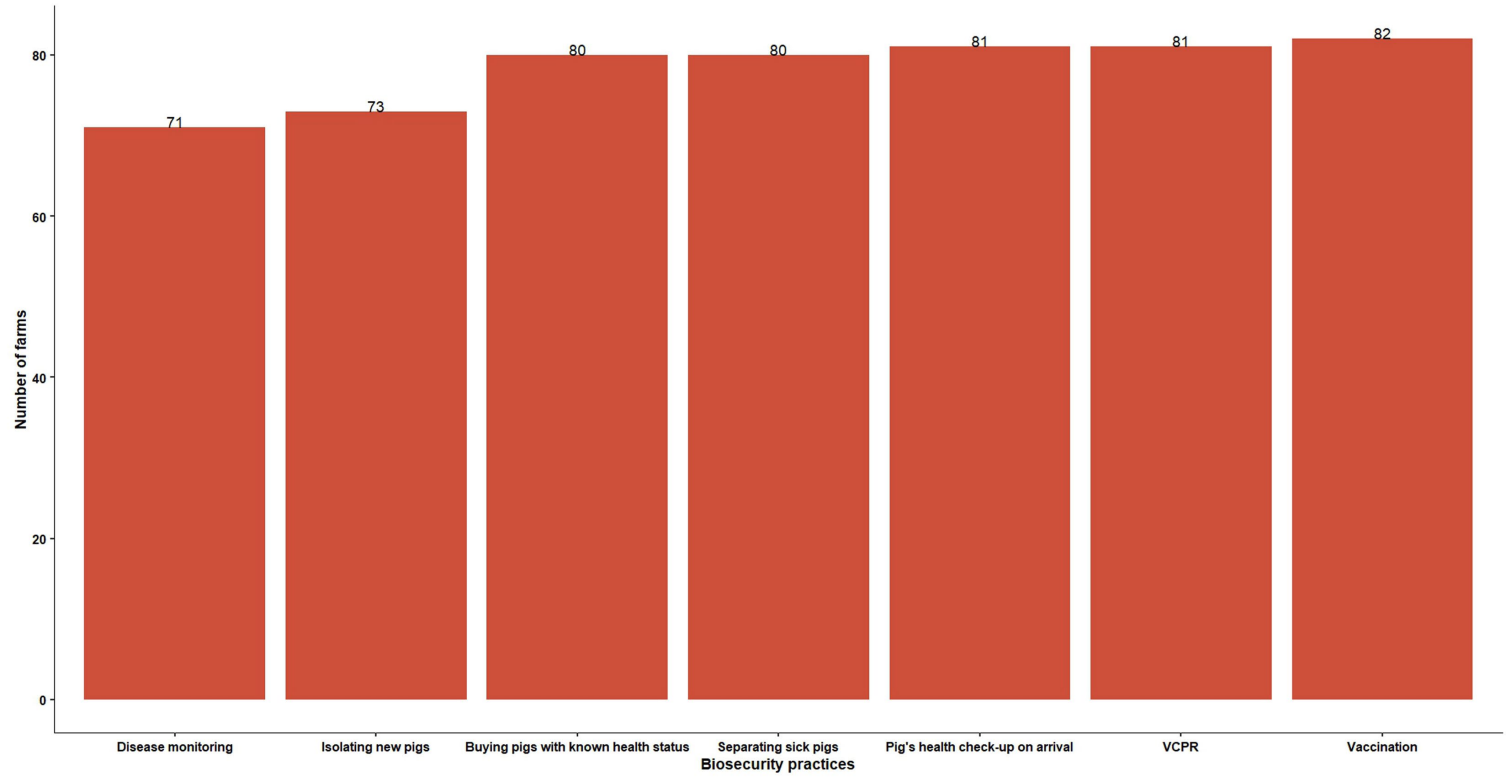
Health management practices adopted on swine farms (VCPR-Veterinarian-Client-Patient-Relationship).

The least commonly reported health management practices were testing pigs for diseases and isolating new animals on arrival on the farm (Figure 2). Among biosecurity practices adopted on the farms, restricting vehicles from entering within 10 feet radius of the barn (99%), and requiring the vehicles entering the farm to be washed and disinfected (81%) were most commonly adopted (Table 3).

Swine producers reported disease outbreaks on their farms in the last 3 years. Responders (*N* = 13) reported PRRS (*n* = 4) and swine influenza (*n* = 3), as the most common disease outbreak that occurred in the last 3 years on their farms. Apart from this, PED (*n* = 2), Mycoplasmosis (*n* = 1), and Seneca Valley Virus (*n* = 1) outbreaks were also reported by the responders.

### 3.2. Survey of swine veterinarians

#### 3.2.1. Demographics of swine veterinarians

From the cross-sectional survey of all Illinois veterinarians, responses from 7 swine veterinarians (5-predominantly swine and 2-mixed animals) were included in the study (Table 4). The exclusive swine veterinarians had a mean clientele of 21.6 farms (range: 10–33). Of the seven veterinarians, 4 were males, and 3 were females. One of seven swine veterinarians had board certification. Eighty-six percent of the responders were from central Illinois. Six of seven swine veterinarians were owners of the clinical practice, and one of seven was an associate veterinarian. Fifty-seven percent of the veterinarians had more than 15 years of experience treating swine. Five of seven veterinarians received biosecurity training after completing their veterinarian degree. All these veterinarians received their biosecurity training between the years 2019–2021 through “In-person training” (5 veterinarians),” Continuing Education credits” (*n* = 2), “webinars” (*n* = 4), and “phone, printed media, e-mails” (*n* = 1).

**Table 4:** Demographic characteristics of swine veterinarians in Illinois.

Demographics	*n* (%)
Type of practice	
Exclusively/predominantly swine	5 (71)
Mixed animal	2 (29)
Gender of responders	
Male	4 (57)
Female	3 (43)
DVM graduation year^a^	
1944–1974	1 (14)
1975–2004	3 (43)
2005–2021	3 (43)
Additional degrees	
None; the highest degree is DVM (or VMD)	6 (86)
Board certification	1 (14)
Others	0 (0)
Years of experience in clinical practice^a^	
<15 years	3 (43)
>15 years	4 (57)
Region of Practice	
Northeast	0 (0)
North-Central	1 (14)
Central	6 (86)
Southern	0 (0)

a Indicates a categorical variable that has been recategorized into two/three categories.

#### 3.2.2. Disease knowledge and risk perception of swine veterinarians

All the responders considered biosecurity practices “Very Important/Important” to prevent and control foreign and infectious animal diseases (Table 5). Eighty-six percent of the swine veterinarians were familiar with the current guidelines and practices for preventing and controlling foreign animal diseases. Fifty-seven percent of responders considered the occurrence of a foreign animal disease outbreak in the US mainland in the next 3 years as “very likely/likely” (Table 5). Two of seven veterinarians considered the likelihood of veterinarians transmitting an infectious disease from one animal to another as “unlikely/very unlikely” (Table 5). All veterinarians thought it was practical to follow effective biosecurity measures while handling animals (Table 5).

**Table 5:** Perception of swine veterinarians on foreign animal disease risk and biosecurity.

Perception questions^a^	*n* (%)
How important do you consider biosecurity practices for the prevention and control of foreign animal diseases (FADs)?	
Very Important/Important	7 (100)
Neutral	0 (0)
Less Important/Not at all Important	0 (0)
How important do you consider biosecurity practices for the prevention and control of infectious diseases?	
Very Important/Important	7 (100)
Neutral	0 (0)
Less Important/Not at all Important	0 (0)
How familiar are you with current guidelines and practices for the prevention and control of foreign animal disease (FAD) outbreaks?	
Extremely familiar/Moderately familiar	6 (86)
Somewhat familiar	0 (0)
Slightly familiar/Not at all familiar	1 (14)
How important is developing disease prevention and control plans to prepare for a foreign animal disease (FAD) outbreak?	
Very Important/Important	7 (100)
Neutral	0 (0)
Less Important/Not at all Important	0 (0)
How important do you consider disease surveillance and testing for the detection and prevention of infectious diseases?	
Very Important/Important	7 (100)
Neutral	0 (0)
Less Important/Not at all Important	0 (0)
What do you think is the likelihood of the occurrence of a foreign animal disease outbreak in the US mainland in the next 3 years?	
Very likely/Likely	4 (57)
Neutral	2 (29)
Unlikely/Very Unlikely	1 (14)
What is the likelihood of a veterinarian transmitting an infectious disease from one animal to another?	
Very likely/Likely	4 (57)
Neutral	1 (14)
Unlikely/Very Unlikely	2 (29)
How practical is it to follow effective biosecurity measures while handling animals in day-to-day practice?	
Very practical/Practical	7 (100)
Neutral	0 (0)
Less practical/Not at all practical	0 (0)

a Five-scale choices were collapsed and reported as three-scale.

Veterinarians were when asked about the most common biosecurity problem in Illinois livestock farms in an open-ended question. The assessment of the open-ended question revealed that veterinarians considered “people/workers” (*n* = 3) the most common biosecurity problem in Illinois livestock farms. Other problem areas reported by veterinarians include “Wean to finish biosecurity,” “Slaughter pig transportation,” “Insect and transportation contamination/migration,” and “Being brought in from another country.”

Five of seven veterinarians have participated in a foreign animal disease investigation. Five out of seven veterinarians have suspected or diagnosed a notifiable disease in the past 3 years in Illinois and reported it to the State Animal Health Official (SAHO) and one veterinarian to the United States Department of Agriculture - Animal and Plant Health Inspection Service - Area Veterinarian in Charge (USDA-APHIS-AVIC). Veterinarians were asked about their course of action if they suspect or diagnose the following diseases: ASF, PRRS, foot and mouth disease, equine influenza, rabies, salmonellosis, and leptospirosis. We also asked the veterinarians if they should report suspected or confirmed or both cases of foreign animal disease and the State/Nationally reportable disease to the state animal health official. The veterinarians received a score of 0/1 based on an incorrect/correct response. The swine veterinarians scored a median knowledge score of 5 (Range: 3–7).

#### 3.2.3. Biosecurity practices of swine veterinarians

All veterinarians practiced shower-in-shower-out before entering a swine farm if the facility was available (Table 6). Eighty-six percent of the responders discarded medical waste safely, and 71 % practiced moving from healthy to sick animals while examining swine on farms (Table 6).

**Table 6:** On-farm biosecurity practices of swine veterinarians.

Practices	Always	Sometimes	Rarely	Never
Wash hands between barns	3 (43)	2 (29)	1 (14)	1 (14)
Change disposables (like gloves, shoe covers, etc.) between barns	3 (43)	3 (43)	1 (14)	0 (0)
Change/disinfect boots between barns	5 (71)	1 (14)	1 (14)	0 (0)
Change coveralls between barns	3 (43)	3 (43)	1 (14)	0 (0)
Practice shower-in-shower-out, if available	7 (100)	0 (0)	0 (0)	0 (0)
Moving from healthy animal to sick animal for examination	5 (71)	1 (14)	1 (14)	0 (0)
Disinfect examination tools (Stethoscope, thermometer, etc.) between barns	3 (43)	3 (43)	0 (0)	1 (14)
Discard medical waste safely	6 (86)	1 (14)	0 (0)	0 (0)

Six of seven veterinarians visited multiple farms in a day. Six veterinarians washed their vehicles used during farm visits either between two farm visits (*n* = 3) when it was visibly dirty (*n* = 2), or at the end of each workday (*n* = 1). Five veterinarians disinfect the vehicle used during farm visits, either between two farm visits (*n* = 3) or at the end of each workday (*n* = 2). Four of seven veterinarians carry medical waste disposal cans in the vehicle used during farm visits.

#### 3.2.4. Biosecurity advice of swine veterinarians

Six of seven veterinarians have provided farm biosecurity assessments for their clients in the past 3 years, with a mean of 68.83 assessments per veterinarian.

All seven veterinarians indicated their clients were “extremely to moderately knowledgeable” about biosecurity (Table 7). They rated the general biosecurity practices followed at their clients’ swine farms as either “excellent/very good” (*n* = 6) or “good” (*n* = 1). Five veterinarians indicated that their clients “always” seek biosecurity-related advice from them (Table 7). All five exclusively/predominantly swine veterinarians reported providing biosecurity-related advice to their clients “during every consultation.” However, veterinarians in mixed animal practice provided biosecurity-related advice to their clients “only when they ask” or “during a disease outbreak on their own or nearby farm.” Four veterinarians reported being asked by producers to follow biosecurity protocols (e.g., changing coveralls, shower-in-shower-out, etc.) during their farm visits (Table 7).

**Table 7:** Responses of swine veterinarians to client-related questions.

Client-related questions	*n* (%)
How would you describe the overall knowledge of your swine producer clients towards biosecurity?^a^	
Extremely/Moderately knowledgeable	7 (100)
Somewhat knowledgeable	0 (0)
Slightly/Not at all knowledgeable	0 (0)
How would you describe the general biosecurity practices followed at the swine farms of your clients?^a^	
Excellent/Very Good	6 (86)
Good	1 (14)
Fair/Poor	0 (0)
Do your swine producer clients ask you for biosecurity-related advice?^a^	
Always/Often	5 (72)
Sometimes	2 (28)
Rarely/Never	0 (0)
When do you provide biosecurity advice to your clients?	
During every consultation	5 (72)
Only when they ask	1 (14)
During a disease outbreak on their own/nearby farm	1 (14)
Never	0 (0)
Do swine producers ask you to follow biosecurity protocols (e.g., changing of coveralls, shower-in-shower-out, etc.) during your farm visits?^a^	
Always/Often	5 (72)
Sometimes	1 (14)
Rarely/Never	1 (14)

a Five-scale choices were collapsed and reported as three-scale.

#### 3.2.5. Disease occurrences reported by swine veterinarians

Swine veterinarians reported Swine influenza (*n* = 5), PRRS (*n* = 4), Mycoplasmosis (*n* = 5), Colibacillosis (*n* = 5), and rotavirus (*n* = 4) as the most indicated disease occurrences at their client farms in the year 2020. The occurrences of PED and Seneca Valley Virus were either never or rare at their clients’ swine farms in the year 2020.

### 3.3. Swine biosecurity educational website

#### 3.3.1. Google analytics of the swine biosecurity website

Between July 5th, 2022, and November 5th, 2022, we had 485 website accesses (473 new and 12 returning users). The total event count (a measure of distinct user interaction on a website, e.g., loading a page, clicking a link) during this period was 5,500, and an average engagement time was 1 min and 46 s.

**Figure 3:**
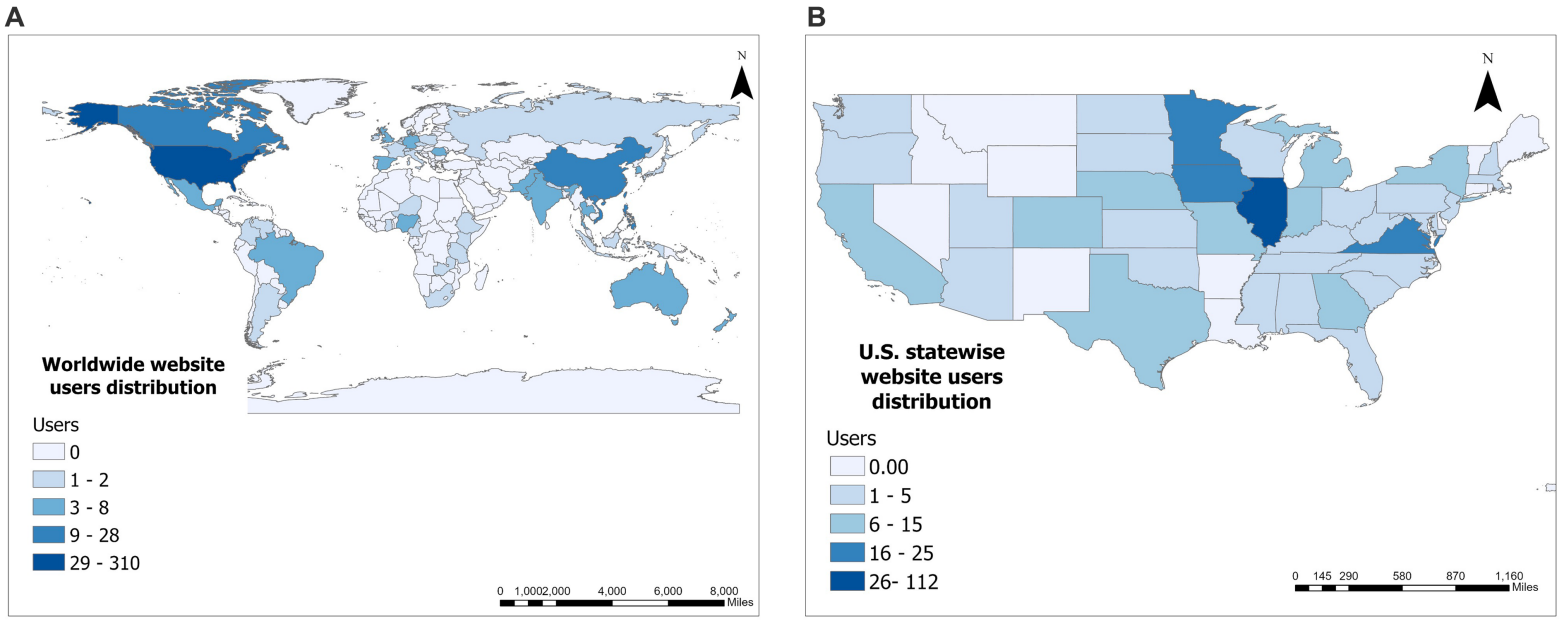
Website user distribution worldwide **(A)** and United States **(B)**.

Globally, most website users come from the US, Canada, and China (Figure 3A). Most US users accessing the website were from Illinois, Iowa, Minnesota, and North Carolina (Figure 3B).

**Figure 4:**
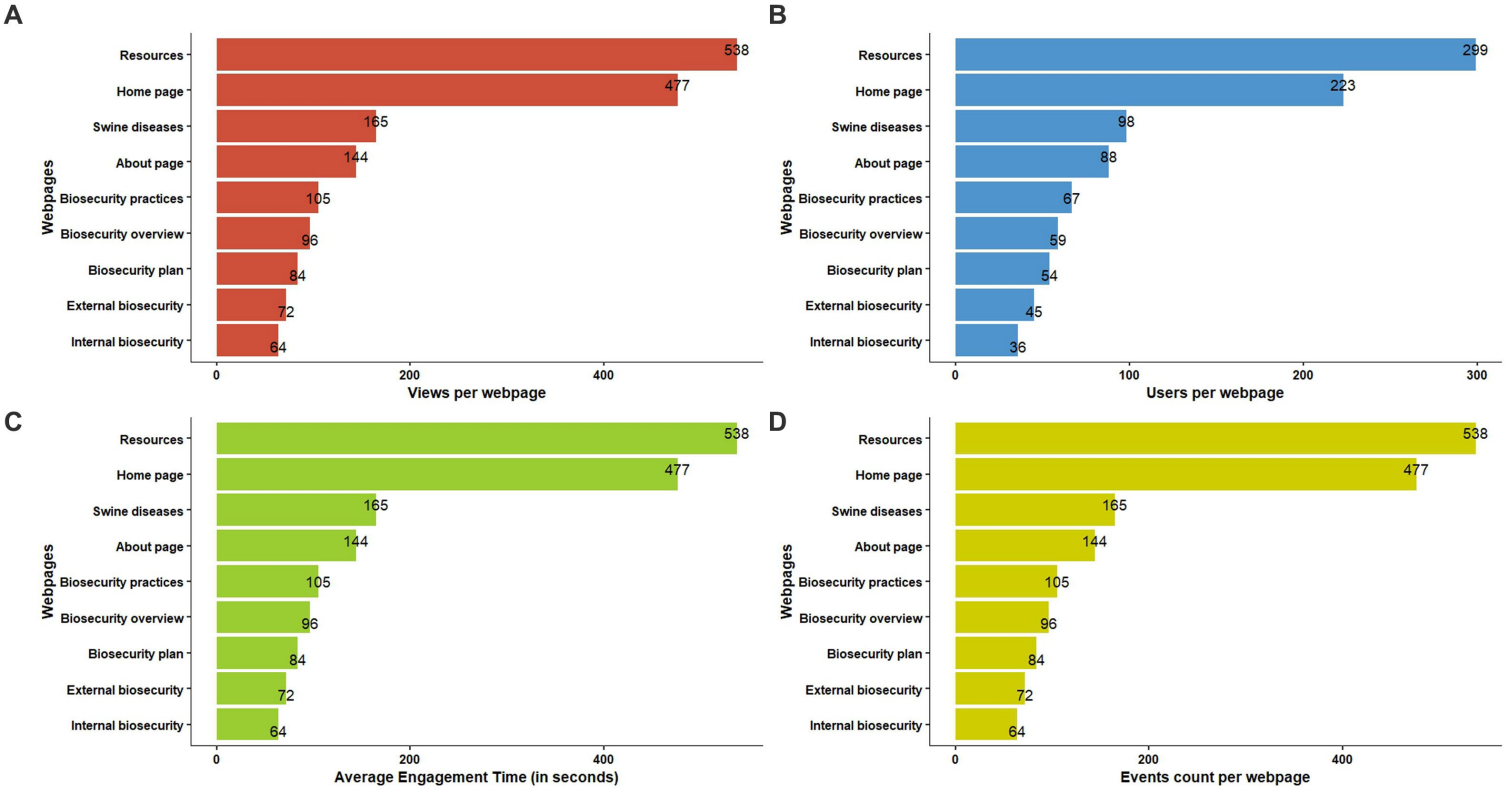
Google Analytics result for page-wise **(A)** views, **(B)** users, **(C)** average engagement time, and **(D)** event count.

**Figure 5:**
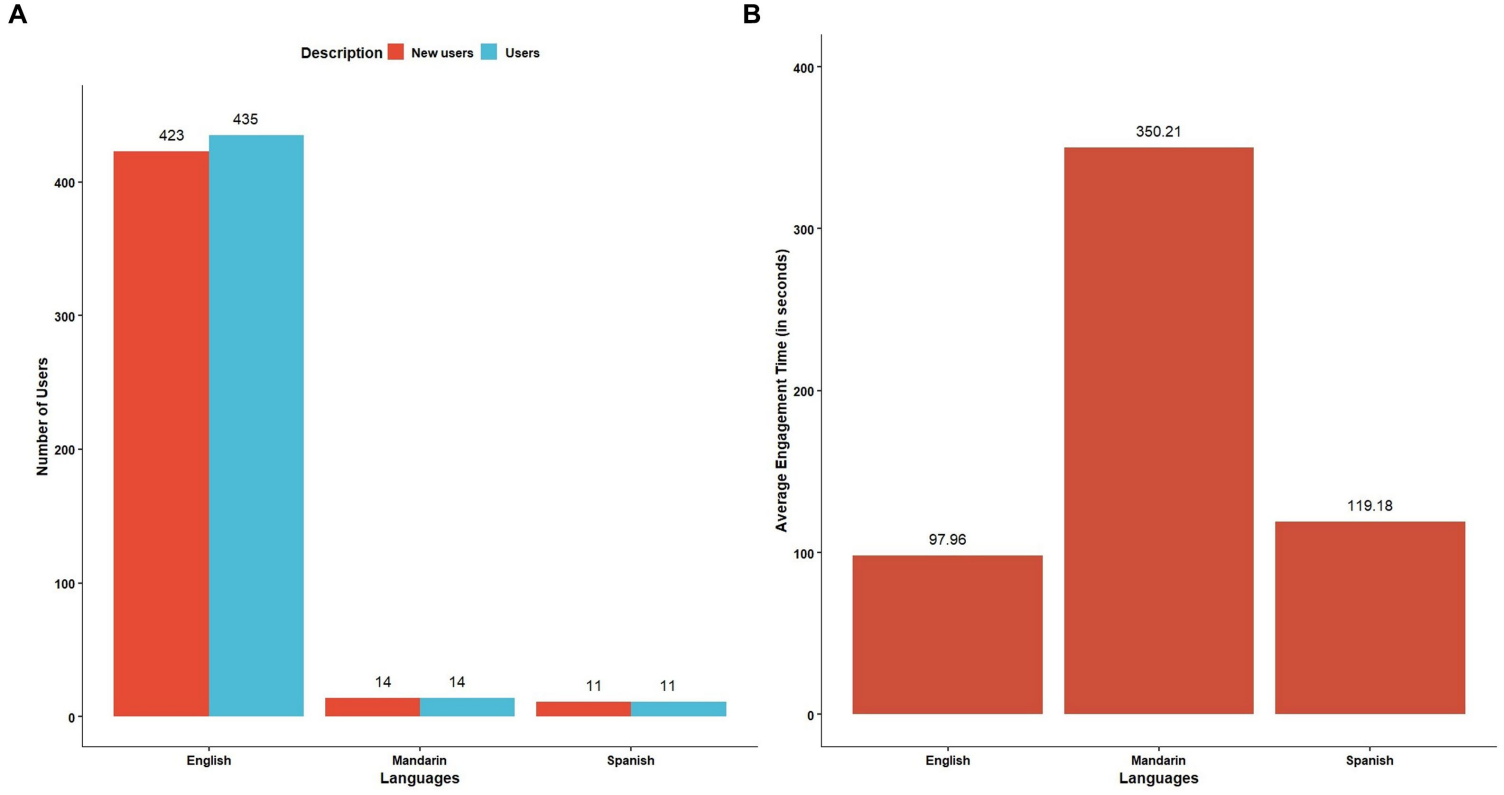
Google Analytics of the biosecurity website for language of users **(A)** user distribution **(B)** average engagement time.

**Figure 6:**
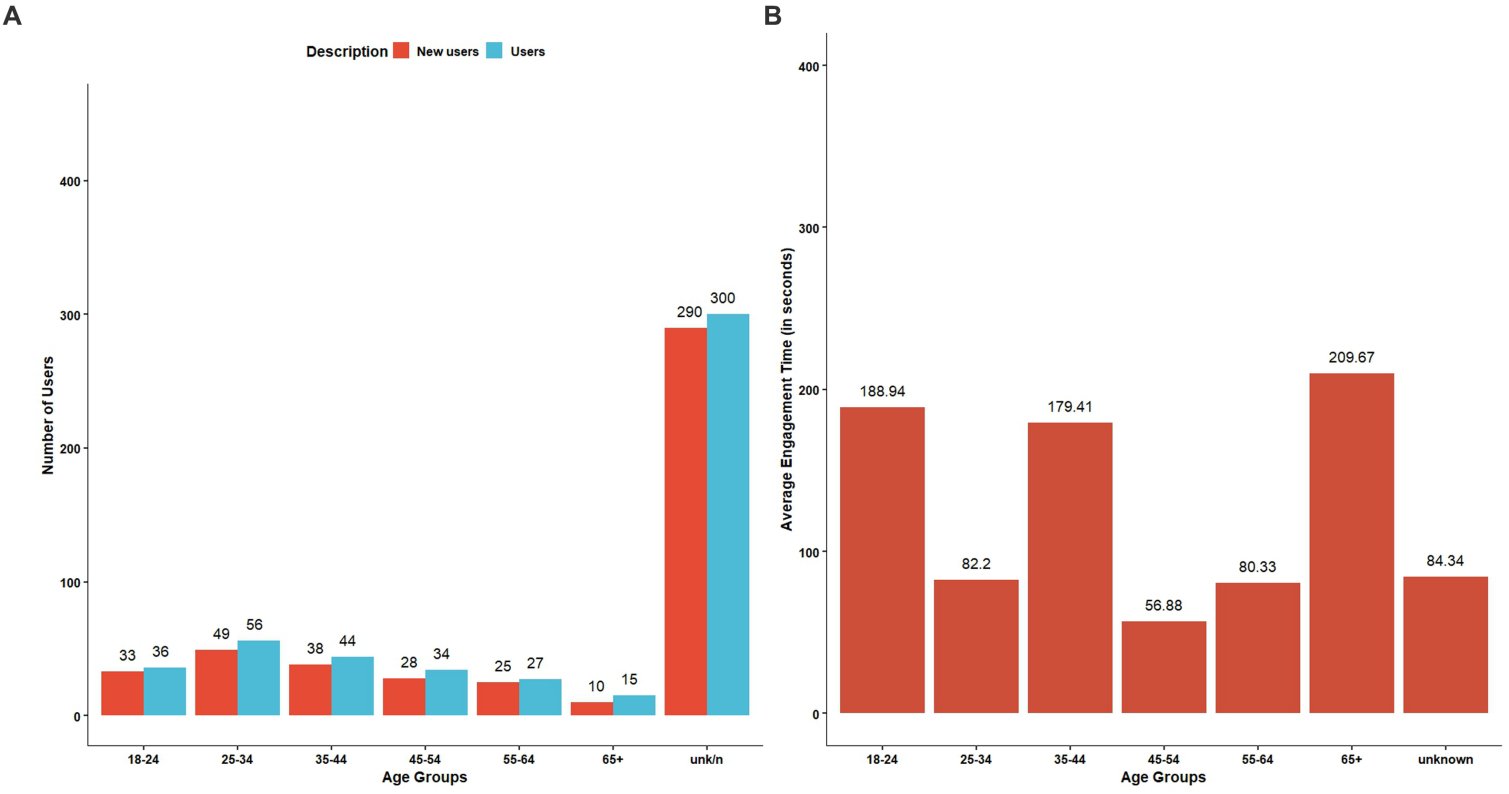
Google Analytics of the biosecurity website for user age groups **(A)** user distribution **(B)** average engagement time.

The webpage with the highest hits for views (Figure 4A), user count (Figure 4B), and event count (Figure 4D) was the resources page, whereas the highest average engagement time was for the swine diseases page (Figure 4C). English speakers, followed by Mandarin and Spanish speakers, formed the primary user base (Figure 5A). The highest average engagement time was for Mandarin speakers, followed by Spanish and English speakers (Figure 5B). The age distribution of the users who enabled the tracking of their age on their device while accessing our webpage was approximately uniform across all age groups; however, 300 users and 290 new users constituted the unknown age group (Figure 6A). Average engagement time was highest for the >65 years age group (24%), followed by 18–24 years (21%) and 35–44 years (20%) (Figure 6B).

Sixty-six percent of persons who accessed the website used their desktop, while 33% used mobile. Forty-six percent of the users accessed the website through direct links, 35% accessed the website through referrals, and 10% did an organic search to access the website.

**Figure 7:**
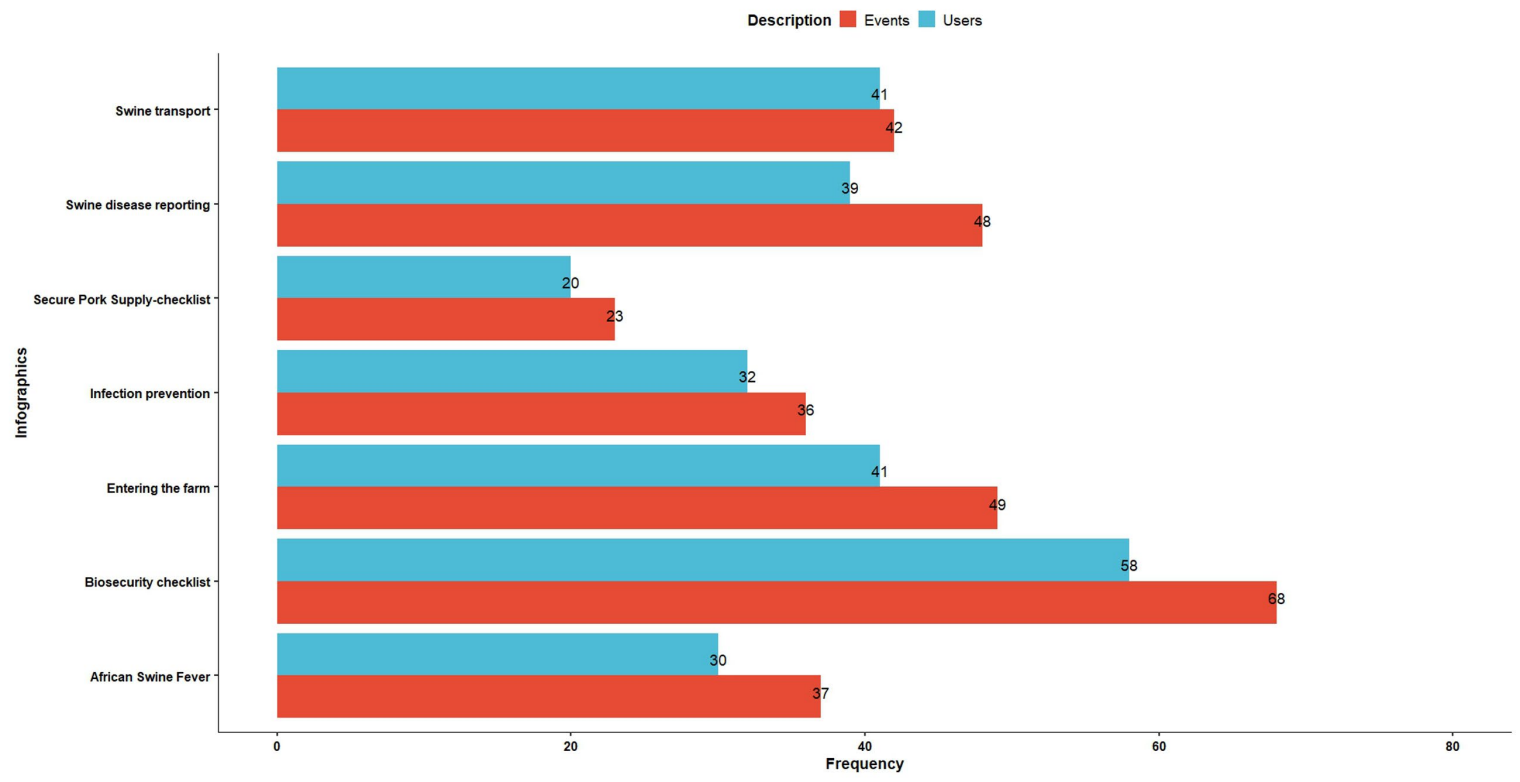
The frequency of total users and event counts (clicks/downloads) for each infographic on the resources page of the swine biosecurity website.

The biosecurity checklist infographic was viewed the most, with 58 users and 68 event counts (downloads), followed by entering the farm infographic and swine disease reporting infographic (Figure 7).

#### 3.3.2. Modules of the swine biosecurity website

The biosecurity overview module had the maximum responses provided by users (*n* = 26), followed by the swine diseases module (*n* = 12), external biosecurity (*n* = 7), Secure Pork Supply module (*n* = 7), and internal biosecurity module (*n* = 6). The general biosecurity practices module quiz had no response in the 4 months of the study period. The “Test your knowledge” quiz questions for each module with the descriptions of total responses and correct and incorrect responses for each question can be found in Supplementary File 5.

We calculated the mean score for each module. The highest mean score of 4.83 was provided for the external biosecurity module, and the lowest mean score was 2.67 for the Secure Pork Supply plan module. The mean scores for the remaining modules were: Swine diseases (3.67), Biosecurity overview (3.62), and Internal biosecurity (3.83).

## 4. Discussion

This study evaluated Illinois swine producers’ and veterinarians’ biosecurity knowledge, perception, and practices through two independent cross-sectional online surveys. Based on the survey results and our team’s expertise in biosecurity, we created an educational website to inform and improve biosecurity knowledge and practices among swine producers and veterinarians. In addition, outreach efforts were tracked via Google Analytics to monitor website traffic, user demographics, and engagement time to enable us to monitor the website’s effectiveness and upgrade it if needed.

### 4.1. Survey of swine producers

Thirteen responders completed the swine producers’ survey, managing 82 farms across nine counties in Illinois and raising over 85,000 pigs. Understanding swine producers’ attitudes toward biosecurity and their foreign animal disease risk perception will help swine stakeholders to tailor the policies and programs to encourage sustainable adoption of disease prevention and control practices by the swine producers on their farms (47). Results of the perception-based questions in our survey revealed that more than half of swine producers did not perceive the risk of occurrence of a foreign animal disease outbreak in the US in the next 5 years. However, a qualitative assessment study from the US indicated that the likelihood of ASF entry into the US is high, mainly through legal or illegal imports (including bioterrorism) of swine products and by-products, animal feed, or genetic material (43). In the survey responses, 5 swine producers owning 14 swine farms believe the impact of foreign animal disease outbreak would last anywhere between 3 months to 12 months. Conversely, a disease modeling and forecasting study suggested that the impact of a foreign animal disease introduction in the US could last two to over 10 years, depending on the response efforts implemented to control its spread (44). These results suggest that the swine producers under-perceived the risk and impact of foreign animal disease outbreaks in the US. Given that perception is a core component in determining human behavior (19), the perception and attitude observed in the study results may negatively affect adopting biosecurity practices by swine producers.

Our study found that most swine farms followed the majority of the required biosecurity practices. Nevertheless, these results should not be generalized because a single producer (contractor/integrator) who owned a considerable proportion of farms (60 farms) and had a Secure Pork Supply plan implemented for his farms impacted the overall finding of this section. By removing the large integrator’s response, all the other producers’ biosecurity practices, such as isolating new incoming animals, disease monitoring, wild animal exclusion, bird control, requiring vehicles to be washed and disinfected before entry into the farm, showering before entering and exiting the farm, and having a separate entrance for waste-collecting vehicles were among the least commonly adopted biosecurity practices. Some of these biosecurity practices are expensive (e.g., setting up a showering facility) and are labor-intensive (e.g., disease monitoring), which are often limiting the implementation of these practices, especially for small-scale producers (35, 45). Before interpreting the results of the swine producer survey, some limitations need to be noted, particularly the low response rate, limiting the generalizability of our study results. The IPPA represents around 1,600 swine producers in Illinois, and as per USDA’s 2017 agriculture census data, there were 2,153 swine producers in Illinois. This, and the low response rate, limited the representativeness of our survey results. Future studies should incentivize producers who complete the survey (48) or use multiple survey modes or send multiple reminders at appropriate intervals to get a higher number of responses from swine producers (49).

### 4.2. Survey of swine veterinarians

Our study results revealed a positive perception of Illinois swine veterinarians towards biosecurity and its importance in infectious and foreign animal disease prevention and control. Unlike swine producers, swine veterinarians in Illinois had a higher foreign animal disease risk perception and considered the likelihood of a foreign animal disease outbreak in the US as highly likely. This is an encouraging finding as perceived risk is the driver of an individual’s risk-taking behavior (35, 50), suggesting that lower perceived risk might result in a lack of diligence in adopting and implementing biosecurity measures.

In our study, veterinarians considered people/workers the most common threat to farm biosecurity, which concurs with previous studies (3, 36). On the other hand, 29% of Illinois swine veterinarians thought it was “unlikely/very unlikely” for a veterinarian to transmit a disease from one animal to another, contrary to what previous studies suggested (24, 37, 51, 52). These findings are concerning as previous studies have shown that veterinarians’ perceptions can substantially influence their adoption of effective disease prevention practices (41). Moreover, despite awareness and having biosecurity training, many Illinois swine veterinarians did not always follow simple biosecurity practices like washing hands, changing disposable clothes and boots, and disinfecting examination tools between barns. The probable explanation for not following these practices could be a low perceived risk from these measures or their application being impractical in daily practice.

Previous studies have highlighted the role of swine veterinarians as a primary translator of information on biosecurity and related practices for swine producers (24, 42, 53, 54). In our study, swine veterinarians reported providing biosecurity advice to their clients during every consultation.

The survey results represent the opinion of an estimated 28% of the total swine veterinarians in Illinois. However, there might be non-response bias wherein the respondents’ opinions and practices might systematically differ from the non-respondents (55). Therefore, these results should be interpreted with caution.

### 4.3. Swine biosecurity educational website

We used the health-belief model as the conceptual framework for website development and chose a web-based educational tool as it serves as a medium to reach a large segment of swine producers and veterinarians. We developed a web-based educational tool and evaluated the outreach efforts using Google Analytics and content comprehension using self-assessing quizzes. Previous studies have used Google Analytics effectively as a web analytics tool for educational, commercial, and university websites (29–31). Seemingly, quizzes have become a popular way of assessing content comprehension and user interaction (56–58). For our study, Google Analytics helped examine the network behavioral patterns of the website users, and we deem our outreach effort successful as 473 new users accessed the site in the initial 4-month period of its launch. We expected a high access number of site visitors from Illinois due to our local outreach efforts, but we also received a high access number from Iowa, Minnesota, and North Carolina, the leading swine-producing states of the US, which is an encouraging finding. The average engagement time for the website was towards the lower end, suggesting future efforts should be made to improve the website content and enhance user experience to appeal to a larger audience. On a positive note, the website had users from around the world. However, a good proportion of our users were Mandarin and Spanish speakers. These findings were expected as China is a leading pork producer worldwide (59), which recently experienced a devastating ASF outbreak (60) that might increase pork producers’ awareness of infectious swine diseases and eagerness to learn more about effective disease prevention methods. The high proportion of Spanish-speaking visitors on the website can be explained by the data suggesting that over three-quarters of agricultural workers worldwide are Spanish speakers (61, 62). These results give us the future direction of expanding the website content in other languages.

The swine diseases page showed the maximum user engagement time, and the resources page had the top hit for views, user count, and event count. The resource page consisted of infographics related to reporting swine diseases, biosecurity protocols for entering a swine farm, transportation biosecurity, a biosecurity checklist for a swine farm, a description of the Secure Pork Supply plan, an information fact sheet on ASF, along with signs in downloadable format. Infographics are information snapshots consisting of the pictorial depiction of data in a concise manner (63), and they attract the user’s attention and enable better retention of information (64–66). Building over this concept, we designed these infographics to provide relevant information on swine diseases and effective biosecurity protocols concisely that the producers and veterinarians could use in their work settings to educate the farm employees. Infographics have proved to be a popular, effective, and feasible mode of information dissemination, and our study results on the resource page agree with the findings of previous studies on infographics’ effectiveness (65).

The study results of Google Analytics should be interpreted with caution for several reasons. The Google Analytics data has limitations because it does not record all website activities and only includes demographic information of users who made it public on their Google accounts. The reports show approximates of actual data and cannot identify the occupation of visitors. The access granted by Google Analytics and the anonymity of users limits the granularity of the data collected and is subjected to missing information. Moreover, we cannot ascertain but expect that most website visits came from swine stakeholders, veterinarians, and people interested in swine disease prevention and control as we shared our website on platforms with our targeted user base such as swine health and production-related websites, academic and professional social media platforms, the official website of the College of Veterinary Medicine, University of Illinois Urbana Champaign, and the ISVMA newsletter. Nevertheless, Google Analytics data gives us a good approximation of what is working on the website and what needs more attention, highlighting the areas that require re-evaluation and upgrade. Our study results suggest that we should allocate effort into designing more infographics and adding additional content to the swine diseases modules as these web pages attracted the most attention among users.

To facilitate learning and evaluate users’ understanding of the module content, we embedded short quizzes with automated marking at the end of each of the six modules. Periodic quizzes have enhanced learning experiences by reiterating important concepts, promoting active learning, and increasing learners’ engagement (56, 57, 67). In addition, automated feedback has been described as improving comprehension and providing a quick snapshot of users’ understanding of the subject (57). However, compared to the number of website users, the response rate for the quizzes embedded in the swine biosecurity modules was low. The plausible explanation for this low response rate for the quizzes could be the time constraints of users or a non-aligning interest in swine biosecurity. However, the users who did take an attempt on the quizzes performed well, indicating either prior background in swine biosecurity or an adequate understanding of the concepts provided through the modules after reading them.

## 5. Conclusion

Our study developed a novel methodology that utilized a stepwise method of assessing-developing-evaluating-improving. It assessed the knowledge and perception of swine producers and veterinarians on swine disease risk and biosecurity practices, developed an educational website to address the gaps identified through the surveys, and evaluated the outreach and efficacy of the website, with a final goal to improve foreign animal disease risk awareness and biosecurity practices of swine producers and veterinarians in Illinois, in the US, and worldwide. Understanding swine producers’ and veterinarians’ perceptions of biosecurity and its importance in disease control and prevention can help policymakers develop targeted programs and aid researchers and extension staff in tailoring their swine biosecurity educational efforts.

## Data availability statement

The original contributions presented in the study are included in the article/Supplementary material, further inquiries can be directed to the corresponding author.

## Ethics statement

The studies involving human participants were reviewed and approved by The Institutional Review Board (IRB) of the University of Illinois at Urbana Champaign (IRB #21974). The participants provided their written informed consent to participate in this study.

## Author contributions

IA and CV: study design and writing—original draft. IA: data analysis and visualization. CV and CB: resources. IA, CV, and CB: writing—review and editing. CV: supervision and project administration. All authors contributed to the article and approved the submitted version.

## Funding

This research was funded by Farm Bill funding through USDA Animal and Plant Health Inspection Service (APHIS) Veterinary Services (VS)’ National Animal Disease Preparedness and Response Program (NADPRP) 2020 (Award number: AP21VSSP0000C037).

## Conflict of interest

The authors declare that the research was conducted in the absence of any commercial or financial relationships that could be construed as a potential conflict of interest.

## Publisher’s note

All claims expressed in this article are solely those of the authors and do not necessarily represent those of their affiliated organizations, or those of the publisher, the editors and the reviewers. Any product that may be evaluated in this article, or claim that may be made by its manufacturer, is not guaranteed or endorsed by the publisher.
