# Supplementary material for: Assessing and improving on-farm biosecurity knowledge and practices among swine producers and veterinarians through online surveys and an educational website in Illinois, United States
Source: Front Vet Sci. 2023 Jun 9;10:1167056. doi: 10.3389/fvets.2023.1167056 (PMC10289165; doi:10.3389/fvets.2023.1167056)
Supplement: Supplementary file 1 [file Data_Sheet_1.PDF]

# Swine_Biosecurity_Survey

## Index:

**Single farm questions.**

**Multi farm questions**

**Common questions irrespective of single or multi-farm setup.**
**There are few questions which will display based on the option selected in the previous question. These questions are highlighted in for every block expect for the Multifarm block as all questions in that block have some sort of display logic.**

**All skip questions are numbered so they are easy to follow.**

**Questions with * are forced response questions which cannot be skipped.**

**All blocks starting and ending is highlighted in yellow with bolded text.**

## Survey Flow:

Block: Consent (5 Questions)
Standard: Demographic & Farm Information (11 Questions)
Standard: Disease Risk Perceptions and Knowledge (11 Questions)
Standard: Farm Characteristics (18 Questions)

Branch: New Branch
If
If Do you operate multiple hog farms? Yes Is Selected

Block: mf (22 Questions)
Block: Practices related to the movement of people at your farm (38 Questions)
Block: Practices related to animal movements onto your farm (14 Questions)
Block: Animal Health Management (7 Questions)
Block: Previous Disease Outbreaks (4 Questions)
Block: Multi-farm (18 Questions)
Block: Feedback (5 Questions)

EndSurvey:

Standard: Practices related to the movement of people at your farm (38 Questions)
Standard: Practices related to animal movements onto your farm (14 Questions)
Standard: Animal Health Management (7 Questions)
Standard: Previous Disease Outbreaks (4 Questions)
Standard: Feedback (5 Questions)

Page Break

**Start of Block: Consent**

## Consent Form

### Assessing Biosecurity Knowledge and Practices Among Illinois Swine Producers

Effective biosecurity practices on hog farms are vital for safeguarding our swine population. Disease outbreaks such as African Swine Fever (ASF) could potentially cost the hog industry billions of US dollars. Hog producers, like yourself, as well as veterinarians, are an important part of protecting our hog industry from such devastation. Understanding your prevention practices will help in developing educational programs that will enhance your biosecurity plans that will prove useful especially during a foreign animal disease (FAD) outbreak.

You are being asked to participate in a voluntary research study conducted by Drs. Varga, Miller, and Agrawal, veterinarians at the **College of Veterinary Medicine**. They are working in collaboration with **Illinois Pork Producers Association(IPPA)** to assess current biosecurity and farm management practices used by Illinois pork producers.
Study participation requires you to complete a web-based (online) questionnaire on practices related to the movement of people and animals onto your farm, your animal health management practices, and disease risk perceptions. The survey **will take about 20 minute**s to complete.

If you are unable to access the web-based survey, another alternative is to call **Dr. Csaba Varga (Principal Investigator)** at 217-480-6983 or contact him via email at cvarga@illinois.edu and complete the survey via telephone.
The study is sponsored by the **United States Department of Agriculture (USDA)**, Animal and Plant Health Inspection Service. This is for work that supports our National Animal Disease Preparedness and Response Program (NADPRP).

We will maintain the confidentiality of your information to the extent permitted and required by laws and university policies. Your name or personal identifiers will not be published or presented. Your de-identified information could be used for future research without additional informed consent.

No monetary incentives will be given for participation in this study. Your participation is voluntary, you may refuse to participate or discontinue participation at any time. This will involve no penalty or loss of benefits to which you are otherwise entitled; neither will it affect your current or future dealings with the University of Illinois at Urbana-Champaign or Illinois Pork Producers Association (IPPA).

If you have questions about this project, please contact Dr. Csaba Varga at 217-480-6983 or cvarga@illinois.edu. If you have any questions about your rights as a participant in this study or

any concerns or complaints, please contact the **University of Illinois at Urbana-Champaign Office for the Protection of Research Subjects at 217-333-2670 or via email at irb@illinois.edu.**

Please print this consent form if you would like to retain a copy for your records. I have read and understood the above consent form. I certify that I am 18 years old or older. By clicking the **"Yes, I consent"** option to enter the survey, I indicate my willingness to voluntarily take part in this study.

---

**Do you consent to participate in this study?**

- Yes, I consent
- No, I do not wish to be a part of this study

*Skip To: If Do you consent to participate in this study? = No, I do not wish to be a part of this study*

*Skip To: End of Block If Do you consent to participate in this study? = Yes, I consent*

---

**Please specify the reason for your non-participation**

- Not interested
- Not beneficial to me
- Not relevant to me
- Do not have time
- Others ________________________________________________

*Skip To: End of Survey If Please specify the reason for your non-participation = Not interested*

*Skip To: End of Survey If Please specify the reason for your non-participation = Not beneficial to me*

*Skip To: End of Survey If Please specify the reason for your non-participation = Others*

*Skip To: End of Survey If Condition: Others Is Not Empty. Skip To: End of Survey.*

*Skip To: End of Survey If Please specify the reason for your non-participation = Do not have time*

*Skip To: End of Survey If Please specify the reason for your non-participation = Not relevant to me*

**End of Block: Consent**

---

**Start of Block: Demographic & Farm Information**

## Let us talk briefly about you and your farm operations.

**Q1.(a) How would you describe your farm business structure under which you are presently supplying hogs?**

- **Independent producer** (Hog producer that owns and operates his/her farm)
- **Contractor or integrator** (Corporate pork producer company who provides pigs or breeding stock, feed, and other services to contract hog producers to raise hogs till a desired stage of production)
- **Contract hog producer** (Hog producers who raise hogs for a contractor/integrator)

Other, Please Specify ________________________

**Q1.(b) How would you describe your role on the hog farm(s)? (Check all that apply)**

- Farm owner
- Farm manager
- Veterinarian
- Others, Please Specify ____________________________

**Q2. What is your age? (in years)**

___________________________

**Q3. What is your sex?**

- Male
- Female
- Prefer not to answer

**Q4. What is the highest level of education you have completed?**

- Did not complete high school
- High school or equivalent
- Some college or Associates degree
- 4-year college or Bachelor's degree
- Graduate degree (MSc, Ph.D., DVM, etc.)

**Q5. How long have you been in hog production (years of experience)?**

- <5 years
- 5-10 years
- 11-25 years
- 26-50 years
- >50 years

**Q6. How beneficial do you think your biosecurity measures are at preventing disease introduction into your hog farm? (**By **biosecurity** we mean practices and procedures that are aimed at reducing the chances of disease introduction and spread within and between farms.)

- Extremely beneficial
- Beneficial
- Moderately Beneficial
- Somewhat Beneficial
- Not beneficial at all

**Q7. Where do you currently spend most of your money out of your farm health management funds?**

- Prevention of diseases
- Treatment of diseases

**Q8. If given a choice, which do you generally think is more cost-effective?**

- Preventing diseases from infecting your animals
- Treating your animals when they have a disease

**Q9. Did you spend any money on disease prevention and control in the previous year?**

- I do not spend on disease prevention
- I spend approximately $ amount per pig

  ________________________________________________

*Display This Question:*

*If Did you spend any money on disease prevention and control every year? = I spend approximately $ amount*

*And And Did you spend any money on disease prevention and control in last 12 months?<o:p style="-webkit-user-select: auto;"></o:p> I spend approximately $ amount  Is Not Empty*

**Q10. Was the amount you spent on disease prevention and control in 2020 about the same as most years?**

- Yes
- No, I have spent more per pig in 2020
- No, I have spent less per pig in 2020

**End of Block: Demographic & Farm Information**

---

**Start of Block: Disease Risk Perceptions and Knowledge**

## Now we will be proceeding towards the disease risk you are aware of at your hog farm.

**Q11. Are you familiar with foreign animal diseases (FAD) such as African swine fever (ASF) / Foot and mouth disease (FMD)?**

- I have full knowledge about these diseases
- I have a decent knowledge of these diseases
- I have a superficial knowledge of these diseases
- I have just heard about these diseases
- I have never heard about these diseases

**Q12. If you suspect an emergency foreign animal disease (FAD) in your herd, who are you most likely to contact?**

- I would not call anybody
- Private veterinarian
- IL State veterinarian
- Extension Agent
- Neighbor
- Other, Please Specify ______________________________

**Q13. Do you have a System for Award Management (SAM) registration for your hog farm(s)?** (**System for Award Management (SAM)** is a U.S. Federal Government-owned and operated website, used for free to register to do business with the U.S. government.)

- Yes
- No

**Q14. Do you have a Data Universal Numbering System (DUNS) number for your hog farm(s)?** *(Dun & Bradstreet (D&B) provides a **D-U-N-S Number**, a unique nine-digit identification number, for each physical location of your business. The federal government uses the DUNS number to track how federal money is allocated.)*

- Yes
- No

**Q15. If a foreign animal disease (FAD) outbreak occurred on your farm, what do you think the government indemnity payment approach would be?**

- No indemnity payments will be available
- Indemnity payments will be available for all farmers regardless of their disease prevention and control efforts
- Indemnity payment will be available only for farmers who demonstrate and document disease prevention and control efforts
- Indemnity payment will be available only for farmers who are registered with SAM and have a DUNS number.

**Q16. If a foreign animal disease (FAD) outbreak occurred on your farm, in your opinion, how long would the negative impacts on your operation persist?**

- less than 1 month
- 1-2 months
- 3-6 months
- 6-12 months
- more than 12 months

**Q17. How important do you consider developing an enhanced disease prevention and control plan (e.g. Secure Pork Supply Plan) that can be used during a foreign animal disease (FAD) outbreak for your hog farm?**

- Extremely important
- Very important
- Moderately important
- Slightly important
- Not at all important

**Q18. How important do you consider routine testing for the detection and prevention of specific diseases on your hog farm(s)?**

- Very important
- Important
- Moderately Important
- Slightly Important
- Not important at all

**Q19. What do you think is the likelihood of occurrence of foreign animal disease (FAD) outbreak in the next five years in the US swine industry?**

- Very likely
- Somewhat likely
- Not likely at all
- Don't know

**Q20. How would you rate your hog operation's disease prevention and control measures?**

- Very Poor
- Poor
- Acceptable
- Good
- Very Good

## End of Block: Disease Risk Perceptions and Knowledge

---

## Start of Block: Farm Characteristics

## Now let's talk a little more about your hog farm and its management practices.

---

**Q21. Do you operate multiple hog farms in Illinois?**

- Yes
- No

*Skip To: End of Block If Do you operate multiple hog farms? = Yes*

---

**Q22. In which Illinois county is your hog farm located?**

County

| | | | | | | |
|---|---|---|---|---|---|---|
| Adams | Coles | Fulton | Jo Daviess | Madison | Ogle | Scott |
| Alexander | Cook Crawford | Gallatin | Johnson | Marion Marshall | Peoria | Shelby |
| Bond | Cumberland | Greene | Kane | Mason Massac | Perry | St. Clair |
| Boone | De Witt | Grundy | Kankakee | McDonough | Piatt | Stark |
| Brown | DeKalb | Hamilton | Kendall | McHenry | Pike | Stephenson |
| Bureau | Douglas | Hancock | Knox | McLean | Pope | Tazewell |
| Calhoun | DuPage | Hardin | Lake | Menard | Pulaski | Union |
| Carroll | Edgar | Henderson | LaSalle | Mercer Monroe | Putnam | Vermilion |
| Cass | Edwards | Henry | Lawrence | Montgomery | Randolph | Wabash |
| Champaign | Effingham | Iroquois | Lee | Morgan | Richland | Warren |
| Christian | Fayette | Jackson | Livingston | Moultrie | Rock Island | Washington |
| Clark | Ford | Jasper | Logan | | Saline | Wayne |
| Clay | Franklin | Jefferson | Macon | | Sangamon | White |
| Clinton | | Jersey | Macoupin | | Schuyler | Whiteside |
| | | | | | | Will |
| | | | | | | Williamson |
| | | | | | | Winnebago |
| | | | | | | Woodford |

**Q23. Is your primary residence located at your hog farm?**

- Yes
- No

*Display This Question:*

*If Is your primary residence located at your hog farm? = Yes*

**Q24. Does your primary residence share the driveway or entrance with your hog farm?**

- Yes
- No

**Q25. How would you describe your hog farm?**

- **Farrow to finish farm (**Involves all stages of production, raising pigs from breeding through finishing to market weights)
- **Farrow to wean farm** (Involves raising pigs from breeding through marketing 10- to 15-pound weaned pigs for nursery-grow-finish farms)
- **Farrow to nursery/feeder farm** (Involves raising pigs from breeding through marketing 40- to 60-pound feeder pigs for finishing farms)
- **Wean-to-finish farm** (Involves raising weaned pigs and finishing them to market weights)
- **Nursery farm** (involves raising weaned pigs through 40- to 60-pound feeder pigs)
- **Finishing farm** (Involves raising 40- to 60-pound feeder pigs and finish them to market weight)
- Other, Please Specify ________________________________________________

**Q26. Do you have a Premise Identification Number for your hog farm?** (***Premises Identification Number*** *(Premises ID or Prem ID or PIN) is the identification of a location where livestock are raised, housed, or pass through during commerce and is assigned by the Illinois Department of Agriculture which allows animal health officials to quickly and precisely identify where animals are located in the event of an animal health emergency.*)

- Yes
- No

**Q27. How many other livestock farms are situated within a 3-mile radius of your hog farm?**

- None
- 1 farm
- 2-3 farms
- 4-5 farms
- More than 5 farms

**Q28. How many pigs do you have on your hog farm as of today? (Specify their number)**

________________

**Q29. Do you keep any other type of livestock/poultry species on your hog farm?**

- Yes
- No

*Display This Question:*

*If Do you keep any other type of livestock/poultry species on your hog farm? = Yes*

**Q30. What type of livestock and/or poultry do you keep on your farm? (Check all that apply)**

- Cattle
- Sheep and/or Goat
- Poultry
- Other, Please Specify ________________

**Q31. Which type of pig housing is used at your hog farm?**

- Total confinement with mechanical ventilation
- Total confinement with natural ventilation
- Open building with outside access for pigs
- Other, Please Specify ________________

*Display This Question:*

*If Which type of pig housing is used at your hog farm? = Total confinement with mechanical ventilation*

**Q32. Do you have an air filtration unit installed in the barn(s) that house the pigs?**

- Yes
- No

**Q33. How many barn(s) do you have that house pigs at your hog farm? (Please specify their number)**

________________

**Q34. How many barn(s) are less than 10 years old that house pigs at your hog farm?**

- None
- 1 barn
- 2 barns
- 3-4 barns
- More than 4 barns

**Q35. Do you have a Secure Pork Supply Plan for your hog farm?** (A **Secure Pork Supply Plan** lists current biosecurity practices implemented at your hog operation and provides opportunities to voluntarily prepare before a foreign animal disease (FAD) outbreak.)

- Yes
- No

*Display This Question:*

*If Note: A Secure Pork Supply Plan lists current biosecurity practices implemented at your hog opera... = No*

**Q36. Have you had a biosecurity assessment done for your hog farm?** (**Biosecurity assessment** evaluates current practices and identifies how to reduce disease introductions and/or spread. Biosecurity assessments can be done by you, your veterinarian, another experienced swine producer, a biosecurity officer, or other appropriately knowledgeable people.)

- Yes
- No

*Display This Question:*

*If Note: Biosecurity assessment evaluates current practices and identifies how to reduce disease int... = Yes*

**Q37. When was the last biosecurity assessment done for your hog farm?**

- 1-6 months ago
- 7-12 months ago
- 13-24 months ago
- More than 24 months ago

## End of Block: Farm Characteristics

---

## Start of Block: Practices related to the movement of people at your farm

## Let’s dive into the movement of people onto your hog farm.

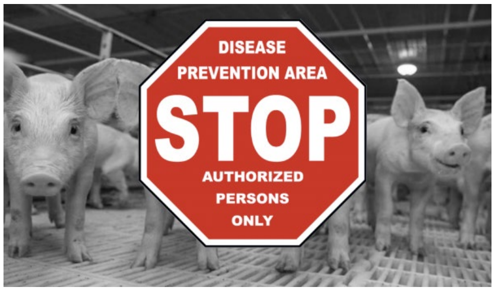


**Q58. Do you have signage (similar to the one shown above) at your hog farm entrance indicating "a biosecure area"?**

- Yes
- No

**Q59. Do you have gates or barriers to control entry at the main entrance of your hog farm?**

- Yes
- No

*Display This Question:*

*If Do you have gates or barriers to control entry at the main entrance of your hog farm? = Yes*

**Q60. Do you restrict entry to your hog farm using a closed gate, when the facility is not attended to?**

- Yes
- No

**Q61. Do you have a separate entrance for vehicles, which collect and transport farm waste (e.g., manure and/or deadstock) from your hog farm?**

- Yes
- No

**Q62. How many entry point(s) do you have to your hog farm?**

- 1
- 2
- More than 2

**Q63. Do you have a logbook that records every entry (e.g., identity, date, time, and purpose of visit) on your hog farm?**

- Yes
- No

**Q64. Do you allow vehicles to enter your hog farm and park within 10 feet of the barn(s) where pigs are housed?**

- Yes
- No

**Q65. Do you require vehicles (including trucks) to be washed and checked to be free of visible dirt before entering your hog farm?**

- Yes
- No

**Q66. Which of the following structures (if any) are present at your hog farm? (Check all that apply)**

- Designated parking area for trucks and visitors
- Loading and unloading area for pigs
- Delivery areas for feed
- Truck wash unit
- Cleaning and disinfection station
- Handwashing area
- Toilet
- Area for changing into farm-specific clothes and boots
- None of the above

**Q67. Do you have a well-defined perimeter buffer area (an outer control boundary, set up around the farm buildings to minimize potential pathogen introduction near animal housing) at your hog farm?**

- Yes
- No

*Skip To: Q69 If Do you have a well-defined perimeter buffer area (an outer control boundary, set up around the fa... = No*

**Q68. Which of the following facilities are located outside the perimeter buffer area? (Check all that apply)**

- A designated parking lot for non-farm/non-animal vehicles
- Loading and unloading areas
- Farm personnel/ owner's housing units
- Cleaning & Disinfection station
- Feed storage unit
- None of the above

**Q69. Do you have employees caring for your hogs at your hog farm?**

- Yes
- No

*Display This Question:*

*If Do you have employees caring for your hogs at your hog farm?  = No*

**Q70. Do you work at other hog farms during a given workday?**

- Yes
- No

*Display This Question:*

*If Do you have employees caring for your hogs at your hog farm?  = Yes*

**Q71. Do any of your employees work at other hog farms during a given workday?**

- Yes
- No

**Q72. Do people (yourself, employees, family members, or visitors) change into farm-specific coveralls and boots before entering your hog farm?**

- Yes
- No

*Display This Question:*

*If Do people (yourself, employees, family members, or visitors) change into farm-specific coveralls... = Yes*

**Q73. Do people (yourself, employees, family members or visitors) take a shower before entering your hog farm?**

- Yes
- No

Page Break

## Moving on, we will talk about the structures and facilities available at your hog farm.

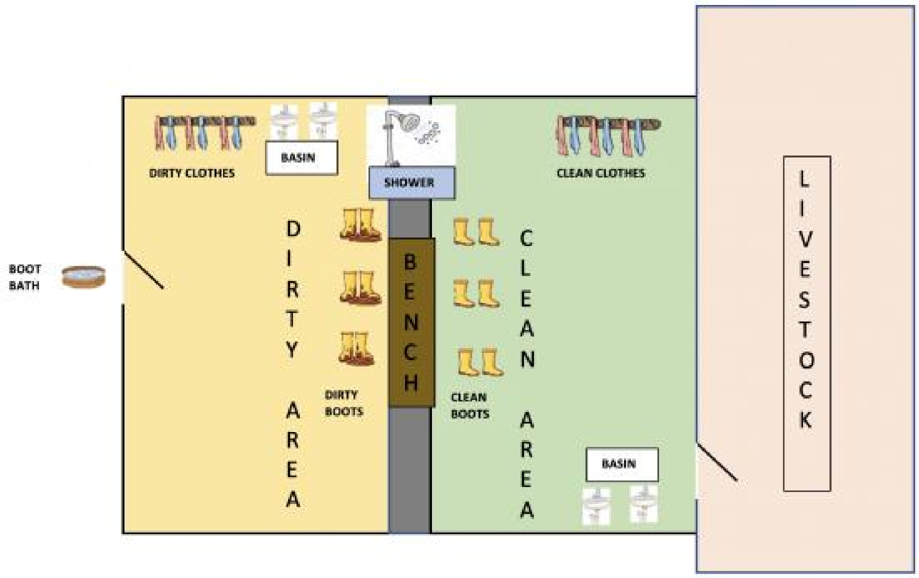


**Q74. Do you have a clearly defined line of separation (building walls separating the pigs from the outside) for each building that houses pigs?**

- Yes
- No

*Skip To: Q76 If Do you have a clearly defined line of separation (building walls separating the pigs from the out... = No*

**Q75. Do you always lock buildings where pigs are kept when no one is present to restrict the entry of unauthorized people into the barn(s)?**

- Yes
- No

**Q76. Do you have designated entry point(s) for people entering the barn where pigs are kept?**

- Yes
- No

**Q77. Do you have a logbook for records of who entered the barn where pigs are kept?**

- Yes
- No

**Q78. Do you require visitors and employees who enter the barn where pigs are housed to change into farm-specific clothing and boots?**

- Everyone who enters or exits the barn is required to change into farm-specific clothing and boots
- Only the employees at the hog farm are required to change into farm-specific clothing and boots
- Only employees handling pigs are required to change into farm-specific clothing and boots
- Only visitors are required to change into farm-specific clothing and boots
- No one is required to change into farm-specific clothing and boots

Page Break

**Q79. Do you have a comparable facility (as illustrated in the picture above) at your hog farm?**

- Yes
- No

**Q80. Thinking of the general layout of most of your barns, and your barn biosecurity, check all the options that apply to your barns.**

- Footbath at the barn entry
- A dirty area that holds dirty clothes, and dirty boots
- A clean area to put on farm-specific coveralls/boots
- A clear demarcation (e.g., bench, counter, etc.) between the dirty and clean areas
- Hand-wash basin in the dirty area
- Hand-wash basin in the clean area
- Toilet
- Shower
- None of the above

**Q81. Do you use separate barn equipment (e.g. cart, feed dispenser, cleaning equipment, etc.) for each barn?**

- Yes
- No

**Q82. Do you share your farm equipment (e.g., tractors, feed trucks) with other livestock farms (including other hog farms)?**

- Yes
- No

*Display This Question:*

*If Do you share your farm equipment (e.g., tractors, feed trucks) with other livestock farms (includ... = Yes*

**Q83. Do you clean and disinfect shared farm equipment before use or after return?**

- Yes
- No

**Q84. How do you manage the manure at your hog farm? (Check all that apply)**

- On-site composting
- On-site manure storage deep pits, tanks, or lagoons
- Land application of manure to your farmland
- Land application of manure to another farmland
- Use for biodiesel only for your farm
- Use for biodiesel to other farms
- Store and sell for commercial use
- Other, Please Specify ________________________________________________

**Q85. How do you dispose of your deadstock at your hog farm? (Check all that apply)**

- Burial on site
- Incineration on-site
- Incineration off-site
- Rendering off-site
- Composting on-site
- Other, Please Specify ________________________________________________

**Q86. Do you have a separate holding location (other than pig barn) for deadstock at your hog farm?**

- Yes
- No

**Q87. How do you plan to dispose of dead stock in the event of multiple deaths (more than usual) due to a major disease outbreak at your farm? (Check all that apply)**

- Composting on-site
- Burial on-site
- Rendering
- Incineration
- I do not know
- Other, Please Specify________________________________________________

**Q88. How do you get feed for your hog farm?**

- Delivered by a commercial feed supplier/hauler
- Farm personnel haul feed from a commercial feed supplier/ local feed mill
- Produce at your farm
- Other, Please Specify ________________________________________________

**Q89. Which of the following cleaning protocol do you follow after moving out your stock from pig barns/pens?**

- Do not clean
- Remove only the visible dirt
- Clean with warm/normal water to remove visible dirt
- Clean with water and then disinfect before moving the new stock
- Other, Please Specify ________________________________________________

**Q90. What is the water source for pigs at your hog farm? *(Check all that apply)***

- Public water
- Well
- Surface water (Pond, river, lake)

*Display This Question:*

*If What is the water source for pigs at your hog farm? (Check all that apply) = Well*

*Or What is the water source for pigs at your hog farm? (Check all that apply) = Surface water (Pond, river, lake)*

**Q91. Do you test the water for bacteria at your hog farm?**

- Yes
- No

**Q92. Do you clean hog drinking water systems (e.g., drinkers, water lines) at your hog farm?**

- Yes
- No

**Q93. Which of the following animals/pest control measures do you have for the barn(s) where pigs are kept? *(Check all that apply)***

- Wild animal exclusion or control
- Bird exclusion or control
- Rodent control
- Restrict Pet entry (no dogs, cats, etc. are allowed in pig barns)

**End of Block: Practices related to the movement of people at your farm**

---

**Start of Block: Practices related to animal movements onto your farm**

## How about the movements of animals onto and off your hog farm?

**Q94. Were new pigs introduced/brought to your hog farm in the last 12 months?**

- Yes
- No

*Skip To: Q99 If Were new pigs introduced/brought to your hog farm in the last 12 months? = No*

**Q95. From where did you buy/obtain new pigs for your hog farm in the last 12 months? (Check all that apply)**

- From sources **within** Illinois
- From sources **outside** Illinois **within** the United States
- From sources **outside** of the United States
- Other, Please Specify ________________________________________________

*Display This Question:*

*If From where did you buy/obtain new pigs for your hog farm in the last 12 months? (Check all that a... = From sources <strong>outside </strong> of the United States*

**Q96. Do you import new pigs for your hog farm from Canada?**

- Yes
- No

*Display This Question:*

*If From where did you buy/obtain new pigs for your hog farm in the last 12 months? (Check all that a... = From sources <strong>outside</strong> Illinois <strong>within</strong> the United States*

**Q97. From which US State did you buy/obtain pigs for your hog farm in the last 12 months? (Check all that apply)**

| | | | | |
|---|---|---|---|---|
| Alabama | Hawaii | Massachusetts | New Mexico | South Dakota |
| Alaska | Idaho | Michigan | New York | Tennessee |
| Arizona | Illinois | Minnesota | North Carolina | Texas |
| Arkansas | Indiana | Mississippi | North Dakota | Utah |
| California | Iowa | Missouri | Ohio | Vermont |
| Colorado | Kansas | Montana | Oklahoma | Virginia |
| Connecticut | Kentucky | Nebraska | Oregon | Washington |
| Delaware | Louisiana | Nevada | Pennsylvania | West Virginia |
| Florida | Maine | New Hampshire | Rhode Island | Wisconsin |
| Georgia | Maryland | New Jersey | South Carolina | Wyoming |

*Display This Question:*

*If From where did you buy/obtain new pigs for your hog farm in the last 12 months? (Check all that a... = From sources <strong>within</strong> Illinois*

*Or From where did you buy/obtain new pigs for your hog farm in the last 12 months? (Check all that a... = Other, Please Specify*

**Q98. From which Illinois county did you buy/obtain most of the new pigs for your hog farm in the last 12 months?**

County

| | | | | | | |
|---|---|---|---|---|---|---|
| Adams | Coles | Fulton | Jo Daviess | Madison | Ogle | Scott |
| Alexander | Cook Crawford | Gallatin | Johnson | Marion Marshall | Peoria | Shelby |
| Bond | Cumberland | Greene | Kane | Mason Massac | Perry | St. Clair |
| Boone | De Witt | Grundy | Kankakee | McDonough | Piatt | Stark |
| Brown | DeKalb | Hamilton | Kendall | McHenry | Pike | Stephenson |
| Bureau | Douglas | Hancock | Knox | McLean | Pope | Tazewell |
| Calhoun | DuPage | Hardin | Lake | Menard | Pulaski | Union |
| Carroll | Edgar | Henderson | LaSalle | Mercer Monroe | Putnam | Vermilion |
| Cass | Edwards | Henry | Lawrence | Montgomery | Randolph | Wabash |
| Champaign | Effingham | Iroquois | Lee | Morgan | Richland | Warren |
| Christian | Fayette | Jackson | Livingston | Moultrie | Rock Island | Washington |
| Clark | Ford | Jasper | Logan | | Saline | Wayne |
| Clay | Franklin | Jefferson | Macon | | Sangamon | White |
| Clinton | | Jersey | Macoupin | | Schuyler | Whiteside |
| | | | | | | Will |
| | | | | | | Williamson |
| | | | | | | Winnebago |
| | | | | | | Woodford |

**Q99. In general, do you buy/obtain pigs only from farms with known disease status (e.g. pigs having a health certificate, vaccination records, etc.)?**

- Yes
- No

**Q100. In general, do you assess your pig's health within 24 hours of their arrival at your hog farm?**

- Yes
- No

**Q101. How are pigs transported to your hog farm?**

- Use your vehicle
- Hire transport
- Others, Please Specify ________________________________________________

**Q102. How do you transport your pigs to another farm(s) or market(s)?**

- Use your vehicle
- Hire transport
- Others, Please Specify ________________________________________________

**Q103. Are animal transport vehicles required to be washed and disinfected before and after use?**

- Always
- Most of the times
- Sometimes
- Rarely
- Never

\

**Q104. Do you isolate newly obtained pigs or pigs that have moved off your hog farm and returned?** *(**Isolation** is a procedure that is intended to keep the newly purchased pigs and/or pigs who have traveled outside of the farm facilities and returned in an isolation pen for a designated period before mixing them with the existing stock.)*

- Always
- Most of the times
- Sometimes
- Rarely
- Never

*Display This Question:*

*If Note: Quarantine is a procedure that is intended to keep the newly purchased pigs and/or pigs who... != Never*

**Q105. For how many weeks do you isolate the newly obtained pigs or pigs that you have moved off your hog farm and returned?**

- Less than 1 week
- 1 week
- 2 weeks
- 3 weeks
- 4 or more weeks

Note: An **Isolation pen** is a designated space used for keeping pigs who are new or have traveled outside of the farm and returned, separated from the existing stock to prevent the introduction of disease into naïve herds. Isolation pens should be physically separated from any buildings used for other livestock.

**Q106. Do you have an isolation barn your hog farm?** (An Isolation barn is a designated space used for keeping pigs who are new or have traveled outside of the farm and returned, separated from the existing stock to prevent the introduction of disease into naïve herds. Isolation pens should be physically separated from any buildings used for other livestock.)

- Yes
- No

**End of Block: Practices related to animal movements onto your farm**

---

**Start of Block: Animal Health Management**

## How about the animal health management practices at your hog farm? Let's talk briefly about that.

**Q107. Do you have a Veterinarian-Client-Patient-Relationship (VCPR) for monitoring the health of pigs at your hog farm(s)?** *(A **veterinarian-client-patient relationship***

***(VCPR)*** *is the basis for interaction among veterinarians, their clients, and their patients and is critical to the health of your animal.)*

- Yes
- No

*Display This Question:*

*If Note: A veterinarian-client-patient relationship (VCPR) is the basis for interaction among veteri... = No*

**Q108. How do you treat pigs if/when they get sick at your hog farm?**

- Seek advice from a veterinarian
- Seek advice from fellow hog producers
- Treat on our own with the help of our farm staff
- Others, Please Specify ________________

**Q109. Are sick pigs separated from the healthy ones at your hog farm?**

- Yes
- No

*Display This Question:*

*If Note: A veterinarian-client-patient relationship (VCPR) is the basis for interaction among veteri... = No*

**Q110. Do you keep written records of pigs' health status at your hog farm?**

- Yes
- No

**Q111. Are your pigs vaccinated for any diseases?**

- Yes
- No

**Q112. Are you testing the pigs for infectious diseases at your hog farm?**

- Yes
- No

## End of Block: Animal Health Management

---

## Start of Block: Previous Disease Outbreaks

## Tell us briefly about the disease status of your farm.

**Q113. Has your hog farm had any swine disease outbreaks in the last 3 years?**

- Yes
- No
- Prefer not to say

*Skip To: End of Block If Has your hog farm had any swine disease outbreaks in the last 3 years? = Prefer not to say*

*Display This Question:*

*If Has your hog farm had any swine disease outbreaks in the last 3 years? = Yes*

**Q114. Has your hog farm(s) had any of the following disease outbreaks indicated below in the last 3 years? (Check all that apply).**

| | |
|---|---|
| PRRS | Porcine Reproductive and Respiratory Disease |
| PEDV | Porcine Epidemic Diarrhoea Virus |

- PRRS
- PEDV
- Influenza
- Mycoplasmosis
- Rotavirus
- Seneca Valley Virus
- Colibacillosis (E.coli)
- Others, Please Specify ________________

---

*Display This Question:*

*If If Has your hog farm(s) had any of the following disease outbreaks indicated below in the last 3 years? (Check all that apply).    PRRS  Porcine Reproductive and Respir... q://QID85/SelectedChoicesCount Is Greater Than or Equal to 1*

*Carry Forward Selected Choices - Entered Text from "Has your hog farm(s) had any of the following disease outbreaks indicated below in the last 3 years? (Check all that apply). PRRS Porcine Reproductive and Respiratory Disease PEDV Porcine Epidemic Diarrhoea Virus "*

X→

**Q115. Please indicate the time of occurrence of the disease outbreak. (Check all that apply)**

| | In the last 6 months | In the last 7-12 months | In the last 13-18 months | In last 19-36 months |
|---|---|---|---|---|
| PRRS | ○ | ○ | ○ | ○ |
| PEDV | ○ | ○ | ○ | ○ |
| Influenza | ○ | ○ | ○ | ○ |
| Mycoplasmosis | ○ | ○ | ○ | ○ |
| Rotavirus | ○ | ○ | ○ | ○ |
| Seneca Valley Virus | ○ | ○ | ○ | ○ |
| Colibacillosis (E.coli) | ○ | ○ | ○ | ○ |
| Others, Please Specify | ○ | ○ | ○ | ○ |

**End of Block: Previous Disease Outbreaks**

---

**Start of Block: mf**

* **Q38. How many farms do you operate in Illinois? (Please specify their number)**

________________________________

**Q39. Is your primary residence located at any of your hog farm(s)?**

- Yes
- No

*Display This Question:*

*If Is your primary residence located at any of your hog farm(s)? = Yes*

**Q40. Does the primary residence share the driveway or entrance with your hog farm?**

- Yes
- No

NOTE:
**Farrow to finish farm** (Involves all stages of production, raising pigs from breeding through finishing to market weights)
**Farrow to wean farm** (Involves raising pigs from breeding through marketing 10- to 15-pound weaned pigs for nursery-grow-finish farms) **Farrow to nursery/feeder farm** (Involves raising pigs from breeding through marketing 40- to 60-pound feeder pigs for finishing farms) **Wean-to-finish farm** (Involves raising weaned pigs and finishing them to market weights)
**Finishing farm** (Involves raising 40- to 60-pound feeder pigs and finish them to market weight).
**Nursery farm** (involves raising weaned pigs through 40- to 60-pound feeder pigs)

**Q41. How would you describe your hog farms? (Check all that apply)**

- Farrow to finish farm
- Farrow to wean farm
- Farrow to nursery/feeder farm
- Wean-to-finish farm
- Finishing farm
- Nursery farm
- Other, Please specify ________________________________________________

*Skip To: (*) If Condition: Selected Count Is Equal to 1. Skip To: Think about your newest ${q://QID42/....*

*Carry Forward Selected Choices - Entered Text from "NOTE: Farrow to finish farm (Involves all stages of production, raising pigs from breeding through finishing to market weights) Farrow to wean farm (Involves raising pigs from breeding through marketing 10- to 15-pound weaned pigs for nursery-grow-finish farms) Farrow to nursery/feeder farm (Involves raising pigs from breeding through marketing 40- to 60-pound feeder pigs for finishing farms) Wean-to-finish farm (Involves raising weaned pigs and finishing them to market weights) Finishing farm (Involves raising 40- to 60-pound feeder pigs and finish them to market weight).*

*Nursery farm (involves raising weaned pigs through 40- to 60-pound feeder pigs)        How would you describe your hog farms? (Check all that apply)  "*

**Q42. Among the ${ QHow many farms do you operate in Illinois? (Please specify their number)/ChoiceTextEntryValue} farms that you have in Illinois, please indicate the number of each type of your hog farms.**

Farrow to finish farm : ________
Farrow to wean farm : ________
Farrow to nursery/feeder farm : ________
Wean-to-finish farm : ________
Finishing farm : ________
Nursery farm : ________  (6)
Other, Please specify : ________  (7)
Total : ________

---

*Carry Forward Selected Choices - Entered Text from "NOTE:  Farrow to finish farm (Involves all stages of production, raising pigs from breeding through finishing to market weights)   Farrow to wean farm (Involves raising pigs from breeding through marketing 10- to 15-pound weaned pigs for nursery-grow-finish farms)  Farrow to nursery/feeder farm (Involves raising pigs from breeding through marketing 40- to 60-pound feeder pigs for finishing farms)  Wean-to-finish farm (Involves raising weaned pigs and finishing them to market weights) Finishing farm (Involves raising 40- to 60-pound feeder pigs and finish them to market weight). Nursery farm (involves raising weaned pigs through 40- to 60-pound feeder pigs)        How would you describe your hog farms? (Check all that apply)  "*

**Q43. Which type of hog farm is the newest of all your hog farms?**

- Farrow to finish farm
- Farrow to wean farm
- Farrow to nursery/feeder farm
- Wean-to-finish farm
- Finishing farm
- Nursery farm
- Other, Please Specify

---

Page Break

## Think about your newest ${Q43/ChoiceGroup/SelectedChoices} and answer the following question.

*Display This Question:*

*If If NOTE:  Farrow to finish farm (Involves all stages of production, raising pigs from breeding through finishing to market weights)   Farrow to wean farm (Involves raisin... q://QID41/SelectedChoicesCount Is Equal to  1*

## (*) Think about your newest ${Q42/ChoiceGroup/SelectedChoicesTextEntry} and answer the following questions.

*Display This Question:*

*If If NOTE:  Farrow to finish farm (Involves all stages of production, raising pigs from breeding through finishing to market weights)   Farrow to wean farm (Involves raisin... q://QID41/SelectedChoicesCount Is Equal to  1*

**Q44. In which Illinois county is your newest ${Q42/ChoiceGroup/SelectedChoicesTextEntry} located?**

County

| | | | | | | |
|---|---|---|---|---|---|---|
| Adams<br>Alexander<br>Bond<br>Boone<br>Brown<br>Bureau<br>Calhoun<br>Carroll<br>Cass<br>Champaign<br>Christian<br>Clark<br>Clay<br>Clinton | Coles<br>Cook Crawford<br>Cumberland<br>De Witt<br>DeKalb<br>Douglas<br>DuPage<br>Edgar<br>Edwards<br>Effingham<br>Fayette<br>Ford<br>Franklin | Fulton<br>Gallatin<br>Greene<br>Grundy<br>Hamilton<br>Hancock<br>Hardin<br>Henderson<br>Henry<br>Iroquois<br>Jackson<br>Jasper<br>Jefferson<br>Jersey | Jo Daviess<br>Johnson<br>Kane<br>Kankakee<br>Kendall<br>Knox<br>Lake<br>LaSalle<br>Lawrence<br>Lee<br>Livingston<br>Logan<br>Macon<br>Macoupin | Madison<br>Marion Marshall<br>Mason Massac<br>McDonough<br>McHenry<br>McLean<br>Menard<br>Mercer Monroe<br>Montgomery<br>Morgan<br>Moultrie | Ogle<br>Peoria<br>Perry<br>Piatt<br>Pike<br>Pope<br>Pulaski<br>Putnam<br>Randolph<br>Richland<br>Rock Island<br>Saline<br>Sangamon<br>Schuyler | Scott<br>Shelby<br>St. Clair<br>Stark<br>Stephenson<br>Tazewell<br>Union<br>Vermilion<br>Wabash<br>Warren<br>Washington<br>Wayne<br>White<br>Whiteside<br>Will<br>Williamson<br>Winnebago<br>Woodford |

*Display This Question:*

*If If NOTE:  Farrow to finish farm (Involves all stages of production, raising pigs from breeding through finishing to market weights)   Farrow to wean farm (Involves raisin... q://QID41/SelectedChoicesCount Is Greater Than  1*

**Q45. In which Illinois county is your newest ${Q43/ChoiceGroup/SelectedChoices} located?**

County

| | | | | | | |
|---|---|---|---|---|---|---|
| Adams<br>Alexander<br>Bond<br>Boone<br>Brown<br>Bureau<br>Calhoun<br>Carroll<br>Cass<br>Champaign<br>Christian<br>Clark<br>Clay<br>Clinton | Coles<br>Cook Crawford<br>Cumberland<br>De Witt<br>DeKalb<br>Douglas<br>DuPage<br>Edgar<br>Edwards<br>Effingham<br>Fayette<br>Ford<br>Franklin | Fulton<br>Gallatin<br>Greene<br>Grundy<br>Hamilton<br>Hancock<br>Hardin<br>Henderson<br>Henry<br>Iroquois<br>Jackson<br>Jasper<br>Jefferson<br>Jersey | Jo Daviess<br>Johnson<br>Kane<br>Kankakee<br>Kendall<br>Knox<br>Lake<br>LaSalle<br>Lawrence<br>Lee<br>Livingston<br>Logan<br>Macon<br>Macoupin | Madison<br>Marion Marshall<br>Mason Massac<br>McDonough<br>McHenry<br>McLean<br>Menard<br>Mercer Monroe<br>Montgomery<br>Morgan<br>Moultrie | Ogle<br>Peoria<br>Perry<br>Piatt<br>Pike<br>Pope<br>Pulaski<br>Putnam<br>Randolph<br>Richland<br>Rock Island<br>Saline<br>Sangamon<br>Schuyler | Scott<br>Shelby<br>St. Clair<br>Stark<br>Stephenson<br>Tazewell<br>Union<br>Vermilion<br>Wabash<br>Warren<br>Washington<br>Wayne<br>White<br>Whiteside<br>Will<br>Williamson<br>Winnebago<br>Woodford |

**Q46. Do you have a Premise Identification Number for your newest ${Q43/ChoiceGroup/SelectedChoicesTextEntry}? (Premises Identification Number** (Premises ID or Prem ID or PIN) is the identification of a location where livestock is raised, housed, or pass through during commerce and is assigned by the Illinois Department of Agriculture which allows animal health officials to quickly and precisely identify where animals are located in the event of an animal health emergency.)

- Yes
- No

**Q47. How many other livestock farms are situated within a 3-mile radius of your newest hog farm?**

- None
- 1 farm
- 2-3 farms
- 4-5 farms
- More than 5 farms

* **Q48. How many pigs do you have on your newest hog farm as of today? (Please specify their number)**

________________________________________

**Q49. Do you keep any other type of livestock/poultry species on your newest hog farm?**

- Yes
- No

*Display This Question:*

*If Do you keep any other type of livestock/poultry species on your hog farm? = Yes*

**Q50. What type and number of livestock and/or poultry do you keep on your farm? *(Check all that apply)***

- Cattle
- Sheep and/or Goat
- Poultry
- Other, Please Specify ________________________________________________

**Q51. Which type of pig housing is used at your newest hog farm?**

- Total confinement with mechanical ventilation
- Total confinement with natural ventilation
- Open building with outside access for pigs
- Other, Please Specify ________________________________________________

*Display This Question:*

*If Which type of pig housing is used at your hog farm? = Total confinement with mechanical ventilation*

**Q52. Do you have an air filtration unit installed in the barn(s) that house the pigs?**

- Yes
- No

**Q53. How many barn(s) do you have that house pigs at your newest hog farm? (Please specify their number)**

________________________

**Q54. How many barn(s) are less than 10 years old that house pigs at your newest hog farm?**

- None
- 1 barn
- 2 barns
- 3-4 barns
- More than 4 barns

**Q55. Do you have a Secure Pork Supply Plan for your newest hog farm?** (A **Secure Pork Supply Plan** lists current biosecurity practices implemented at your hog operation and provides opportunities to voluntarily prepare before a foreign animal disease (FAD) outbreak.)

- Yes
- No

*Display This Question:*

*If Note: A Secure Pork Supply Plan lists current biosecurity practices implemented at your hog opera... = No*

**Q56. Have you had a biosecurity assessment done for your newest hog farm?** *(**Biosecurity assessment** evaluates current practices and identifies how to reduce disease introductions and/or spread. Biosecurity assessments can be done by you, your veterinarian, another experienced swine producer, a biosecurity officer, or other appropriately knowledgeable people.)*

- Yes
- No

*Display This Question:*

*If Note: Biosecurity assessment evaluates current practices and identifies how to reduce disease int... = Yes*

**Q57. When was the last biosecurity assessment done for your newest hog farm?**

- 1-6 months ago
- 7-12 months ago
- 13-24 months ago
- More than 24 months ago

**End of Block: mf**

**Start of Block: MF Practices related to the movement of people at your farm**

## Let's dive into the movement of people onto your hog newest farm.

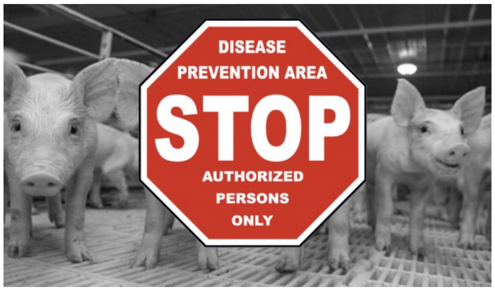


**Q58. Do you have signage (similar to the one shown above) at your newest hog farm entrance indicating "a biosecure area"?**

- Yes
- No

**Q59. Do you have gates or barriers to control entry at the main entrance of your newest hog farm?**

- Yes
- No

*Display This Question:*

*If Do you have gates or barriers to control entry at the main entrance of your hog farm? = Yes*

**Q60. Do you restrict entry to your hog farm using a closed gate, when the facility is not attended to?**

- Yes
- No

**Q61. Do you have a separate entrance for vehicles, which collect and transport farm waste (e.g., manure and/or deadstock) from your newest hog farm?**

- Yes
- No

**Q62. How many entry point(s) do you have to your newest hog farm?**

- 1
- 2
- More than 2

**Q63. Do you have a logbook that records every entry (e.g., identity, date, time, and purpose of visit) on your hog farm?**

- Yes
- No

**Q64. Do you allow vehicles to enter your newest hog farm and park within 10 feet of the barn(s) where pigs are housed?**

- Yes
- No

**Q65. Do you require vehicles (including trucks) to be washed and checked to be free of visible dirt before entering your hog farm?**

- Yes
- No

**Q66. Which of the following structures (if any) are present at your newest hog farm? (Check all that apply)**

- Designated parking area for trucks and visitors
- Loading and unloading area for pigs
- Delivery areas for feed
- Truck wash unit
- Cleaning and disinfection station
- Handwashing area
- Toilet
- Area for changing into farm-specific clothes and boots
- None of the above

**Q67. Do you have a well-defined perimeter buffer area (an outer control boundary, set up around the farm buildings to minimize potential pathogen introduction near animal housing) at your newest hog farm?**

- Yes
- No

*Skip To: Q69 If Do you have a well-defined perimeter buffer area (an outer control boundary, set up around the fa... = No*

**Q68. Which of the following facilities are located outside the perimeter buffer area? (Check all that apply)**

- A designated parking lot for non-farm/non-animal vehicles
- Loading and unloading areas
- Farm personnel/ owner's housing units
- Cleaning & Disinfection station
- Feed storage unit
- None of the above

**Q69. Do you have employees caring for your hogs at your newest hog farm?**

- Yes
- No

*Display This Question:*

*If Do you have employees caring for your hogs at your hog farm?  = No*

**Q70. Do you work at other hog farms during a given workday?**

- Yes
- No

*Display This Question:*

*If Do you have employees caring for your hogs at your hog farm?  = Yes*

**Q71. Do any of your employees work at other hog farms during a given workday?**

- Yes
- No

**Q72. Do people (yourself, employees, family members, or visitors) change into farm-specific coveralls and boots before entering your newest hog farm?**

- Yes
- No

*Display This Question:*

*If Do people (yourself, employees, family members, or visitors) change into farm-specific coveralls... = Yes*

**Q73. Do people (yourself, employees, family members or visitors) take a shower before entering your newest hog farm?**

- Yes
- No

Page Break

## Moving on, we will talk about the structures and facilities available at your newest hog farm.

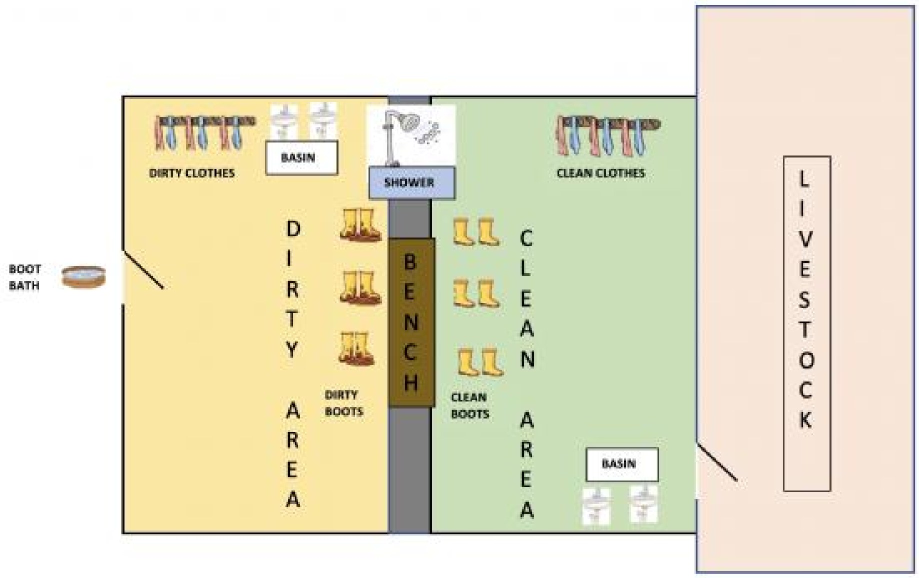


**Q74. Do you have a clearly defined line of separation (building walls separating the pigs from the outside) for each building that houses pigs?**

- Yes
- No

*Skip To: Q76 If Do you have a clearly defined line of separation (building walls separating the pigs from the out... = No*

**Q75. Do you always lock buildings where pigs are kept when no one is present to restrict the entry of unauthorized people into the barn(s)?**

- Yes
- No

**Q76. Do you have designated entry point(s) for people entering the barn where pigs are kept?**

- Yes
- No

**Q77. Do you have a logbook for records of who entered the barn where pigs are kept?**

- Yes
- No

**Q78. Do you require visitors and employees who enter the barn where pigs are housed to change into farm-specific clothing and boots?**

- Everyone who enters or exits the barn is required to change into farm-specific clothing and boots
- Only the employees at the hog farm are required to change into farm-specific clothing and boots
- Only employees handling pigs are required to change into farm-specific clothing and boots
- Only visitors are required to change into farm-specific clothing and boots
- No one is required to change into farm-specific clothing and boots

Page Break

---

**Q79. Do you have a comparable facility (as illustrated in the picture above) at your hog farm?**

- Yes
- No

**Q80. Thinking of the general layout of most of your barns, and your barn biosecurity, check all the options that apply to your barns.**

- Footbath at the barn entry
- A dirty area that holds dirty clothes, and dirty boots
- A clean area to put on farm-specific coveralls/boots
- A clear demarcation (e.g., bench, counter, etc.) between the dirty and clean areas
- Hand-wash basin in the dirty area
- Hand-wash basin in the clean area
- Toilet
- Shower
- None of the above

**Q81. Do you use separate barn equipment (e.g. cart, feed dispenser, cleaning equipment, etc.) for each barn?**

- Yes
- No

**Q82. Do you share your farm equipment (e.g., tractors, feed trucks) with other livestock farms (including other hog farms)?**

- Yes
- No

*Display This Question:*

*If Do you share your farm equipment (e.g., tractors, feed trucks) with other livestock farms (includ... = Yes*

**Q83. Do you clean and disinfect shared farm equipment before use or after return?**

- Yes
- No

**Q84. How do you manage the manure at your newest hog farm? (Check all that apply)**

- On-site composting
- On-site manure storage deep pits, tanks, or lagoons
- Land application of manure to your farmland
- Land application of manure to another farmland
- Use for biodiesel only for your farm
- Use for biodiesel to other farms
- Store and sell for commercial use
- Other, Please Specify ________________________________________________

**Q85. How do you dispose of your deadstock at your newest hog farm? (Check all that apply)**

- Burial on site
- Incineration on-site
- Incineration off-site
- Rendering off-site
- Composting on-site
- Other, Please Specify ________________________________________________

**Q86. Do you have a separate holding location (other than pig barn) for deadstock at your newest hog farm?**

- Yes
- No

**Q87. How do you plan to dispose of dead stock in the event of multiple deaths (more than usual) due to a major disease outbreak at your newest hoh farm? (Check all that apply)**

- Composting on-site
- Burial on-site
- Rendering
- Incineration
- I do not know
- Other, Please Specify________________________________________________

**Q88. How do you get feed for your newest hog farm?**

- Delivered by a commercial feed supplier/hauler
- Farm personnel haul feed from a commercial feed supplier/ local feed mill
- Produce at your farm
- Other, Please Specify ________________________________________________

**Q89. Which of the following cleaning protocol do you follow after moving out your stock from pig barns/pens?**

- Do not clean
- Remove only the visible dirt
- Clean with warm/normal water to remove visible dirt
- Clean with water and then disinfect before moving the new stock
- Other, Please Specify ________________________________________________

**Q90. What is the water source for pigs at your newest hog farm? *(Check all that apply)***

- Public water
- Well
- Surface water (Pond, river, lake)

*Display This Question:*

*If What is the water source for pigs at your hog farm? (Check all that apply) = Well*

*Or What is the water source for pigs at your hog farm? (Check all that apply) = Surface water (Pond, river, lake)*

**Q91. Do you test the water for bacteria at your newest hog farm?**

- Yes
- No

**Q92. Do you clean hog drinking water systems (e.g., drinkers, water lines) at your newest hog farm?**

- Yes
- No

**Q93. Which of the following animals/pest control measures do you have for the barn(s) where pigs are kept? *(Check all that apply)***

- Wild animal exclusion or control
- Bird exclusion or control
- Rodent control
- Restrict Pet entry (no dogs, cats, etc. are allowed in pig barns)

**End of Block: Practices related to the movement of people at your farm**

---

**Start of Block: Practices related to animal movements onto your farm**

## How about the movements of animals onto and off your newest hog farm?

**Q94. Were new pigs introduced/brought to your newest hog farm in the last 12 months?**

- Yes
- No

*Skip To: Q99 If Were new pigs introduced/brought to your hog farm in the last 12 months? = No*

**Q95. From where did you buy/obtain new pigs for your newest hog farm in the last 12 months? (Check all that apply)**

- From sources **within** Illinois
- From sources **outside** Illinois **within** the United States
- From sources **outside** of the United States
- Other, Please Specify ________________________________________________

*Display This Question:*

*If From where did you buy/obtain new pigs for your hog farm in the last 12 months? (Check all that a... = From sources <strong>outside </strong> of the United States*

**Q96. Do you import new pigs for your newest hog farm from Canada?**

- Yes
- No

*Display This Question:*

*If From where did you buy/obtain new pigs for your hog farm in the last 12 months? (Check all that a... = From sources <strong>outside</strong> Illinois <strong>within</strong> the United States*

**Q97. From which US State did you buy/obtain pigs for your newest hog farm in the last 12 months? (Check all that apply)**

| | | | | |
|---|---|---|---|---|
| Alabama | Hawaii | Massachusetts | New Mexico | South Dakota |
| Alaska | Idaho | Michigan | New York | Tennessee |
| Arizona | Illinois | Minnesota | North Carolina | Texas |
| Arkansas | Indiana | Mississippi | North Dakota | Utah |
| California | Iowa | Missouri | Ohio | Vermont |
| Colorado | Kansas | Montana | Oklahoma | Virginia |
| Connecticut | Kentucky | Nebraska | Oregon | Washington |
| Delaware | Louisiana | Nevada | Pennsylvania | West Virginia |
| Florida | Maine | New Hampshire | Rhode Island | Wisconsin |
| Georgia | Maryland | New Jersey | South Carolina | Wyoming |

*Display This Question:*

*If From where did you buy/obtain new pigs for your hog farm in the last 12 months? (Check all that a... = From sources <strong>within</strong> Illinois*

*Or From where did you buy/obtain new pigs for your hog farm in the last 12 months? (Check all that a... = Other, Please Specify*

**Q98. From which Illinois county did you buy/obtain most of the new pigs for your newest hog farm in the last 12 months?**

County

| | | | | | | |
|---|---|---|---|---|---|---|
| Adams | Coles | Fulton | Jo Daviess | Madison | Ogle | Scott |
| Alexander | Cook Crawford | Gallatin | Johnson | Marion Marshall | Peoria | Shelby |
| Bond | Cumberland | Greene | Kane | Mason Massac | Perry | St. Clair |
| Boone | De Witt | Grundy | Kankakee | McDonough | Piatt | Stark |
| Brown | DeKalb | Hamilton | Kendall | McHenry | Pike | Stephenson |
| Bureau | Douglas | Hancock | Knox | McLean | Pope | Tazewell |
| Calhoun | DuPage | Hardin | Lake | Menard | Pulaski | Union |
| Carroll | Edgar | Henderson | LaSalle | Mercer Monroe | Putnam | Vermilion |
| Cass | Edwards | Henry | Lawrence | Montgomery | Randolph | Wabash |
| Champaign | Effingham | Iroquois | Lee | Morgan | Richland | Warren |
| Christian | Fayette | Jackson | Livingston | Moultrie | Rock Island | Washington |
| Clark | Ford | Jasper | Logan | | Saline | Wayne |
| Clay | Franklin | Jefferson | Macon | | Sangamon | White |
| Clinton | | Jersey | Macoupin | | Schuyler | Whiteside |
| | | | | | | Will |
| | | | | | | Williamson |

| | | | | | | |
|---|---|---|---|---|---|---|
| | | | | | | Winnebago<br>Woodford |

**Q99. In general, do you buy/obtain pigs only from farms with known disease status (e.g. pigs having a health certificate, vaccination records, etc.)?**

- Yes
- No

**Q100. In general, do you assess your pig's health within 24 hours of their arrival at your newest hog farm?**

- Yes
- No

**Q101. How are pigs transported to your newest hog farm?**

- Use your vehicle
- Hire transport
- Others, Please Specify ________________________________

**Q102. How do you transport your pigs to another farm(s) or market(s)?**

- Use your vehicle
- Hire transport
- Others, Please Specify ________________________________

**Q103. Are animal transport vehicles required to be washed and disinfected before and after use?**

- Always
- Most of the times
- Sometimes
- Rarely
- Never

**Q104. Do you isolate newly obtained pigs or pigs that have moved off your newest hog farm and returned?** *(**Isolation** is a procedure that is intended to keep the newly purchased*

*pigs and/or pigs who have traveled outside of the farm facilities and returned in an isolation pen for a designated period before mixing them with the existing stock.)*

- Always
- Most of the times
- Sometimes
- Rarely
- Never

*Display This Question:*

*If Note: Quarantine is a procedure that is intended to keep the newly purchased pigs and/or pigs who... != Never*

**Q105. For how many weeks do you isolate the newly obtained pigs or pigs that you have moved off your newest hog farm and returned?**

- Less than 1 week
- 1 week
- 2 weeks
- 3 weeks
- 4 or more weeks

**Q106. Do you have an isolation barn at your newest hog farm?** (An Isolation barn is a designated space used for keeping pigs who are new or have traveled outside of the farm and returned, separated from the existing stock to prevent the introduction of disease into naïve herds. Isolation pens should be physically separated from any buildings used for other livestock.)

- Yes
- No

**End of Block: Practices related to animal movements onto your farm**

---

**Start of Block: Animal Health Management**

## How about the animal health management practices at your hog farm? Let's talk briefly about that.

**Q107. Do you have a Veterinarian-Client-Patient-Relationship (VCPR) for monitoring the health of pigs at your newest hog farm(s)?** *(A **veterinarian-client-patient relationship***

*(**VCPR**) is the basis for interaction among veterinarians, their clients, and their patients and is critical to the health of your animal.)*

- Yes
- No

*Display This Question:*

*If Note: A veterinarian-client-patient relationship (VCPR) is the basis for interaction among veteri... = No*

**Q108. How do you treat pigs if/when they get sick at your newest hog farm?**

- Seek advice from a veterinarian
- Seek advice from fellow hog producers
- Treat on our own with the help of our farm staff
- Others, Please Specify ________________

**Q109. Are sick pigs separated from the healthy ones at your newest hog farm?**

- Yes
- No

*Display This Question:*

*If Note: A veterinarian-client-patient relationship (VCPR) is the basis for interaction among veteri... = No*

**Q110. Do you keep written records of pigs' health status at your newest hog farm?**

- Yes
- No

**Q111. Are your pigs vaccinated for any diseases?**

- Yes
- No

**Q112. Are you testing the pigs for infectious diseases at your newest hog farm?**

- Yes
- No

**End of Block: Animal Health Management**

---

**Start of Block: Previous Disease Outbreaks**

## Tell us briefly about the disease status of your newest hog farm.

### Q113. Has your newest hog farm had any swine disease outbreaks in the last 3 years?

- Yes
- No
- Prefer not to say

*Skip To: End of Block If Has your hog farm had any swine disease outbreaks in the last 3 years? = Prefer not to say*

*Display This Question:*

*If Has your hog farm had any swine disease outbreaks in the last 3 years? = Yes*

**Q114. Has your newest hog farm(s) had any of the following disease outbreaks indicated below in the last 3 years? (Check all that apply).**

| | |
|---|---|
| PRRS | Porcine Reproductive and Respiratory Disease |
| PEDV | Porcine Epidemic Diarrhoea Virus |

- PRRS
- PEDV
- Influenza
- Mycoplasmosis
- Rotavirus
- Seneca Valley Virus
- Colibacillosis (E.coli)
- Others, Please Specify ________________

*Display This Question:*

*If If Has your hog farm(s) had any of the following disease outbreaks indicated below in the last 3 years? (Check all that apply).    PRRS  Porcine Reproductive and Respir... q://QID85/SelectedChoicesCount Is Greater Than or Equal to 1*

*Carry Forward Selected Choices - Entered Text from "Has your hog farm(s) had any of the following disease outbreaks indicated below in the last 3 years? (Check all that apply). PRRS Porcine Reproductive and Respiratory Disease PEDV Porcine Epidemic Diarrhoea Virus "*

X→

**Q115. Please indicate the time of occurrence of the disease outbreak. (Check all that apply)**

| | In the last 6 months | In the last 7-12 months | In the last 13-18 months | In last 19-36 months |
|---|---|---|---|---|
| PRRS | ○ | ○ | ○ | ○ |
| PEDV | ○ | ○ | ○ | ○ |
| Influenza | ○ | ○ | ○ | ○ |
| Mycoplasmosis | ○ | ○ | ○ | ○ |
| Rotavirus | ○ | ○ | ○ | ○ |
| Seneca Valley Virus | ○ | ○ | ○ | ○ |
| Colibacillosis (E.coli) | ○ | ○ | ○ | ○ |
| Others, Please Specify | ○ | ○ | ○ | ○ |

**End of Block: Previous Disease Outbreaks**

**Start of Block: Multi-farm**

## So you mentioned earlier, you have ${Q42/ChoiceTextEntryValue} hog farms.

**Q116. According to you, how different are the structure and management practices at your other hog farm(s) from your newest one?**

- Very different
- Slightly different
- Not very different

*Display This Question:*

*If If NOTE:  Farrow to finish farm (Involves all stages of production, raising pigs from breeding through finishing to market weights)   Farrow to wean farm (Involves raisin... q://QID41/SelectedChoicesCount Is Greater Than  1*

*Carry Forward Unselected Choices from "Which type of hog farm is the newest of all your hog farms?"*

**Q117. Which of your hog farm(s) differs the most from your newest hog farm?**

- Farrow to finish farm
- Farrow to wean farm
- Farrow to nursery/feeder farm
- Wean-to-finish farm
- Finishing farm
- Nursery farm
- Other, Please Specify

Page Break

Display This Question:

*If If NOTE:  Farrow to finish farm (Involves all stages of production, raising pigs from breeding through finishing to market weights)   Farrow to wean farm (Involves raisin... q://QID41/SelectedChoicesCount Is Equal to  1*

**Please answer the following questions thinking about your ${Q41/ChoiceGroup/SelectedChoicesTextEntry} which differs the most from your newest ${Q41/ChoiceGroup/SelectedChoicesTextEntry} in terms of animal number or structure or management practices. Let's call this farm, Farm 2:**

*Display This Question:*

*If If NOTE:  Farrow to finish farm (Involves all stages of production, raising pigs from breeding through finishing to market weights)   Farrow to wean farm (Involves raisin... q://QID41/SelectedChoicesCount Is Equal to  1*

**Q118. In which of the following aspects does your Farm 2 differ from your newest hog farm? (Check all that apply)**

- Number of pigs
- Type of housing
- Biosecurity plans and assessment

*Display This Question:*

*If If NOTE:  Farrow to finish farm (Involves all stages of production, raising pigs from breeding through finishing to market weights)   Farrow to wean farm (Involves raisin... q://QID41/SelectedChoicesCount Is Greater Than  1*

**You say your ${Q117/ChoiceGroup/SelectedChoices} differs from your newest farm. Let us call this farm, Farm 2. Please answer the flowing question thinking about your Farm 2:**

*Display This Question:*

*If If NOTE:  Farrow to finish farm (Involves all stages of production, raising pigs from breeding through finishing to market weights)   Farrow to wean farm (Involves raisin... q://QID41/SelectedChoicesCount Is Greater Than  1*

**Q119. In which of the following aspects does your Farm 2 differ from your newest hog farm? *(Check all that apply)***

- Number of pigs
- Type of housing
- Biosecurity plans and assessment

*Display This Question:*

*If In which of the following aspects does your Farm 2 differ from your newest farm? (Check all that... = Number of pigs*

*Or In which of the following aspects does your Farm 2 differ from your newest farm? (Check all that... = Number of pigs*

**Q120. How many pigs do you have on your Farm 2 as of today? (Please specify the number)**

________________

*Display This Question:*

*If In which of the following aspects does your Farm 2 differ from your newest farm? (Check all that... = Type of housing*

*Or In which of the following aspects does your Farm 2 differ from your newest farm? (Check all that... = Type of housing*

**Q121. What is the type of pig housing at your Farm 2?**

- Total confinement with mechanical ventilation
- Total confinement with natural ventilation
- Open building with outside access for pigs
- Other, Please Specify ________________

*Display This Question:*

*If In which of the following aspects does your Farm 2 differ from your newest farm? (Check all that... = Biosecurity plans and assessment*

**Q122. Have you had a biosecurity assessment completed for your Farm 2?**

- Yes
- No

*Display This Question:*

*If Have you had a biosecurity assessment completed for your Farm 2? = Yes*

**Q123. When was the most recent biosecurity assessment completed for your Farm 2?**

- 1-6 months ago
- 7-12 months ago
- 13-24 months ago
- More than 24 months ago

*Display This Question:*

*If In which of the following aspects does your Farm 2 differ from your newest farm? (Check all that... = Biosecurity plans and assessment*

*And In which of the following aspects does your Farm 2 differ from your newest farm? (Check all that... = Biosecurity plans and assessment*

**Q124. Do you have a Secure Pork Supply Plan for your hog farm?** (A **Secure Pork Supply Plan** lists current biosecurity practices implemented at your hog operation and provides opportunities to voluntarily prepare before a foreign animal disease (FAD) outbreak.)

- Yes
- No

*Display This Question:*

*If If NOTE:  Farrow to finish farm (Involves all stages of production, raising pigs from breeding through finishing to market weights)   Farrow to wean farm (Involves raisin... q://QID41/SelectedChoicesCount Is Equal to  1*

**Q125. Which of the following structures are present at your Farm 2? *(Check all that apply)***

- Signage at the farm entrance
- Cleaning and disinfection station
- Separate clean area (containing clean boots, coveralls) and dirty area (containing dirty boots, coveralls)
- Shower-in-shower-out facility
- Designated parking area for trucks and visitors
- Designated loading and unloading area for pigs
- Truck wash unit
- None of the above

*Display This Question:*

*If If NOTE:  Farrow to finish farm (Involves all stages of production, raising pigs from breeding through finishing to market weights)   Farrow to wean farm (Involves raisin... q://QID41/SelectedChoicesCount Is Equal to  1*

Note: "**All-In-All-Out**" is a system that keeps animals together in groups matched by age, weight, production stage, and condition. The group is moved into a phase of production together and is moved out of that phase as a group according to a production schedule. When a group moves forward, the facility is completely emptied.)

**Q126. Which of the following protocols do you follow at your Farm 2? (Check all that apply)**

- Requires people (yourself, employees, visitors) to change into farm-specific boots and/or coveralls before entering the farm
- Requires people (yourself, employees, visitors) to shower before entering the farm
- Maintains logbook for visitors at farm entry
- Requires vehicle entering the farm to be washed and free from visible dirt
- Obtain pigs from sources with known health status
- All-in-all-out system
- Using gates to stop the unauthorized entry of people to your farm

*Display This Question:*

*If If NOTE:  Farrow to finish farm (Involves all stages of production, raising pigs from breeding through finishing to market weights)   Farrow to wean farm (Involves raisin... q://QID41/SelectedChoicesCount Is Equal to  1*

**Q127. Which of the following control measures do you have at your Farm 2? (*Check all that apply)***

- Wild animal exclusion or control
- Bird exclusion or control
- Rodent control
- Restrict pet entry (no dogs, cats, etc. are allowed in pig barns)

*Display This Question:*

*If If NOTE:  Farrow to finish farm (Involves all stages of production, raising pigs from breeding through finishing to market weights)   Farrow to wean farm (Involves raisin... q://QID41/SelectedChoicesCount Is Greater Than  1*

*Carry Forward Unselected Choices from "Which type of hog farm is the newest of all your hog farms?"*

**Q128. Which of the following structures are present at your hog farms other than your newest hog farm (${Q43/ChoiceGroup/SelectedChoices})? (Check all that apply)**

| | Farrow to finish farm | Farrow to wean farm | Farrow to nursery/feeder farm | Wean-to-finish farm | Finishing farm | Nursery farm (6) | Other, Please specify (7) |
|---|---|---|---|---|---|---|---|
| Signage at the farm entrance | ☐ | ☐ | ☐ | ☐ | ☐ | ☐ | ☐ |
| Cleaning and disinfection station | ☐ | ☐ | ☐ | ☐ | ☐ | ☐ | ☐ |
| Separate clean area (containing clean boots, coveralls) and dirty area (containing dirty boots, coveralls) | ☐ | ☐ | ☐ | ☐ | ☐ | ☐ | ☐ |
| Shower-in-shower-out facility | ☐ | ☐ | ☐ | ☐ | ☐ | ☐ | ☐ |
| Designated parking area for trucks and visitors | ☐ | ☐ | ☐ | ☐ | ☐ | ☐ | ☐ |
| Designated loading and unloading area for pigs | ☐ | ☐ | ☐ | ☐ | ☐ | ☐ | ☐ |
| Truck wash unit | ☐ | ☐ | ☐ | ☐ | ☐ | ☐ | ☐ |

*Display This Question:*

*If If NOTE:  Farrow to finish farm (Involves all stages of production, raising pigs from breeding through finishing to market weights)   Farrow to wean farm (Involves raisin... q://QID41/SelectedChoicesCount Is Greater Than  1*

*Carry Forward All Answers - Displayed & Hidden from "Which of the following structures are present at your hog farms other than your newest hog farm (${q://QID43/ChoiceGroup/SelectedChoices})? (Check all that apply)"*

Note: **"All-In-All-Out"** is a system that keeps animals together in groups matched by age, weight, production stage, and condition. The group is moved into a phase of production together and is moved out of that phase as a group according to a production schedule. When a group moves forward, the facility is completely emptied.)

**Q129. Which of the following protocols do you follow at your other hog farms? (Check all that apply)**

| | Farrow to finish farm | Farrow to wean farm | Farrow to nursery/feeder farm | Wean-to-finish farm | Finishing farm | Nursery farm (6) | Other, Please specify (7) |
|---|---|---|---|---|---|---|---|
| Requires people (yourself, employees, visitors) to change into farm-specific boots and/or coveralls before entering the farm | ▢ | ▢ | ▢ | ▢ | ▢ | ▢ | ▢ |
| Requires people (yourself, employees, visitors) to shower before entering the farm | ▢ | ▢ | ▢ | ▢ | ▢ | ▢ | ▢ |
| Maintains logbook for visitors at farm entry | ▢ | ▢ | ▢ | ▢ | ▢ | ▢ | ▢ |
| Requires vehicle entering the farm to be washed and free from visible dirt | ▢ | ▢ | ▢ | ▢ | ▢ | ▢ | ▢ |
| Obtain pigs from sources with known health status | ▢ | ▢ | ▢ | ▢ | ▢ | ▢ | ▢ |
| All-in-all-out system | ▢ | ▢ | ▢ | ▢ | ▢ | ▢ | ▢ |
| Using gates to stop the unauthorized entry of people to your farm | ▢ | ▢ | ▢ | ▢ | ▢ | ▢ | ▢ |

*Display This Question:*

*If If NOTE:  Farrow to finish farm (Involves all stages of production, raising pigs from breeding through finishing to market weights)   Farrow to wean farm (Involves raisin... q://QID41/SelectedChoicesCount Is Greater Than  1*

*Carry Forward All Answers - Displayed & Hidden from "Which of the following structures are present at your hog farms other than your newest hog farm (${q://QID43/ChoiceGroup/SelectedChoices})? (Check all that apply)"*

**Q130. Which of the following control measures do you have at your other hog farms? (Check all that apply)**

| | Farrow to finish farm | Farrow to wean farm | Farrow to nursery/feeder farm | Wean-to-finish farm | Finishing farm | Nursery farm (6) | Other, Please specify (7) |
|---|---|---|---|---|---|---|---|
| Wild animal exclusion or control | ▢ | ▢ | ▢ | ▢ | ▢ | ▢ | ▢ |
| Bird exclusion or control | ▢ | ▢ | ▢ | ▢ | ▢ | ▢ | ▢ |
| Rodent control | ▢ | ▢ | ▢ | ▢ | ▢ | ▢ | ▢ |
| Restrict pet entry (no dogs, cats, etc. are allowed in pig barns) | ▢ | ▢ | ▢ | ▢ | ▢ | ▢ | ▢ |

**End of Block: Multi-farm**

---

**Start of Block: Feedback**

## Please give us your feedback.

**Q131. Will you be interested in learning about how to make a farm-specific biosecurity plan?**

- Yes
- No

*Display This Question:*

*If Will you be interested in learning about how to make a farm-specific biosecurity plan? = Yes*

**Q132. Which of the following forms would you prefer the most for educational material? (Check all that apply)**

- Webinar presentation
- Website with information
- Articles in Hog journals
- Articles in Illinois Pork Producers Association newsletters
- Articles in Illinois Farm Week
- Other, Please Specify ________________

*Display This Question:*

*If Will you be interested in learning about how to make a farm-specific biosecurity plan? = Yes*

After this online survey, we will be developing online educational materials that relate to what we learn from this survey. We will announce through the Illinois Pork Producers Association (IPPA) when these educational materials are available. Thank you for your interest.

**End of Block: Feedback**

---
