# Supplementary material for: Assessing and improving on-farm biosecurity knowledge and practices among swine producers and veterinarians through online surveys and an educational website in Illinois, United States
Source: Front Vet Sci. 2023 Jun 9;10:1167056. doi: 10.3389/fvets.2023.1167056 (PMC10289165; doi:10.3389/fvets.2023.1167056)
Supplement: Supplementary file 2 [file Data_Sheet_2.PDF]

Vet_Biosecurity_Survey

Start of Block: Consent Form

Consent Form

Assessing Biosecurity Knowledge and Practices Among Illinois Veterinarians

Dear Colleagues,
You are being asked to participate in a voluntary research study conducted by Dr. Varga's research group at the **College of Veterinary Medicine.**
Veterinarians play an important role in preventing and controlling the spread of infectious diseases through accurate diagnosis, timely reporting, and awareness of their prevention and control. Our study aims to assess your perception regarding the risk of foreign and endemic animal diseases that might impact Illinois animals. We are also interested in the current biosecurity practices used in your clinical settings, and biosecurity advice you provide to the animal owners. Understanding your practices will help us in developing a tailored biosecurity training program for Illinois animal owners. Study participation requires you to complete a short (about 10 minutes) web-based (online) questionnaire. The survey is **anonymous**, and we will maintain the confidentiality of your information to the extent permitted and required by laws and university policies. No identifying information will be linked to your response. Your de-identified information could be used for future research without additional informed consent.
Your participation is voluntary; you may refuse to participate or discontinue participation at any time. This will involve no penalty or loss of benefits to which you are otherwise entitled; neither will it affect your current or future dealings with the **University of Illinois at Urbana-Champaign or Illinois State Veterinary Medical Association (ISVMA).**
The study is sponsored by the United States Department of Agriculture (USDA), Animal and Plant Health Inspection Service, Veterinary Services, under the National Animal Disease Preparedness and Response Program (NADPRP).
If you have questions about this project, please contact **Dr. Csaba Varga (Principal Investigator)** at 217-480-6983 or cvarga@illinois.edu. If you have any questions about your rights as a participant in this study or any concerns or complaints, please contact the **University of Illinois at Urbana-Champaign Office for the Protection of Research Subjects** at 217-333-2670 or via email at irb@illinois.edu. If you would like to retain a copy for your records, you can print this consent form.

I have read and understood the above consent form. I certify that I am 18 years old or older. By clicking the **"Yes, I consent"** option to enter the survey, I indicate my willingness to voluntarily take part in this study.

Q1.1 **Do you consent to participate in this study?**

- ☐ Yes, I consent
- ☐ No, I do not wish to participate in this study

End of Block: Consent Form

Start of Block: Demographic

Let us talk briefly about you and your profession.

Q2.1 **How would you describe your profession?**

- ☐ Veterinarian in clinical practice
- ☐ Veterinarian in academia (Professors, researchers, veterinary teaching clinicians, etc.)
- ☐ Veterinarian in government (State/federal agencies, U.S. Army Veterinary Corps, etc.)
- ☐ Veterinarian in animal welfare sector (ASPCA, etc.)
- ☐ Veterinary Students
- ☐ Veterinary Technician
- ☐ Allied animal health professional (Non-veterinarians)
- ☐ Veterinarians with another status, Please specify
  ____

Skip To: End of Survey If How would you describe your profession? = Veterinary Students

Skip To: End of Survey If How would you describe your profession? = Veterinary Technician

Skip To: End of Survey If How would you describe your profession? = Allied animal health professional (Non-veterinarians)

Q2.2 **What is your gender?**

- ☐ Male
- ☐ Female
- ☐ Prefer not to say

Q2.3 **When did you complete your veterinary degree (DVM or VMD)? (Select the year range of your graduation)**

- ☐ 1944-1954
- ☐ 1955-1964
- ☐ 1965-1974
- ☐ 1975-1984
- ☐ 1985-1994
- ☐ 1995-2004
- ☐ 2005-2014
- ☐ 2015-2021

Q2.4 **Which additional degree or board certification have you completed? (Check all that apply)**

- ☐ None; my highest degree is DVM (or VMD)
- ☐ Graduate degree (MS, MPH, Ph.D., etc.)
- ☐ Board certification (AVMA's American Board of Veterinary Specialties)
- ☐ Others, Please Specify ____

Q2.5 **Have you received any formal training in biosecurity post DVM graduation?**

- ☐ Yes
- ☐ No

Display This Question:

If Have you received any formal training in biosecurity post DVM graduation? = Yes

Q2.6 **When was your last formal training in biosecurity post DVM graduation? (Specify the year)**
Year (Drop-down list)

▼ 2021 (1) ... 1921 (101)

Display This Question:

If Have you received any formal training in biosecurity post DVM graduation? = Yes

Q2.7 **What type of biosecurity training have you received (e.g. webinars, in-person trainings, CE credit trainings, etc.)? (Please specify)**

____

End of Block: Demographic

Start of Block: Disease Perception and Knowledge: (For all vets)

Moving on let's talk briefly about your disease risk perception and knowledge.

Q3.1 **How important do you consider biosecurity practices for the prevention and control of foreign animal diseases (FADs)?**

(A foreign animal disease (FAD) is a disease that is not currently found in the United States, may have been in the U.S. in the past, but have been eradicated.)

- ☐ Very important
- ☐ Important
- ☐ Neutral
- ☐ Less important
- ☐ Not at all important

Q3.2 **How important do you consider biosecurity practices for the prevention and control of infectious diseases?**

- ☐ Very important
- ☐ Important
- ☐ Neutral
- ☐ Less important
- ☐ Not at all important

Q3.3 **How familiar are you with current guidelines and practices for the prevention and control of foreign animal disease (FAD) outbreaks?**

- ☐ Extremely familiar
- ☐ Moderately familiar
- ☐ Somewhat familiar
- ☐ Slightly familiar
- ☐ Not at all familiar

Q3.4 **How important is developing disease prevention and control plans to prepare for a foreign animal disease (FAD) outbreak?**

- ☐ Very important
- ☐ Important
- ☐ Neutral
- ☐ Less important
- ☐ Not at all important

Q3.5 **How important do you consider disease surveillance and testing for the detection and prevention of infectious diseases?**

- ☐ Very important
- ☐ Important
- ☐ Neutral
- ☐ Less important
- ☐ Not at all important

Q3.6 **What do you think is the likelihood of occurrence of foreign animal disease (FAD) outbreak in the US mainland in the next 3 years?**

- ☐ Very likely
- ☐ Likely
- ☐ Neutral
- ☐ Unlikely
- ☐ Very Unlikely

Display This Question:

If How would you describe your profession? != Veterinarian in government (State/federal agencies, U.S. Army Veterinary Corps, etc.)

Q3.7 **What will be your course of action in case you suspect and/or confirm the diseases below?** ***(Check all that apply)***

	Report to USDA-APHIS- Area-Veterinarians-in-Charge (AVIC)	Report to State Animal Health Official (SAHO)	Report to State Public Health Official	Will treat the animal and not report to anyone	I don't know
Foot and Mouth Disease (FMD)					
Equine Influenza					
Porcine Respiratory and Reproductive Syndrome (PRRS)					
African Swine Fever (ASF)					
Rabies					
Leptospirosis					
Salmonellosis					

Display This Question:

If How would you describe your profession? != Veterinarian in government (State/federal agencies, U.S. Army Veterinary Corps, etc.)

Q3.8 **Which type of foreign animal disease (FAD) and State/National reportable disease cases would you report to the State Animal Health Official (SAHO) ?**

- ☐ Suspected cases
- ☐ Confirmed cases
- ☐ Both

Q3.9 **What is the likelihood of a veterinarian transmitting an infectious disease from one animal to another?**

- ☐ Very likely
- ☐ Likely
- ☐ Neutral
- ☐ Unlikely
- ☐ Very unlikely

Q3.10 **How practical is it to follow effective biosecurity measures while handling animals in day-to-day practice?**

- ☐ Very practical
- ☐ Practical
- ☐ Neutral
- ☐ Less practical
- ☐ Not at all practical

End of Block: Disease Perception and Knowledge: (For all vets)

Start of Block: Disease investigations and reporting: (For all practicing vets)

We are almost there! Share with us your experience with disease investigation.

Q5.1 **Have you ever been part of a foreign animal disease (FAD) (suspected/diagnosed) investigation in your career as a practicing veterinarian in Illinois?**

- ☐ Yes
- ☐ No

Q5.2 **Have you suspected/diagnosed any notifiable animal disease in the past 3 years in Illinois?**
(Notifiable animal diseases are the diseases that are required by law to be reported to official

authorities and include all diagnosed or suspected cases of a communicable animal disease for which APHIS has a control or eradication program.)

- ☐ Yes
- ☐ No

Display This Question:

If Have you suspected/diagnosed any notifiable animal disease in the past 3 years in Illinois?(Notif... = Yes

Q5.3 **Who did you report the suspected/diagnosed case of the notifiable animal disease? *(Check all that apply)***

- ☐ No one
- ☐ State Animal Health Official (SAHO)
- ☐ USDA - APHIS- Area veterinarian-in-charge (AVIC)
- ☐ State Public Health Agency
- ☐ Don't remember
- ☐ Others, Please Specify

End of Block: Disease investigations and reporting: (For all practicing vets)

Start of Block: Practice demographic (For Practicing vets only)

Let's talk briefly about your veterinary clinical practice.

Q6.1 **How many years of experience do you have as a practicing veterinarian?**

- ☐ 1-5 years
- ☐ 6-10 years
- ☐ 11-15 years
- ☐ 16-20 years
- ☐ 21-25 years
- ☐ >25 years

Q6.2 **Are you currently a licensed veterinarian in Illinois?**

- ☐ Yes
- ☐ No

Skip To: End of Block If Are you currently a licensed veterinarian in Illinois? = No

Q6.3 **In which Illinois region are you currently practicing clinical veterinary medicine?**

Northeast region counties- Cook, DuPage, Grundy, Kane, Kankakee, Kendall, Lake, McHenry, Will.

North-Central region counties- Boone, Bureau, Carroll, DeKalb, Fulton, Henderson, Henry, Jo Daviess, Knox, LaSalle, Lee, Livingston, Marshall, McDonough, McLean, Mercer, Ogle, Peoria, Putnam, Rock Island, Stark, Stephenson, Tazewell, Warren, Whiteside, Winnebago, Woodford.

Central region counties -Adams, Brown, Calhoun, Cass, Champaign, Christian, Clark, Clay, Coles, Crawford, Cumberland, De Witt, Douglas, Edgar, Effingham, Fayette, Ford, Greene, Hancock, Iroquois, Jasper, Jersey, Lawrence, Logan, Macon, Macoupin, Mason, Menard, Montgomery, Morgan, Moultrie,

Piatt, Pike, Richland, Sangamon, Schuyler, Scott, Shelby, Vermilion.
Southern region counties -Alexander, Bond, Clinton, Edwards, Franklin, Gallatin, Hamilton, Hardin, Jackson, Jefferson, Johnson, Madison, Marion, Massac, Monroe, Perry, Pope, Pulaski, Randolph, Saline, St. Clair, Union, Wabash, Washington, Wayne, White, Williamson.

- ☐ Northeast
- ☐ North-Central
- ☐ Central
- ☐ Southern

Q6.4 **How would you describe your role at the veterinary practice where you are currently practicing clinical veterinary medicine?**

- ☐ Owner/ Co-owner
- ☐ Associate Veterinarian
- ☐ Other, Please Specify ____

Q6.5 **How would you describe the veterinary practice you own or are associated with?**

- ☐ **Companion animal exclusive/ predominant** (Sum of Canine, Feline, Avian (non-poultry) and Exotics) is at between 50-90% of the time spent)
- ☐ **Swine exclusive/ predominant** (Combination of swine predominant and exclusive where there's at least 50% time spent with swine)
- ☐ **Bovine exclusive/ predominant** (Combination of bovine predominant and exclusive where there's at least 50% time spent with bovine)
- ☐ **Equine exclusive/ predominant** (Combination of equine predominant and exclusive where there's at least 50% time spent with equines)
- ☐ **Other species** (Combination of other animal species like caprine, ovine, cervids, camelids, poultry etc.)

End of Block: Practice demographic (For Practicing vets only)

Start of Block: Mixed Animal

We are halfway through! So, you deal primarily with varied animal species. Share with us your clients' awareness about biosecurity and the biosecurity practices you follow in your profession everyday.

Q7.1 **Which of the following animal type do you treat at your practice? (Check all that apply)**

- ☐ Caprine/ovine
- ☐ Camelids
- ☐ Cervids
- ☐ Poultry
- ☐ Others, Please Specify

Q7.2 **How many farm animal clients do you have? (Specify their number)**

____

Q7.3 **How would you describe the overall biosecurity knowledge of your clients?**

- ☐ Extremely knowledgeable
- ☐ Moderately knowledgeable
- ☐ Somewhat knowledgeable
- ☐ Slightly knowledgeable
- ☐ Not at all knowledgeable

Q7.4 **How would you describe the general biosecurity practices followed at your clients' farms?**

- ☐ Excellent
- ☐ Very Good
- ☐ Good
- ☐ Fair
- ☐ Poor

Q7.5 **Do your clients ask you for biosecurity-related advice?**

- ☐ Always
- ☐ Often
- ☐ Sometimes
- ☐ Rarely
- ☐ Never

Q7.6 **Have you provided farm biosecurity assessments for your clients in the past 3 years?**

- ☐ Yes
- ☐ No

Display This Question:

If Have you provided farm biosecurity assessments for your clients in the past 3 years? = Yes

Q7.7 **How many biosecurity assessments have you provided in the past 3 years for your clients? (Specify the number)**

____

Q7.8 **What do you think is the most common biosecurity problem at Illinois livestock farms? (Write in few words)**

____

Q7.9 **When do you provide biosecurity assistance to your clients?**

- ☐ During every consultation
- ☐ Only when they ask
- ☐ During a disease outbreak on their own/nearby farm
- ☐ Never

Q7.10 **Which of the following biosecurity practices do you follow during a <u>routine visit to your clients' farms</u>?**

(Routine visits to the farm are made to evaluate the overall health status of the animal, and include services like regular vaccination administration, checking for pregnancies, and treating sick animals)

	**Always**	**Sometimes**	**Rarely**	**Never**
Wash hands between animals/barns				
Change disposables (like gloves, shoe covers, etc.) between barns				
Change/disinfect boots between barns				
Change coveralls between barns				
Practice shower-in-shower-out, if available				
Moving from healthy animal to sick animal for examination				
Disinfect examination tools (Stethoscope, thermometer, etc.) between barns				
Discard medical waste safely				

Q7.11 **Do you visit multiple farms/sites in a day?**
(Consider swine farms or sites with a separate Premise ID as multiple farms/sites)

☐ Yes
☐ No

Display This Question:

If Do you visit multiple farms/sites in a day?(Consider swine farms or sites with a separate Premise... = Yes

Q7.12 **Which of the following biosecurity practices do you follow for routine visits to multiple farms/sites in a day?**
(Routine visits to the farm are made to evaluate the overall health status of the animal, and include services like regular vaccination administration, checking for pregnancies, and treating sick animals)

	**Always**	**Sometimes**	**Rarely**	**Never**
Use farm-specific coveralls (if available) or change coveralls between farms				
Use farm-specific boots (if available) or change/disinfect boots between farms				
Shower between 2 consecutive farms visits				
Moving from more to less susceptible farms				
Disinfect examination tools (Stethoscope, thermometer, etc.) between farms				

Q7.13 **Do farm owners ask you to follow biosecurity protocols (e.g. changing of coveralls, disposables, etc.) during your visits on their farm?**

☐ Always
☐ Often
☐ Sometimes
☐ Rarely
☐ Never

Q7.14 **Do you carry medical waste disposal can in the vehicle used during farm visits?**

☐ Yes

☐ No

Q7.15 **Do you wash the vehicle used for farm visits?**

☐ Yes

☐ No

Display This Question:

If Do you wash the vehicle used for farm visits? = Yes

Q7.16 **When do you wash the vehicle used for farm visits?**

☐ Between two farm visits

☐ At the end of each workday

☐ Only when it gets visibly dirty

Q7.17 **Do you disinfect the vehicle used for farm visits?**

☐ Yes

☐ No

Display This Question:

If Do you disinfect the vehicle used for farm visits? = Yes

Q7.18 **When do you disinfect wash the vehicle used for farm visits?**

☐ Between two farm visits

☐ At the end of each workday

☐ Only when it gets visibly dirty

End of Block: Mixed Animal

Start of Block: For Swine vets only:

We are halfway through! So, you deal primarily with swine. Share with us your clients' awareness about biosecurity and the biosecurity practices you follow in your profession everyday.

*

Q8.1 **How many swine producer clients do you have?** ***(Specify their number)***

____

Q8.2 **How would you describe the overall knowledge of your swine producer clients towards biosecurity?**

- ☐ Extremely knowledgeable
- ☐ Moderately knowledgeable
- ☐ Somewhat knowledgeable
- ☐ Slightly knowledgeable
- ☐ Not at all knowledgeable

Q8.3 **How would you describe the general biosecurity practices followed at the swine farms of your clients?**

- ☐ Excellent
- ☐ Very Good
- ☐ Good
- ☐ Fair
- ☐ Poor

Q8.4 **Do your swine producer clients ask you for biosecurity-related advice?**

- ☐ Always
- ☐ Often
- ☐ Sometimes
- ☐ Rarely
- ☐ Never

Q8.5 **Have you provided farm biosecurity assessments for your clients in the past 3 years?**

- ☐ Yes
- ☐ No

Display This Question:

If Have you provided farm biosecurity assessments for your clients in the past 3 years? = Yes

Q8.6 **How many biosecurity assessments have you provided in the past 3 years for your swine producers' clients? *(Specify the number)***

____

Q8.7 **What do you think is the most common biosecurity problem at Illinois swine farms? *(Write in few words)***

____

Q8.8 **When do you provide biosecurity advice to your clients?**

- ☐ During every consultation
- ☐ Only when they ask
- ☐ During a disease outbreak on their own/nearby farm
- ☐ Never

Q8.9 **Which of the following biosecurity practices do you follow during a <u>routine visit to each swine farm?</u>**

(Routine visits to the farm are made to evaluate the overall health status of your swine, and include services like regular vaccination administration, processing and castrating piglets, checking for pregnancies, and treating sick pigs)

	Always	Sometimes	Rarely	Never
Wash hands between barns				
Change disposables (like gloves, shoe covers, etc.) between barns				
Change/disinfect boots between barns				
Change coveralls between barns				
Practice shower-in-shower-out, if available				
Moving from healthy animal to sick animal for examination				
Disinfect examination tools (Stethoscope, thermometer, etc.) between barns				

Discard medical waste safely	

Q8.10 **Do you visit multiple swine farms/sites in a day?**
(Consider swine farms or sites with a separate Premise ID as multiple farms/sites)

- ☐ Yes
- ☐ No

Display This Question:

If Do you visit multiple swine farms/sites in a day? (Consider swine farms or sites with a separat... = Yes

Q8.11 **Which of the following biosecurity practices do you follow for routine visits to <u>multiple swine farms/sites in a day</u>?**
(Routine visits to the farm are made to evaluate the overall health status of your swine, and include services like regular vaccination administration, processing and castrating piglets, checking for pregnancies, and treating sick pigs)

	**Always**	**Sometimes**	**Rarely**	**Never**
Use farm-specific coveralls (if available) or change coveralls between farms				
Use farm-specific boots (if available) or change/disinfect boots between farms				
Shower between 2 consecutive farms visits				
Moving from more to less susceptible farms				
Disinfect examination tools (Stethoscope, thermometer, etc.) between farms				

Q8.12 **Do swine producers ask you to follow biosecurity protocols (e.g. changing of coveralls, shower-in-shower-out, etc.) during your farm visits?**

- ☐ Always
- ☐ Often
- ☐ Sometimes
- ☐ Rarely
- ☐ Never

Q8.13 **Do you carry medical waste disposal can (or trash bag) in the vehicle used during farm visits?**

- ☐ Yes
- ☐ No

Q8.14 **Do you wash the vehicle used during farm visits?**

- ☐ Yes
- ☐ No

Display This Question:

If Do you wash the vehicle used during farm visits? = Yes

Q8.15 **When do you wash the vehicle used during farm visits?**

- ☐ Between two farm visits
- ☐ At the end of each workday
- ☐ Only when it gets visibly dirty

Q8.16 **Do you disinfect the vehicle used during farm visits?**

- ☐ Yes
- ☐ No.

Display This Question:

If Do you disinfect the vehicle used during farm visits? = Yes

Q8.17 **When do you disinfect the vehicle used during farm visits?**

- ☐ Between two farm visits
- ☐ At the end of each workday
- ☐ Only when it gets visibly dirty

Q8.18 **Please indicate the occurrence of the following diseases at your clients' swine farms in 2020.**

	Never	Rare	Common	Very common
Porcine Reproductive and Respiratory Syndrome (PRRS)	○	○	○	○
Porcine Epidemic Diarrhea Virus (PEDV)	○	○	○	○
Swine Influenza	○	○	○	○
Colibacillosis	○	○	○	○
Seneca Valley Virus	○	○	○	○
Rotavirus	○	○	○	○
Mycoplasmosis	○	○	○	○
Others, please specify	○	○	○	○

Q8.19 **If a foreign animal disease (FAD) outbreak will occur on a swine farm, what do you think the government indemnity payment approach would be?**

☐ No indemnity payments will be available

☐ Indemnity payments will be available for all farmers regardless of their disease prevention and control efforts

☐ Indemnity payment will be available only for farmers who demonstrate and document disease prevention and control efforts

End of Block: For Swine vets only:

Start of Block: Feedback: (For all vets)

Just feedback!

Q4.1 **From where do you get and update your knowledge on biosecurity?** ***(Check all that apply)***

- ☐ Veterinary and animal health associations
- ☐ Scientific literature
- ☐ Government agencies (e.g. USDA)
- ☐ Meetings and conferences
- ☐ Webinars or seminars
- ☐ Vet specific websites or Online web search
- ☐ Printed media (e.g. magazines, journals, newsletter)
- ☐ From fellow vets
- ☐ Other, Please specify ____

Q4.2 **Which of the following ways would you prefer the most to receive educational materials?** ***(Check all that apply)***

- ☐ Webinar presentation
- ☐ Website with information
- ☐ Articles in journals
- ☐ Articles in ISVMA newsletter
- ☐ Articles in Vet magazines
- ☐ Other, Please Specify ____

End of Block: Feedback: (For all vets)
