# Supplementary material for: Assessing and improving on-farm biosecurity knowledge and practices among swine producers and veterinarians through online surveys and an educational website in Illinois, United States
Source: Front Vet Sci. 2023 Jun 9;10:1167056. doi: 10.3389/fvets.2023.1167056 (PMC10289165; doi:10.3389/fvets.2023.1167056)
Supplement: Supplementary file 3 [file Data_Sheet_3.PDF]

“Test your knowledge Questions”

Module 1: Swine Diseases

Q1. Which of the following is a Foreign Animal Disease in the US?
- African Swine Fever
- Classical Swine Fever
- Foot and Mouth Disease
- All the above

Q2 Which of the following signs indicate a healthy pig? (Check all that applies)
- Alert, bright eyes
- Alert, pointed ears with no visible swelling or injury
- Deviated, dry nose with discharges or lesions
- No visible bones on body and no back arching
- Dull, drooping tail visible lesions

Q3

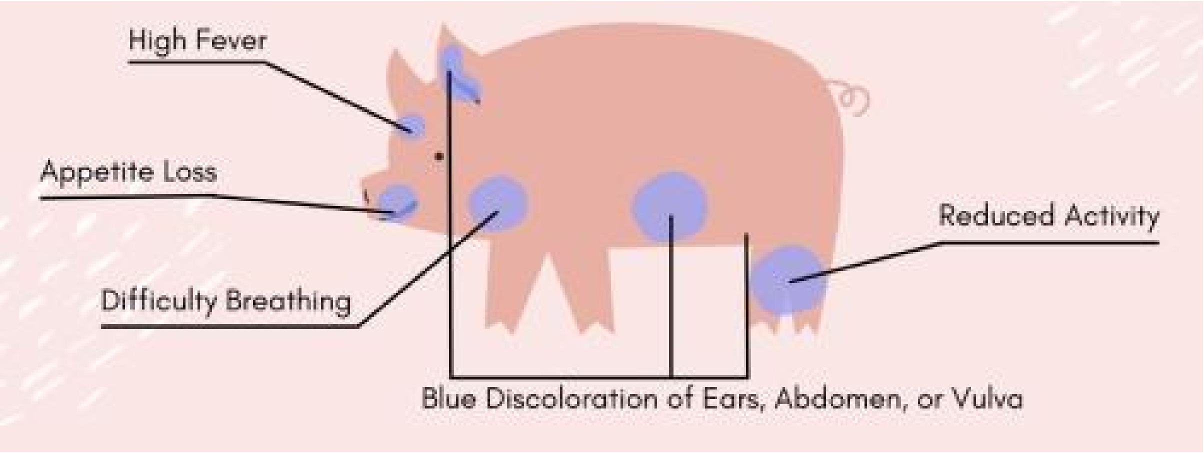

Which of the following disease typically shows the symptoms shown in the above picture?
- African Swine Fever (ASF)
- Porcine Epidemic Diarrhoea (PED)
- Porcine Reproductive and Respiratory Syndrome (PRRS)
- Foot and Mouth disease (FMD)

Q4 True or False: Foot and mouth disease vaccination is used in swine in the US.
- True
- False

Q5 In which year Porcine Epidemic Diarrhoea (PED) was first reported in the US swine herd?
- 2009
- 2018
- 2013
- 1994

Q6 Swine infectious diseases can be transmitted by-

- Direct contact between infected and susceptible animals
- Exposure to contaminated feed
- Vector bites (i.e., tick, lice)
- All the above

Module 2: Biosecurity Overview

Q1 **Biosecurity refers to-**

- Practices to prevent the introduction of disease into the farm
- Practices to prevent spread of disease within/between farm(s)
- Both
- None

Q2 **True or False: Biosecurity is only for commercial swine farms.**

- True
- False

Q3 **Which of the following statement is TRUE about biosecurity? (Check all that apply)**

- Biosecurity helps in prevention of disease on a farm
- Biosecurity is a one-time-single step process
- Biosecurity is a protective shield against new emerging diseases
- Biosecurity is not required at finisher pig farms

Q4 **True or False: Biosecurity protocols are disease-specific.**

- True
- False

Module 3: Biosecurity Practices

Q1 **True or False: Signs are an inexpensive way to make visitors aware of farm rules and biosecurity practices.**

- True
- False

Q2 **LOS in terms of biosecurity stands for-**

- Line of Supply
- Length of Stay
- Line of Separation
- Limits of Stability

Q3 **Identify the Perimeter Buffer Area (PBA) in the given picture.**

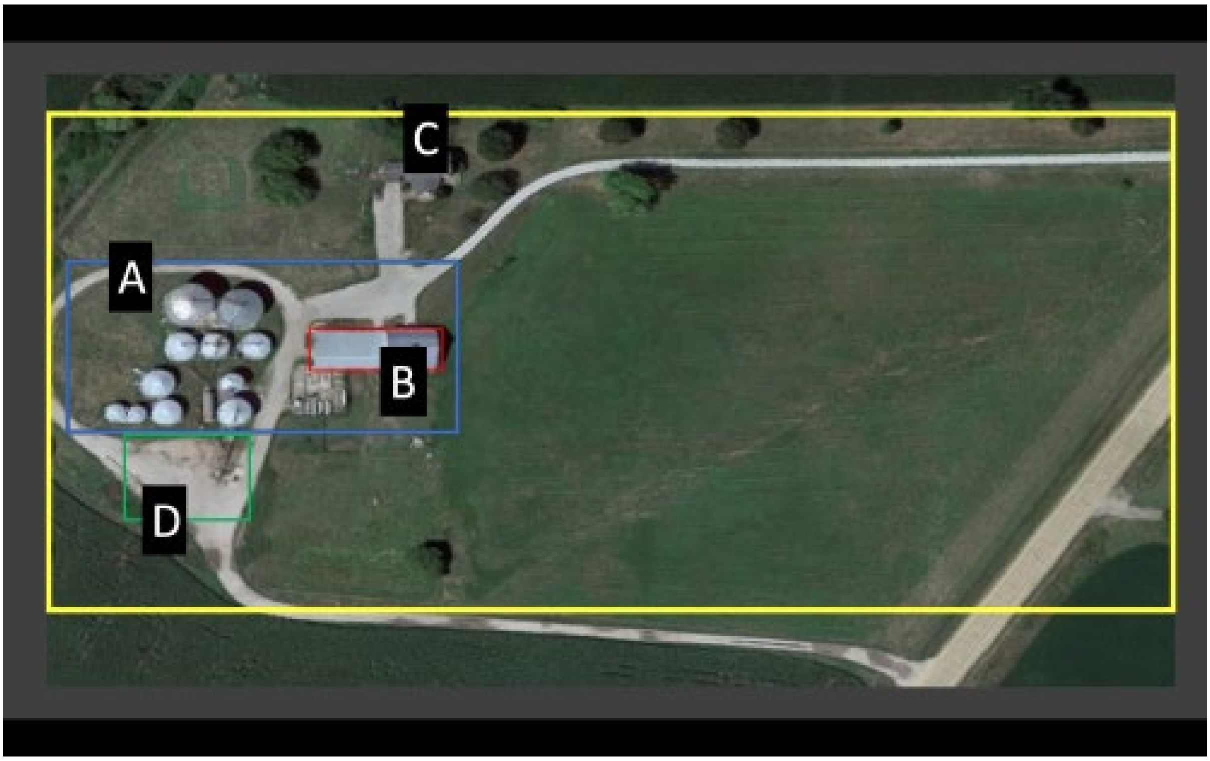

- A
- B
- C
- D

Q4 **True or False: Gates and fences are not important barriers in open housing systems on swine farms.**

- True
- False

Module 4: External Biosecurity

Q1 **Which of the following are elements of external biosecurity?**

- Truck cleaning and disinfection
- Quarantining new incoming stock
- Both
- None

Q2 **Which of the following information one must collect for the visitors' log? (Check all that apply)**

- Name
- Contact Information (including vehicle information)
- Foreign Travel History
- Sex
- Contact with livestock in past 48-72 hours
- Medical Insurance information

Q3 **The most bio-secure way of entering a swine farm is:**

- Showering and changing into farm specific clothes and boots for entering the farm
- Only changing into farm specific clothes and boots before entering the farm
- Only changing boots and washing hands before entering the farm
- Only wearing disposables clothes and boots before entering the farm

Q4 **Recommended duration of quarantining new incoming stock before introducing in the existing pig herd is-**

- 10 days
- 90-120 days
- 30-40 days
- 3 days

Q5 **True or False: A clear clean and dirty area demarcation should be followed during the loading and unloading of animals.**

- True
- False

Module 5: Internal Biosecurity

Q1 **True or False: The movement of employees within the farm should be from young to adult pigs.**

- True
- False

Q2 **Which of the following is the most efficient technique to control air-borne diseases like PRRS?**

- Installing air filtration system
- Mechanical ventilation system
- Natural ventilation system

Q3 **True or False: Biosecurity training should be provided only to permanent employees of a swine farm.**

- True
- False

Q4 **Which of the following activity should be done at the end of a working day?**

- Working in farrowing barn
- Working at nursary barn
- Disposing dead animals
- Checking gilts for heat signs

Module 6: Secure Pork Supply Plan

Q1 **Do you need a Premise ID for getting a Secure Pork Supply (SPS) Plan?**

- Yes
- No
- Optional

Q2 **Who of the following can conduct a biosecurity assessment for a swine farm? (Check all that apply)**

- Veterinarian
- Biosecurity Manager
- Feed mill manager
- Experienced Swine Producer
- Any swine producer

Q3 **What is the main purpose of having a Secure Pork Supply Plan?**

- To have a farm biosecurity plan
- To ensure continuity of business in an event of foreign animal disease outbreak
- To provide insurance to farm animals
