# Supplementary material for: Assessing and improving on-farm biosecurity knowledge and practices among swine producers and veterinarians through online surveys and an educational website in Illinois, United States
Source: Front Vet Sci. 2023 Jun 9;10:1167056. doi: 10.3389/fvets.2023.1167056 (PMC10289165; doi:10.3389/fvets.2023.1167056)
Supplement: Supplementary file 4 [file Data_Sheet_4.PDF]

BIOSECURITY CHECKLIST

VARGA LAB
INFECTIOUS DISEASE EPIDEMIOLOGY

College of Veterinary Medicine
UNIVERSITY OF ILLINOIS URBANA-CHAMPAIGN

- ☐ Mark each access point with a sign and protect it with a suitable barrier.
- ☐ Define a designated parking spot for non-farm vehicles on the farm premises.
- ☐ Restrict entry on the farm premises only to authorized vehicles and people.
- ☐ Maintain a log-book entry for visitors and vehicles entering the farm premises.
- ☐ Establish clean and dirty areas with visible demarcation lines at the farm entrance.
- ☐ Require everyone entering the farm to shower and/or change into farm-specific boots and coveralls.
- ☐ Restrict entry of pork products onto the farm premises.

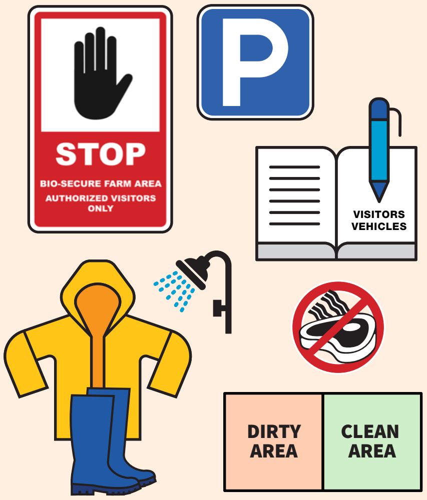

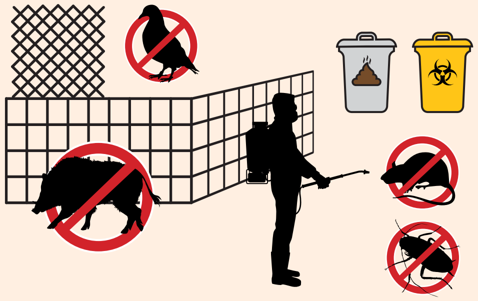

- ☐ Set up exclusion measures to limit wild animals, birds, and pets from entering the farm premises.
- ☐ Implement vector-control measures for pests like rodents, flies, ticks, and lice.
- ☐ Develop manure and mortality disposal plans.
- ☐ If pick-up services are used, keep garbage and dead animal disposal bins outside the farm premises.

- ☐ Clean and disinfect farm equipment, machinery, and transport vehicles after use.
- ☐ Clean and disinfect barns and feeders between animal groups.
- ☐ Test water regularly if supply comes from on-farm wells or surface water.
- ☐ Flush and disinfect waterlines and drinkers if necessary.
- ☐ Follow unidirectional movement while performing farm operations (e.g. young to adult pigs, healthy to sick pigs).

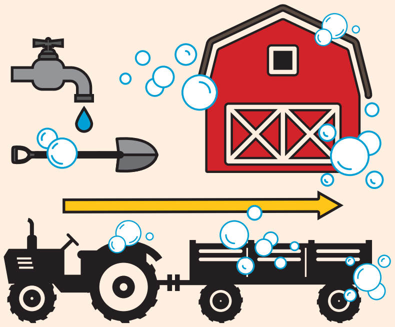

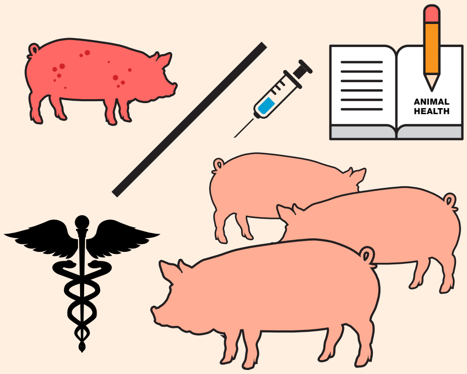

- ☐ Separate incoming pigs for 30-40 days from existing pigs on the farm.
- ☐ Routinely monitor the health of all pigs and isolate sick pigs immediately.
- ☐ Vaccinate pigs as per their vaccination schedule.
- ☐ Appoint a veterinarian for herd health and farm biosecurity management.
- ☐ Provide biosecurity training to all farm employees.
- ☐ Routinely evaluate and update the farm biosecurity protocols if needed.

BIOSECURITY PROTOCOL *for* ENTERING A PIG FARM

VARGA LAB
INFECTIOUS DISEASE EPIDEMIOLOGY

College of Veterinary Medicine
UNIVERSITY OF ILLINOIS URBANA-CHAMPAIGN

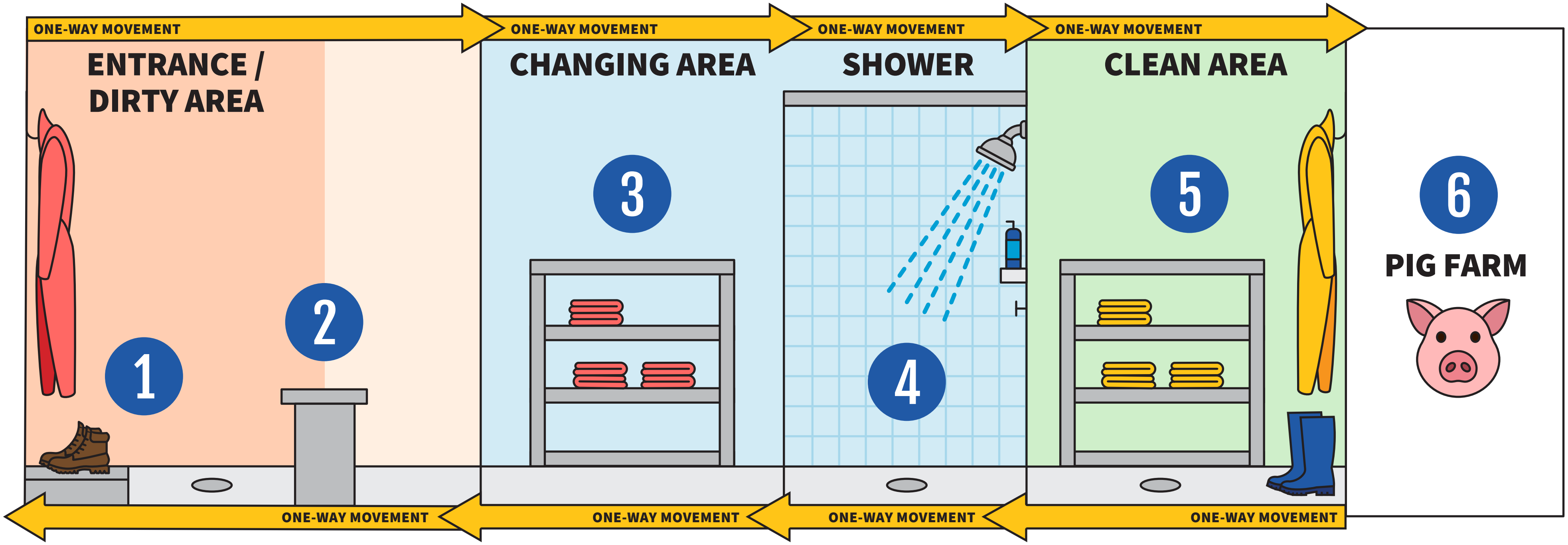

1. Remove your shoes (along with disposable booties, if any) and coat and place them on the designated shoe and coat rack at the entrance.
2. Cross the bench without touching your feet on the dirty side of the entrance floor.
3. Enter the changing area, remove all your clothes, place them on the clothing rack, and step into the shower.*
4. Take a shower and wash your hair using soap and shampoo.
5. Step into the designated clean area of the pathway and put on the farm-specific boots and coveralls.**
6. Enter the pig farm.

NOTE: All the movement should be one-way with no turning back in between.

* If showering facility is unavailable, change into farm-specific clothes and boots.
** Disposables can also be worn if farm-specific coveralls and boots are not available.

SPS CHECKLIST

VARGA LAB
INFECTIOUS DISEASE EPIDEMIOLOGY

College of Veterinary Medicine
UNIVERSITY OF ILLINOIS URBANA-CHAMPAIGN

The **Secure Pork Supply (SPS)** plan is intended for swine producers to ensure business continuity in the event of a foreign animal disease outbreak as well as protect the farm operations from endemic diseases.

What do you need to implement an SPS plan?

- ☐ Premise Identification Number (PIN)
- ☐ Site-specific enhanced biosecurity plan
- ☐ Farm records (including movement records, delivery, visitors, etc.)
- ☐ Biosecurity training for the farm personnel
- ☐ Education on foreign animal disease signs and symptoms to facilitate disease monitoring
- ☐ Facilities for sample collection (oral fluid, nasal swab, and blood) in the event of an outbreak

How do you get an SPS plan?

STEP 1 **Familiarize yourself with the SPS at secureport.org**

STEP 2 **Validate your premises; obtain a PIN; print a map**

STEP 3 **Locate and compile records**: Logbooks, Certificates of Veterinary Inspection (CVIs), and Standard Operating Procedures (SOPs)

STEP 4 **Write and implement an enhanced biosecurity plan** (template below)
https://www.securepork.org/pork-producers/biosecurity/

STEP 5 **Create and label a premises map** (detailed description below)
https://www.securepork.org/Resources/SPS_CreatingPremisesMap-AnimalsRaisedIndoors.pdf

STEP 6 **Have written biosecurity protocols**

STEP 7 **Complete the Illinois Enhanced Biosecurity Checklist:**

For indoor animal rearing:
https://www.securepork.org/Resources/SPS_Biosecurity_Self-Assessment_Checklist-_-IndoorProduction.pdf

For animals with outdoor access:
https://www.securepork.org/Resources/SPS-Biosecurity-Checklist-for-Animals-with-Outdoor-Access.pdf

STEP 8 **Complete Foreign Animal Disease Training and Response Resource:**
https://www.securepork.org/training-materials/

For more detailed information on the eight steps go to:
https://www2.illinois.gov/sites/agr/Animals/AnimalHealth/Documents/Secure%20Pork%20Supply%20Plan%20Eight%20Step%20to%20Participate%20for%20Illinois%20Producers.pdf

LOADING & UNLOADING PIGS BIOSECURELY

VARGA LAB
INFECTIOUS DISEASE EPIDEMIOLOGY
College of Veterinary Medicine
UNIVERSITY OF ILLINOIS URBANA-CHAMPAIGN

Loading and unloading pigs is a major threat to farm biosecurity. Stringent biosecurity measures must be followed to prevent disease introduction into the farm.

LOCATION

A designated area must be constructed for loading and unloading pigs. It should be at least 65-165 feet away from pig housing.

DESIGN of the loading/unloading area and chute:

- The ramp should have a slope of about 5°.
- The chute should be constructed to facilitate only the unidirectional flow of animals.
- Lines should be demarcated between clean and unclean areas to prevent physical contact between farmworkers and transport vehicle and its driver and movement from one side to another of the demarcation line must be prohibited.

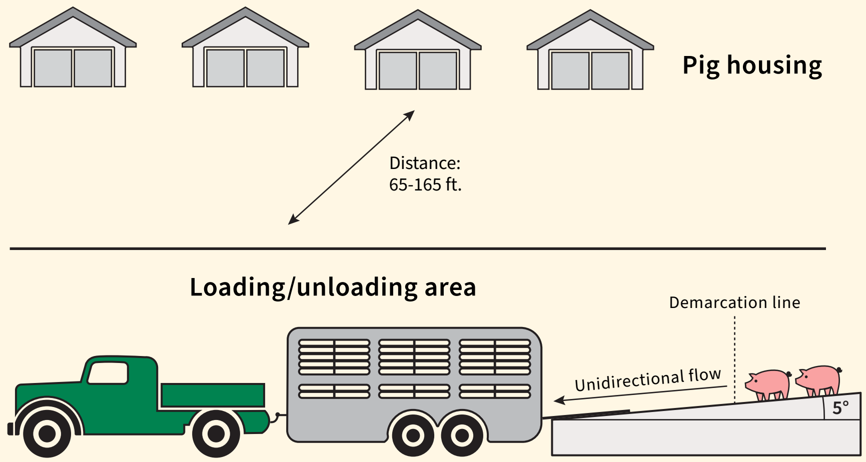

LOADING

BEFORE LOADING

The transport driver must . . .

- Ensure that the transport vehicle used for loading the animals is empty, clean, and disinfected before arrival on the farm.
- Inform the farm office about their arrival in advance and inform them bout the cleanliness status of the vehicle to the farm manager.
- Follow farm biosecurity protocols while entering the farm and park the trailer only in the loading and unloading area.
- Change into clean coveralls and boots from his/her biosecurity kit before starting the loading process.

The farm manager must . . .

- Ensure the loading chute is clean and disinfected.

DURING LOADING

The transport driver must . . .

DIRTY AREA
CLEAN AREA

- Climb in the trailer and identify and establish the demarcation between clean and dirty areas and ensure not crossing the demarcation line at any point during the process.
- Line the trailer and chute with bedding material to avoid injury to the animals due to slipping.

Farmworkers and the transport driver must . . .

- Not cross the demarcated clean and dirty line during loading process.
- Avoid direct contact with pigs by using a board or stick to guide their movement.
- Prevent pigs from turning and moving back into the clean area.
- Maintain records of loaded pigs.

AFTER LOADING

The transport driver must . . .

- Secure the gates of the transport vehicle properly.
- Change coveralls and boots and store them in the dirty bin before entering the cab of the trailer. Dispose of the disposables in a trash bag in the cab.

The farm manager must . . .

- Instruct the employees to clean and disinfect the loading/unloading chute and hallway before using it for another loadout or load-in.

TRANSPORTING

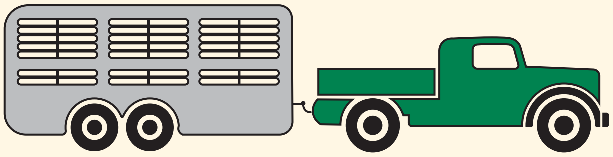

DURING TRANSPORT

The transport driver must:

- Choose the traveling route that has low pig farm density.
- Avoid bumpy trails.
- Check on animals from outside of the trailer at frequent intervals.

UNLOADING

BEFORE UNLOADING

The transport driver must . . .

- Change into clean coveralls and boots from his/her biosecurity kit before starting the unloading process.
- Climb in the trailer and identify and establish the demarcation between clean and dirty areas and ensure not crossing the line at any point during the process.

The farm manager must . . .

- Ensure the unloading chute is clean and disinfected.

DURING UNLOADING

farmworkers and the transport driver must . . .

- Avoid direct contact with loading pigs, use a board or stick to guide their movement.
- Prevent pigs from turning and moving back into the trailer from the clean area.
- Maintain records of unloaded pigs.

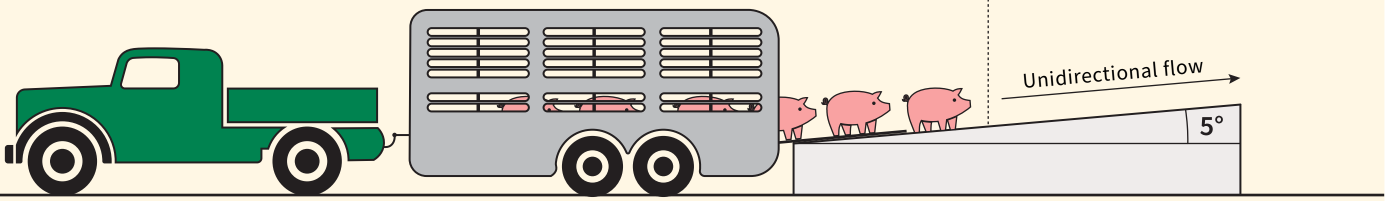

AFTER UNLOADING

The transport driver must . . .

- Sign the required paperwork.
- Remove the dirty boots and coveralls and store them in the dirty bin and send it for cleaning and disinfection.
- Clean and disinfect the trailer and allow it to dry before the next delivery.

The farm manager must . . .

- Instruct the employees to clean and disinfect the loading/unloading chute and hallway before using it for another loadout or load-in. The newly arrived animals must be held in a quarantine room for a minimum of 30-40 days before mixing with the existing stock.

PROTECT *YOUR FARM* **AGAINST**

AFRICAN SWINE FEVER

VARGA LAB
INFECTIOUS DISEASE EPIDEMIOLOGY
College of Veterinary Medicine
UNIVERSITY OF ILLINOIS URBANA-CHAMPAIGN

WHAT is ASF?

African Swine Fever (ASF) is a **high-consequence viral disease** capable of causing massive production and financial losses to the US swine industry. It is a foreign animal disease and is not currently present in the United States.

HOW is ASF transmitted?

A healthy pig can get infected by coming in **direct contact** with an infected pig or infected wild boar or with a person who has been in direct contact with an infected pig. Visitors traveling from countries where ASF is present could pose a risk by bringing in infected pork products.

WHY should swine producers worry about ASF?

Even though ASF is not found in the US, the chance of its introduction to the US is possible through live animal trade or international travel. Currently, **ASF was detected in the Dominican Republic**, and if ASF enters Puerto Rico or the US Virgin Islands, the pork trade of the entire nation will be affected.

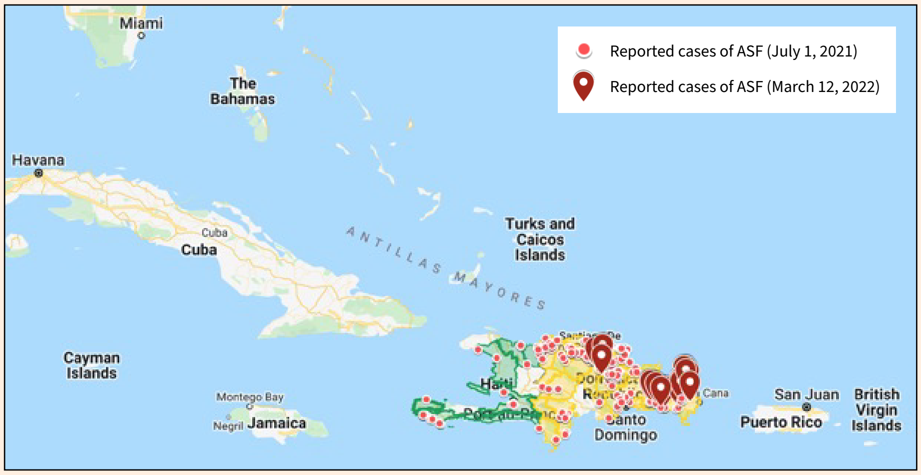

WHAT can I do to protect my pig herd from ASF?

There is no vaccine or treatment available against ASF. The only way to prevent ASF is by preventing its introduction into your farm. This can be achieved by implementing **farm-level biosecurity:**

- Require everyone entering the farm to shower and/or change into farm-specific boots and coveralls.
- Do not permit visitors with a history of international travel within the past 90 days, especially to countries where ASF is present.
- Avoid importing live pig feed from regions or countries where ASF is present.
- Do not feed swill/ kitchen waste to pigs .
- Prevent contact with wild boars by fencing the farm and providing indoor housing.
- Implement vector-control measures for pests like rodents, flies, ticks, and lice.
- Clean and disinfect equipment and transport vehicles after use.
- Buy pigs with known disease status and quarantine incoming stock.

For more swine biosecurity information and a biosecurity checklist visit:
https://vetmed.illinois.edu/swine-biosecurity

WHEN should I suspect ASF?

Know the **signs** and **symptoms** of the disease and contact your veterinarian immediately if you find any of these signs:

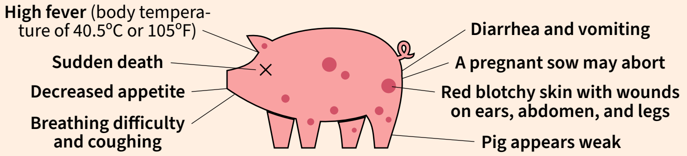

For more symptom information visit:
https://www.fdacs.gov/Consumer-Resources/Animals/Animal-Diseases/African-Swine-Fever

Infected pigs die within 6-13 days after the onset of symptoms; however, sometimes pigs die suddenly without showing any symptoms of the disease.

WHO should I report to if I suspect ASF?

If you see similar symptoms in your herd and suspect ASF, immediately contact your veterinarian or report it to the **State Animal Health Department** (217)-782-4944.

VARGA LAB
INFECTIOUS DISEASE EPIDEMIOLOGY

College of Veterinary Medicine
UNIVERSITY OF ILLINOIS URBANA-CHAMPAIGN

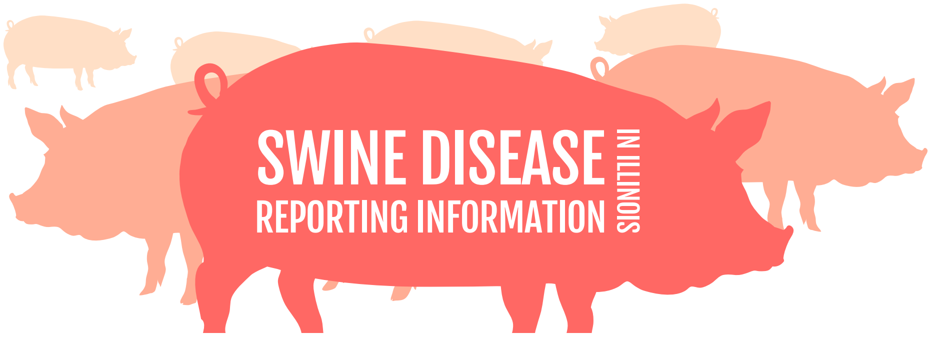

For Animal Owners

When To Suspect A Foreign Animal Disease ?

- High morbidity and/or mortality
- High abortion rates of unknown etiology
- Severe respiratory conditions
- Vesicular condition of any type
- Poor to no response to conditions when response is anticipated
- Recent foreign travel, foreign visitors, or receipt of foreign parcels
- History of importation of animals, embryos, or semen
- Undiagnosed central nervous system conditions
- Presence of exotic flies, mites, ticks, larvae
- Unusual or unexplained signs of illness
- Atypical findings at necropsy

If an animal owner suspects a foreign animal disease or any unusual disease symptoms in their herd, they must contact their veterinarian immediately.

For more swine biosecurity information visit: **https://vetmed.illinois.edu/swine-biosecurity**

Who Must Report ?

Veterinarians
Diagnostic Laboratories
Other Animal Health Professionals

What To Report ?

Any suspected or confirmed case of the diseases listed below or unusually high morbidity or mortality in a herd or diseases not known to exist in the United States.

Who To Report To ?

PRIMARY CONTACT

- **SAHO — State Animal Health Official**
  Illinois Department of Agriculture, Bureau of Animal Health and Welfare
  (217) 782-4944 ■○✚▲

ADDITIONAL CONTACTS

- USDA — **United States Department of Agriculture** — APHIS Veterinary Services Illinois District Office — **(217) 547-6030** ○▲
- SPHO — **State Public Health Official** — Illinois Department of Public Health, Division of Infectious Disease — **(217) 785-7165** ✚▲
  Local health department after hours:
  (800) 782-7860 or **(217) 782-7860**
  https://idph.illinois.gov/LHDMap/HealthRegions.aspx →

Key for Reportable Diseases: ■ Reportable to **SAHO** ○ Reportable to **SAHO** & USDA ✚ Reportable to **SAHO** & SPHO ▲ Reportable to **SAHO**, USDA & SPHO

Foreign Animal Diseases

Animal owners can consult their veterinarians in case they suspect any of these diseases.

- ○ African swine fever
- ▲ Anthrax
- ○ Foot-and-mouth disease
- ○ Hog cholera (Classical swine fever)
- ○ Nipah virus encephalitis
- ○ Porcine cysticercosis
- ○ Swine vesicular disease
- ○ Teschovirus encephalomyelitis

Notifiable Animal Diseases

These diseases *are required to be reported by accredited veterinarians* and not animal owners.

- ▲ Brucellosis
- ○ Louping ill
- ○ Porcine babesiosis
- ○ Pseudorabies
- ▲ Rabies
- ■ Senecavirus A
- ■ Trichinellosis
- ○ Vesicular stomatitis

Diseases of Public Health Importance

These are diseases that are transmitted from animals to humans *are a risk to human health* (zoonotic diseases).

- ▲ Brucellosis
- ✚ Campylobacteriosis
- ✚ Cryptosporidiosis
- ✚ Leptospirosis
- ✚ Pathogenic E. coli
- ✚ Q fever
- ▲ Rabies
- ✚ Salmonellosis

INFECTION PREVENTION + CONTROL *in* VETERINARY CLINICS

VARGA LAB
INFECTIOUS DISEASE EPIDEMIOLOGY

College of Veterinary Medicine
UNIVERSITY OF ILLINOIS URBANA-CHAMPAIGN

Suspecting an Infectious Disease?

Take the patient to a dedicated ISOLATION ROOM.
(If you do not have one, convert an examination room into an isolation room.)

Isolation room must...

1. Be in a low human and animal traffic area of the clinic, and a separate entrance is preferable.
2. Be physically separated from the other examination rooms to reduce the risk of direct or indirect transmission.
3. Be restricted to authorized personnel only.
4. Contain limited equipment and supplies. (Excess supplies should not be stored in an isolation room.)
5. Ventilation should be separate from the rest of the clinic. If that is not possible, a EPA air filtration system should be used.
6. Have clear signage indicating RESTRICTED AREA/BIO SECURE AREA/ISOLATION AREA.
7. Be cleaned and disinfected between patients.

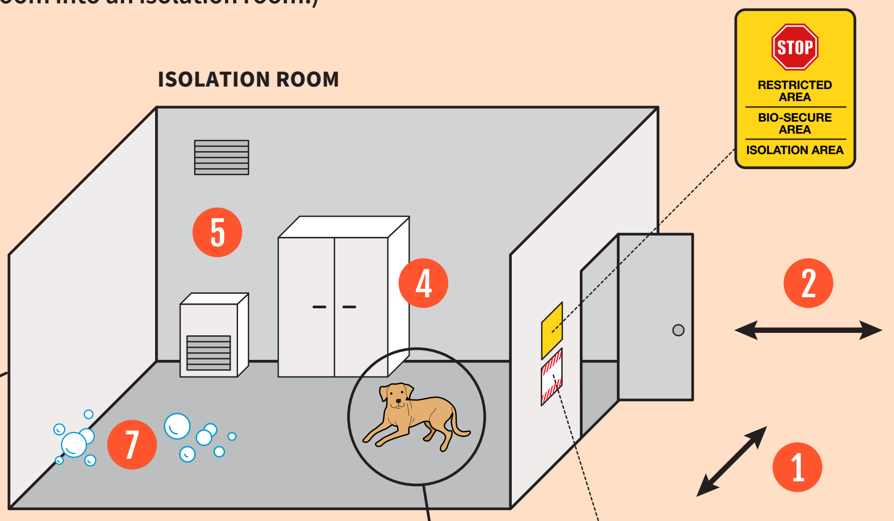

NOTE: All items and supplies entering an occupied isolation room must be considered infectious and should be properly disinfected or disposed of after every patient.

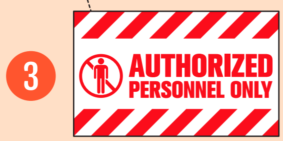

Personnel entering the Isolation Room must...

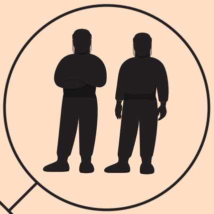

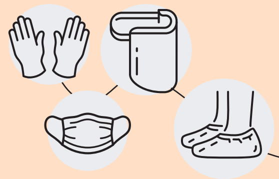

Wear appropriate protective clothing/personal protective equipment either disposable or specific for the isolation room including gloves, shoe cover, face, and eye shield (depending on the infectious agent).

Discard all disposables securely after a single-use .

Replace any protective equipment contaminated with infected bodily fluid.

Wash and sanitize hands before putting on gloves and after removing them.

Handling the patient:

- Dogs in isolation rooms should not be walked or allowed to urinate or defecate in the area used by other animals.
- A separate area for each dog should be designated for walking, urination, and defecation. DO NOT use the same area for all animals in an isolation area.
- Dog handlers can carry the dog or transport it on a gurney wearing a PPE kit. This is ideal for minimizing contamination of the clinic environment.
- STOP The walking area must have clear signage to avoid unauthorized entry.
- Common diagnostic facilities must be used at the end of the day or during minimum traffic hours to limit transmission.
- All facilities used outside of the isolation room must be cleaned and disinfected thoroughly before using for other animals.
- PPE All personnel handling, caring for, or treating the animal suspected of infectious disease must wear PPE when with the patient.

Education & Training

Every Clinic or hospital must have an Infection Control Program, a written protocol, and an Infection Control Officer to monitor it.

All doctors and staff should be vaccinated for Rabies, Influenza, and tetanus.

All staff members must be given proper infection prevention and control training.

The pet owners of animals suspected or diagnosed with a zoonotic or infectious disease must be given clear instructions on how to handle the animal and protect themselves from infections.

Pet owners of animals admitted for infectious diseases must not be allowed to visit their pets.

Reporting

The following suspected or confirmed cases must be reported to the **State Animal Health Official (SAHO)**:

- Rabies
- Tularemia
- Echinococcosis
- Rabbit Haemorrhagic Five
- Plague
- Brucellosis
- Avian influenza
- Psittacosis (ornithosis)
- Piroplasmosis
- Pullorum disease
- Q fever
- Salmonellosis
- Leptospirosis

A list of reportable diseases and reporting procedures should be displayed in the clinical complex for easy access to the clinic's personnel.

Any accidental animal bite incidence during examination or handling must be reported to the **State Public Health Official (SPHO)**. (Note the vaccination status of the animal.)

***Animals from shelter should be considered as high risk for infectious diseases and must be handled with appropriate precautions.**

CONTACT:

SAHO — State Animal Health Official
Illinois Department of Agriculture,
Bureau of Animal Health and Welfare
(217) 782-4944

SPHO — State Public Health Official
Illinois Department of Public Health,
Division of Infectious Disease
(217) 785-7165
Local health department after hours:
(800) 782-7860 or **(217) 782-7860**
