# Supplementary material for: Assessing and improving on-farm biosecurity knowledge and practices among swine producers and veterinarians through online surveys and an educational website in Illinois, United States
Source: Front Vet Sci. 2023 Jun 9;10:1167056. doi: 10.3389/fvets.2023.1167056 (PMC10289165; doi:10.3389/fvets.2023.1167056)
Supplement: Supplementary file 5 [file Data_Sheet_5.PDF]

Supplementary Table 1. The description of questions from the "Test your knowledge" quizzes of each module, along with the frequency of the total, correct, and incorrect responses per question.

Modules	Total Responses	Correct Responses	Incorrect Responses
**Swine diseases**	**12**		
Q1. Which of the following is a Foreign Animal Disease in the US?	8	4	4
Q2 Which of the following signs indicate a healthy pig?	9	5	4
Q3 Which of the following disease typically shows the symptoms shown in the above picture?	9	2	7
Q4 True or False: Foot and mouth disease vaccination is used in swine in the US.	9	8	1
Q5 In which year Porcine Epidemic Diarrhea (PED) was first reported in the US swine herd?	9	3	6
Q6 Swine infectious diseases can be transmitted by?	9	9	0
**Biosecurity overview**	**26**		
Q1 Biosecurity refers to?	16	15	1
Q2 True or False: Biosecurity is only for commercial swine farms.	17	17	0
Q3 Which of the following statement is TRUE about biosecurity?	17	14	3
Q4 True or False: Biosecurity protocols are disease-specific.	17	14	3
**General biosecurity practices**	**0**		
Q1 True or False: Signs are an inexpensive way to make visitors aware of farm rules and biosecurity practices.	0	0	0
Q2 LOS in terms of biosecurity stands for-	0	0	0
Q3 Identify the Perimeter Buffer Area (PBA) in the given picture.	0	0	0
Q4 True or False: Gates and fences are not important barriers in open housing systems on swine farms.	0	0	0
**External biosecurity**	**7**		
Q1 Which of the following are elements of external biosecurity?	6	6	0
Q2 Which of the following information one must collect for the visitors' log?	6	2	4
Q3 The most bio-secure way of entering a swine farm is?	6	6	0
Q4 Recommended duration of quarantining new incoming stock before introducing it in the existing pig herd is?	6	6	0
Q5 True or False: A clear clean and dirty area demarcation should be followed during the loading and unloading of animals.	6	6	0
**Internal biosecurity**	**6**		
Q1 True or False: The movement of employees within the farm should be from young to adult pigs.	6	6	0

Q2 Which of the following is the most efficient technique to control air-borne diseases like PRRS?	6	5	1
Q3 True or False: Biosecurity training should be provided only to permanent employees of a swine farm.	6	6	0
Q4 Which of the following activity should be done at the end of a working day?	6	6	0
**Secure Pork Supply plan**	**7**		
Q1 Do you need a Premise ID for getting a Secure Pork Supply (SPS) Plan?	6	6	0
Q2 Who of the following can conduct a biosecurity assessment for a swine farm?	6	6	0
Q3 What is the main purpose of having a Secure Pork Supply Plan?	6	4	2
